# Chemoenzymatic conversion of amides to enantioenriched alcohols in aqueous medium

**DOI:** 10.1038/s42004-019-0182-8

**Published:** 2019-07-19

**Authors:** Jacob E. Dander, Maude Giroud, Sophie Racine, Evan R. Darzi, Oscar Alvizo, David Entwistle, Neil K. Garg

**Affiliations:** 1 Department of Chemistry and Biochemistry, University of California, Los Angeles, CA 90095, USA.; 2 Codexis, Inc., 200 Penobscot Drive, Redwood City, CA 94070, USA.; 3 These authors contributed equally: Maude Giroud, Sophie Racine.

## Abstract

One-pot reactions that combine non-enzymatic and biocatalytic transformations represent an emerging strategy in chemical synthesis. Some of the most powerful chemoenzymatic methodologies, although uncommon, are those that form a carbon–carbon (C–C) bond and a stereocenter at one of the reacting carbons, thereby streamlining traditional retrosynthetic disconnections. Here we report the one-pot, chemoenzymatic conversion of amides to enantioenriched alcohols. This transformation combines a nickel-catalyzed Suzuki–Miyaura coupling of amides in aqueous medium with an asymmetric, biocatalytic reduction to provide diarylmethanol derivatives in high yields and enantiomeric excesses. The synthetic utility of this platform is underscored by the formal syntheses of both antipodes of the pharmaceutical orphenadrine, which rely on ketoreductase enzymes that instill complementary stereoselectivities. We provide an explanation for the origins of stereoselectivity based on an analysis of the enzyme binding pockets.

The blending of traditional synthetic methods and biocatalytic transformations represents an increasingly important approach to building chemical architectures^1–5^. One variant of this strategy incorporates a biocatalytic step(s) into a multistep synthetic campaign, often to exploit the exquisite regio-, chemo-, or stereoselectivity introduced by an enzyme (Fig. 1a)^6–9^. Another opportunity in this area lies in the development of methodologies that merge a nonenzymatic step with a biocatalytic cycle in a one-pot fashion^10–13^. Such chemoenzymatic methodologies are highly attractive, as they leverage the unique strengths of both reaction classes and, importantly, enable streamlined approaches to achieve multistep functional group conversions. Successful strategies for achieving transformations of this type include the integration of whole-cell metabolism with biocompatible metal-catalysis^14,15^ and in vitro combinations of organo- or metal-catalyzed steps with biocatalytic transformations^5^. The value of both nonenzymatic and enzymatic processes is well established, with enzymatic processes becoming increasingly important because of their mild reaction conditions, sustainability, and impressive selectivities^1,2,5,11–13,16,17^. Rapid advancements in protein engineering technologies, the topic of the 2018 Nobel Prize in chemistry, have resulted in chemoenzymatic syntheses of pharmaceuticals^2,6–8^ and natural products^9,18,19^. Notable examples of chemoenzymatic syntheses of pharmaceuticals that have benefited humanity include the preparation of Sitagliptin, Singulair, and Lipitor, the latter of which is the best-selling drug of all time (Fig. 1a)^2,8^.

Several chemoenzymatic transformations have now been developed and are the subject of reviews^10–13^. From a synthetic standpoint, we view those that allow for the formation of a new carbon–carbon (C–C) bond and lead to the formation of an associated stereocenter as being highly desirable. This notion is consistent with the teachings of Corey in the context of retrosynthetic analysis^20^. Indeed, many widely used nonenzymatic transformations, such as asymmetric aldol reactions^21,22^ and 1,4-additions^23,24^ proceed in such a manner. Additionally, many biocatalysts such as aldolases^25,26^ and polyketide synthases^27^ have evolved to achieve such transformations, highlighting the importance of this strategy in biosynthesis. The chemoenzymatic alternative is to mechanistically decouple the bond forming and stereocenter generating steps. An example of this scenario is where C–C bond formation occurs in a nonenzymatic process, followed by the generation of a stereocenter. Although there have been several reports describing transformations of this type, the majority accomplish C–C bond formation and stereocenter generation at carbon atoms distal to one another, as opposed to at the same carbon atom(s)^10^. Reactions of the latter type are viewed as highly enabling, resembling venerable chemocatalytic transformations that are structurally constructive and stereoselective^20^. Despite this, few methodology advances have been reported in this regard. Hartwig and Zhao disclosed a cooperative Ru-catalyzed metathesis–biocatalytic epoxidation sequence using a biphasic compartmentalization strategy in 2014 (Fig. 1b)^28^. A subsequent study from the same laboratories reported a related system for the cooperative synthesis of styrenyl epoxides^29^. More recently, these groups have disclosed a chemoenzymatic synthesis of 2-aryl-succinate derivatives using a Rh-catalyzed diazo-coupling–enereductase sequential reaction manifold^30^.

With the aforementioned notions in mind, we sought to develop the chemoenzymatic transformation outlined in Fig. 1c. Amides **1** would undergo conversion to enantioenriched alcohols **2** through the combination of a nickel-catalyzed Suzuki–Miyaura amide coupling^31–33^ and a ketoreductase (KRED)-mediated asymmetric reduction^34,35^. This manifold incorporates several noteworthy design elements: (a) The reactions would utilize amides **1** as substrates, which have been traditionally avoided as building blocks in organic synthesis because of their pronounced resonance stabilization^36^. (b) Nonprecious metal catalysis would be used to cleave the classically inert amide C–N bond and build a new C–C bond^37–39^. The use of nickel, in particular, is attractive due to potential economic, environmental, and toxicological benefits relative to precious metal alternatives^37,40,41^. (c) Aryl boronates **3** were envisioned to be ideal reacting partners, given that Suzuki–Miyaura cross-couplings provide robust synthetic tools and a mild platform for C–C bond formation^42,43^. (d) KREDs would be utilized to reduce ketone intermediates **4** and generate the newly formed stereocenter in **2**, as protein engineering and commercialization have established KREDs as a gold standard for asymmetric carbonyl reductions, including cases where the ketone substituents are remarkably similar^44,45^. (e) The success of this endeavor would facilitate the preparation of enantioenriched diarylmethanols, a motif reminiscent of scaffolds commonly encountered in bioactive molecules and pharmaceuticals such as **5** and **6**^46^. It should be noted that no enantioselective conversions of amides to other functional groups using catalytic C–N bond cleavage^47,48^ have been reported to date, despite the rapid growth of metal-catalyzed amide couplings in recent years^37–39^.

Herein we report the conversion of amides to enantioenriched alcohols. Key to the success of our efforts is the surprise finding that the Suzuki–Miyaura coupling of amides can be achieved using an aqueous medium. Moreover, we have identified engineered KREDs that lead to high enantioselectivities in the biocatalytic reduction. When leveraged in a one-pot sequence, diarylmethanol derivatives are obtained in high yields and enantiomeric excesses. The formal syntheses of both enantiomers of the pharmaceutical orphenadrine, using complementary KREDs, underscores the synthetic utility of this chemoenzymatic transformation. An analysis of the enzyme binding pockets delineates the structural origins of observed stereoselectivities. The transformation described herein presents an example of a one-pot chemoenzymatic methodology that forges a C–C bond and provides for the controlled generation of an associated stereocenter in good yields and enantiomeric excesses up to 99%. This study sets the stage for the development of tandem variants of the described transformation in the future and validates chemoenzymatic strategies as a means to achieve enantioselective transformations of amides.

## Results

### Challenges in the development of chemoenzymatic methodologies.

There are several inherent challenges associated with developing the desired chemoenzymatic conversion of amides to enantioenriched alcohols. Generally speaking, one-pot, chemoenzymatic reactions are plagued by incompatibilities between the nonenzymatic and enzymatic processes (e.g., solvent, pH, etc.) and various catalyst deactivation pathways^10–13^. Such challenges exist when considering tandem reactions (both catalysts operate concurrently in one-pot) or sequential, stepwise approaches in one-pot, with the latter offering a possible means to circumvent some of the many incompatibilities^10^. Thus, if a tandem process could not be achieved, we would develop a sequential, stepwise transformation, which would enable the desired one-pot transformation and also lay the foundation for the development of even more challenging tandem variants in the long term. We therefore set out to tackle several specific concerns, such as if the Suzuki–Miyaura coupling of amides could be performed under aqueous conditions, if an engineered KRED could be identified to efficiently and stereoselectively reduce the diaryl ketone intermediates accessible from the Suzuki–Miyaura coupling, and, most questionably, if the two transformations could be coupled together without catalyst deactivation.

### Suzuki–Miyaura cross-coupling of amides in aqueous medium.

The first of these concerns, whether the Suzuki–Miyaura coupling step could be performed under aqueous conditions, was initially addressed. This was a notable uncertainty given that several reports exist describing the deleterious impact of excess water on nickel-catalyzed Suzuki–Miyaura couplings^49–52^ with only one reported approach for nickel-catalyzed Suzuki–Miyaura couplings in strictly aqueous medium using Lipshutz’s designer surfactant methodology^53^. On the other hand, if water could be employed in the Suzuki–Miyaura coupling of amides, this would ultimately enable the desired chemoenzymatic process, as the aqueous medium would be important for enzyme activity. The use of water as the medium would also minimize the use of organic solvent, thus affording greener coupling conditions compared to the parent methodology that utilized toluene^31^.

**Fig. 2:**
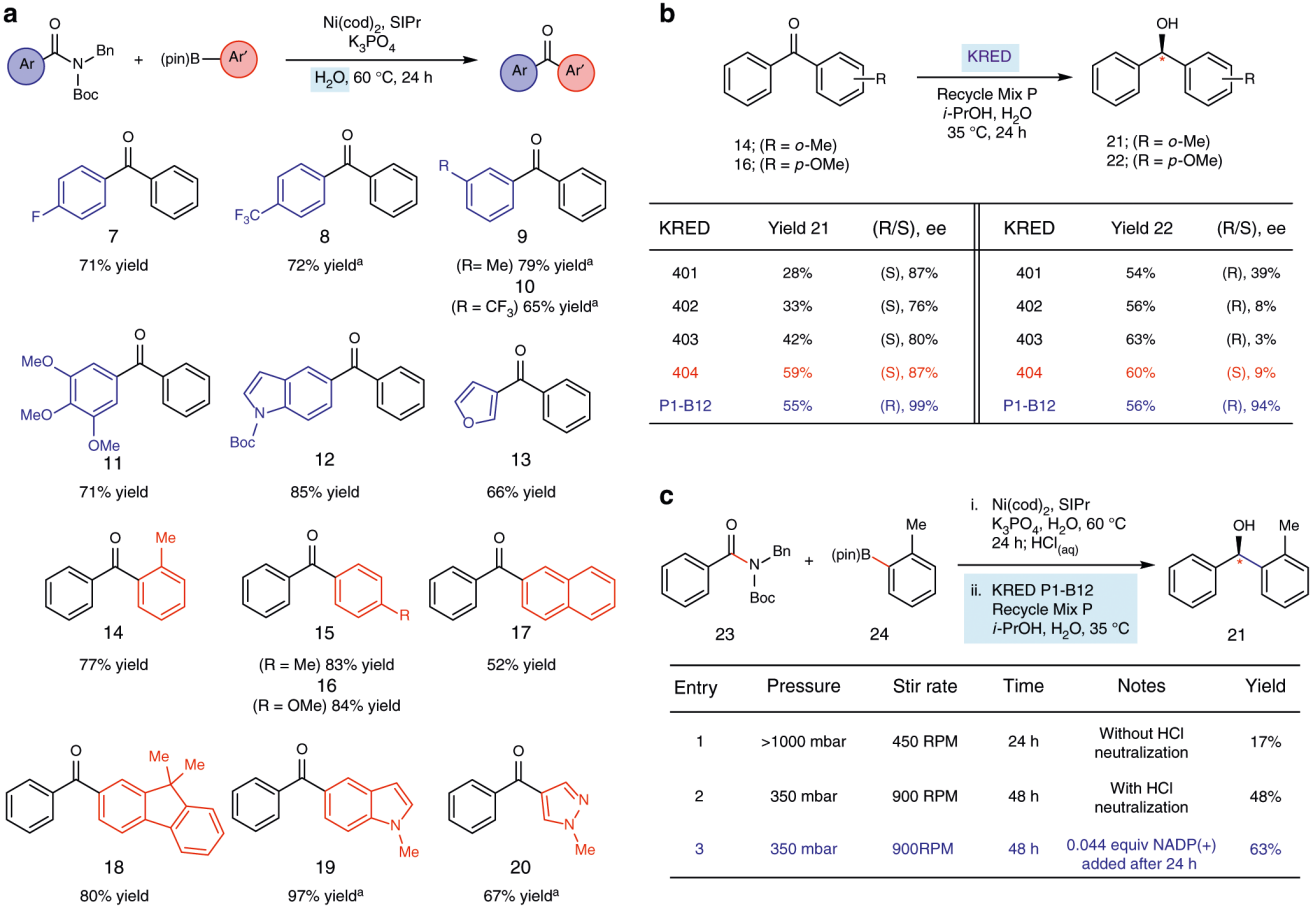
Development of the one-pot chemoenzymatic conversion of amides to enantioenriched alcohols. **a** Scope of the Suzuki–Miyaura coupling of amides in aqueous medium. Conditions: substrate (1.0 equiv), boronate (3.0 equiv), Ni(cod)_2_ (15 mol%), SIPr (30 mol%), K_3_PO_4_ (2.0 equiv), and H_2_O (0.5 M), heated at 60 °C for 24 h. ^a^50 μL toluene additive used in the reaction. Reactions were performed on 0.1 mmol scale and the yields reported reflect the average of two isolation experiments. **b** Evaluation of commercial KREDs in the generation of alcohols **21** and **22**. Conditions: substrate(1.0 equiv), KRED (10 mg/mL), Recycle Mix P (18.2 mg/mL), *i*-PrOH (52 equiv), and H_2_O (0.025 M), heated at 35 °C and stirred at a rate of 450 RPM for 24 h. Reactions were performed on 0.025 mmol scale and yields were determined by ^1^H NMR analysis using 1,3,5-trimethoxybenzene as an external standard. Enantiomeric excesses (ee’s) were determined via chiral Supercritical Fluid Chromatography (SFC). **c** Optimization of the one-pot sequential chemoenzymatic methodology using the coupling of amide **23** and boronate **24**. Conditions (Coupling: step 1): substrate (1.0 equiv), boronate (3.0 equiv), Ni(cod)_2_ (15 mol%), SIPr (30 mol%), K_3_PO_4_ (2.0 equiv), and H_2_O (0.5 M), heated at 60 °C for 24 h, followed by quenching with 1 M HCl. Conditions unless otherwise indicated (Reduction: step 2): KRED (10 mg/mL), Recycle Mix P (18.2 mg/mL), *i*-PrOH (52 equiv), and H_2_O (0.025 M), heated at 35 °C and stirred for 24 h. Reactions were performed on 0.1 mmol scale and the yields were determined by ^1^H NMR analysis using 1,3,5-trimethoxybenzene as an external standard

After surveying a range of reaction conditions (see Supplementary Methods and Supplementary Table 1), we were pleasantly surprised to find that water (0.5 M) could indeed be used as the reaction medium. These conditions deviate from our previously reported conditions in that: (a) the reaction temperature is increased to 60 °C, (b) organic solvent (toluene) is omitted or used sparingly as an additive, (c) the reaction is performed at lower concentration (0.5 M), (d) the catalyst and ligand loadings are increased to 15 mol% and 30 mol%, respectively, and e) the equivalences of boronate employed are slightly increased to 3.0^31^. The scope of this methodology is summarized in Fig. 2a. It should be noted that the reactions are likely occurring “on water,” as complete dissolution of the reaction components is not observed^54^. In a few cases, increased yields were seen if a small portion of toluene was used as a minor additive. Electron-withdrawing substituents on the para position of the amide substrate, such as −F and −CF_3_ were tolerated, as shown by the formation of products **7** and **8**, respectively. Similarly, substrates bearing meta substituents could be used, giving rise to coupled products **9** and **10**. Additionally, it was found that a highly substituted 3,4,5-trimethoxybenzamide underwent coupling to give **11**, and that substrates bearing indole or furan heterocycles could be coupled, as demonstrated by the formation of **12** and **13**, respectively. The reaction was also found to tolerate a range of boronate coupling partners, including an *o*-tolylboronate, which led to ketone **14**, and those bearing para −CH_3_ or −OMe substituents, giving **15** and **16**, respectively. Boronates bound to polycyclic aromatic hydrocarbons performed well in the coupling, as judged by the formation of naphthylone **17** and 9,9-dimethylfluorenyl ketone **18**. Lastly, indolyl and pyrazolyl boronates underwent coupling to afford **19** and **20**, respectively, thus demonstrating that heterocyclic boronates can be utilized in this methodology. Of note, some of the ketone products in Fig. 2a (i.e., **14**, **15**, and **19**) were generated in higher yields using our aqueous conditions compared to our originally disclosed conditions using organic solvent^31^. Collectively, these results suggest that excess water is not detrimental to nickel-mediated Suzuki–Miyaura couplings of amides, and also address one of the key challenges in the development of the desired chemoenzymatic methodology.

### Evaluation of ketoreductases.

Our next effort focused on identifying an evolved KRED that could reduce a range of diaryl ketones of the type obtained via the aforementioned Suzuki–Miyaura coupling of amides. Diaryl ketones are particularly challenging substrates for both chemocatalytic reduction and enzymatic reduction^44,46^. With regard to the latter, Merck has described the use of KRED-100 series enzymes to reduce diaryl ketones, which requires a glucose/glucose dehydrogenase recycling system for the NADPH cofactor^44^. Moreover, a wide range of enantiomeric excesses (ee’s) were reported with no single enzyme prevailing as the enzyme of choice for the reduction of differentially substituted diaryl ketones. We therefore sought to identify an engineered KRED that could reduce a range of diaryl ketones and would rely on a simpler, and more desirable, isopropanol (*i*-PrOH) recycling system for the NADPH cofactor^35,45^.

A focused library of engineered KREDs was identified by limiting the selection of enzymes to those capable of accommodating aryl groups in their small binding pocket. Namely, mutation of the Y190 residue in the *kefir* wild-type sequence to a smaller amino acid is the primary means to expand the small binding pocket and achieve this end. Enzymes featuring such mutations and requiring the preferable isopropanol (*i*-PrOH) recycling system were then experimentally evaluated using ketones **14** and **16**, which served as representative ketones displaying steric and electronic differentiation of the ketone substituents. Results from these studies are shown in Fig. 2b, which highlight that engineered 400 series KREDs and KRED P1-B12 were most promising overall and utilize the preferable *i*-PrOH recycling system for the NADPH cofactor, present in Recycle Mix P^45^. These KREDs share a common evolutionary ancestor in the Wild Type (WT) short-chain aldo-KRED of *Lactobacillus brevis*^55^. Of note, KRED 404^56^ and KRED P1-B12^57^ displayed complementary stereoselectivities, with KRED P1-B12 being superior in terms of enantioselectivities. Specifically, using KRED P1-B12 the (*R*) enantiomers of **21** and **22** were obtained in excellent ee’s of 99 and 94%, respectively. KRED P1-B12 was selected as the enzyme of choice for developing the targeted one-pot chemoenzymatic methodology.

### One-pot, chemoenzymatic methodology.

Having developed the Suzuki–Miyaura coupling using water as the reaction medium and identified promising biocatalytic reduction conditions for diaryl ketones, we pursued the one-pot, chemoenzymatic conversion of amides to enantioenriched alcohols (Fig. 2c). Although the development of a tandem reaction (both catalytic cycles operating concurrently) was viewed as ideal, attempts to achieve a process of this sort were unsuccessful. Thus, by targeting a sequential process, the Suzuki–Miyaura coupling could be performed as already optimized (see Fig. 2a), thus limiting incompatibilities with the biocatalytic reduction step. Both processes could be performed in water, but it was unclear what complications would arise due to the highly basic nature of the Suzuki–Miyaura coupling, and the presence of nickel complexes, the NHC ligand, excess boronate, and minor byproducts. Unfortunately, performing the Suzuki–Miyaura coupling of **23** and **24** with sequential KRED reduction gave a low yield of alcohol **21** (entry 1). However, through a series of troubleshooting and optimization efforts, we ultimately uncovered several experimental tactics that gave **21** in improved yield. For example, simply neutralizing the reaction medium immediately after the Suzuki–Miyaura coupling by the addition of aqueous HCl was beneficial. We also found the stir rate, reaction time, and pressure of the reaction vessel to be critical. Thus, by performing the reaction under reduced pressure, which drives the enzymatic equilibrium forward by removing acetone (see Codex® KRED Screening KitFAQs), and with faster stirring over 48 h to ensure proper mixing, the desired product **21** was obtained in 48% ^1^H NMR yield (entry 2). Lastly, we observed a further boost in ^1^H NMR yield to 63% by adding supplementary NADP(+). In this chemoenzymatic process, at least ten chemical entities are introduced in the reaction vessel, ultimately allowing for the controlled formation of a new C–C bond and an associated stereocenter, thus furnishing alcohol **21** in high ee. Our success in developing a sequential process utilizing both nickel and biocatalysis is a crucial stepping-stone toward the ultimate development of tandem variants of such chemoenzymatic transformations.

**Fig. 3:**
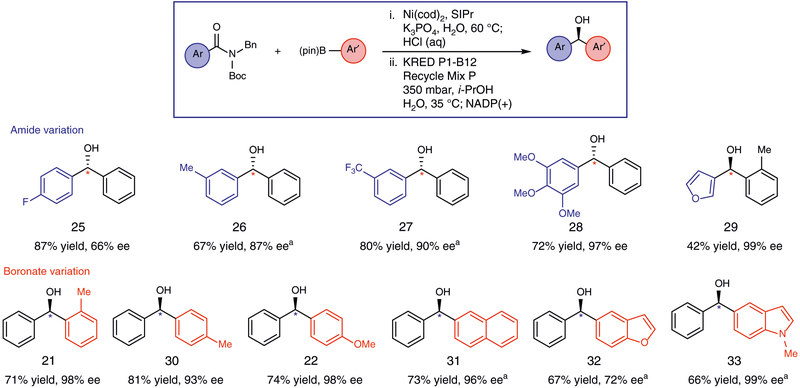
Scope of the one-pot chemoenzymatic conversion of amides to enantioenriched alcohols. Conditions (Coupling: step 1): substrate (1.0 equiv), boronate (3.0 equiv), Ni(cod)_2_ (15 mol%), SIPr (30 mol%), K_3_PO_4_ (2.0 equiv), and H_2_O (0.5 M), heated at 60 °C for 24 h, followed by quenching with 1 MHCl. ^a^50 μL toluene additive used in the reaction. Conditions (Reduction: step 2): KRED (10 mg/mL), Recycle Mix P (18.2 mg/mL), *i*-PrOH (52 equiv), and H_2_O (0.025 M), heated at 35 °C and stirred for 24 h at 350 mbar; Na_2_NADP(+)•3H_2_O (0.044 equiv), heated at 35 °C and stirred for an additional 24 h at 350 mbar. Reactions were run on 0.1 mmol scale and yields were determined by ^1^H NMR analysis using 1,3,5-trimethoxybenzene as an external standard. Reactions were performed on 0.1 mmol scale and the yields reported reflect the average of two isolation experiments. Enantiomeric excesses (ee’s) were determined via chiral SFC

The one-pot, chemoenzymatic methodology was evaluated using various amides and boronates to give a range of enantioenriched alcohol products in synthetically useful isolated yields and good to excellent enantioselectivities (Fig. 3). With regard to the amide coupling partner, we tested a *p*-fluorobenzamide in the coupling with PhB(pin). This gave rise to alcohol **25** in 87% isolated yield and 66% ee. It is notable that KRED P1-B12 is able to somewhat differentiate the two aromatic rings that are similarly sized and vary only by the presence of the fluoride substituent positioned distal from the carbonyl of the presumed ketone intermediate. Substrates bearing *m*-CH_3_ and medicinally privileged *m*-CF_3_ substituents were also examined, ultimately affording alcohols **26** and **27** in 87% and 90% ee, respectively. We also demonstrated that a trimethoxyphenyl substituted substrate could be utilized, without competitive cleavage of the C–O bonds^58^. The resultant alcohol **28** was obtained in 72% yield and 97% ee. Furthermore, an amide featuring a heteroaromatic furan could be coupled, as demonstrated by the formation of alcohol **29** in 99% ee. Various aryl boronate coupling partners were also evaluated with similar success. As noted from our optimization studies, the *o*-tolylboronate could be readily employed in the one-pot process. In isolation experiments, this gave **21** in 71% yield and 98% ee. Para substituents were tolerated, as judged by the efficient formation of **30** and **22** in good yields and excellent enantioselectivities. Of note, the synthesis of **30** constitutes a formal synthesis of (*R*)-neobenodine^59^, an antihistaminic, and anticholinergic drug^46^. Additionally, it was found that boronates featuring extended aromatic systems, such as naphthalene, could be employed in the reaction to generate alcohol **31** in 73% yield and 96% ee. Lastly, we employed both benzofuranyl and indolyl boronic esters in the chemoenzymatic methodology. This resulted in the formation of heterocycle-containing adducts **32** and **33** in good yields and selectivities. The scope and stereoselectivity of this transformation are notable given that prior studies on KRED systems tolerant of diaryl ketones have displayed limited substrate scopes, enantioselectivities, and less desirable cofactor recycling systems^44^.

### Formal syntheses of (*R*)- and (*S*)-orphenadrine.

**Fig. 4:**
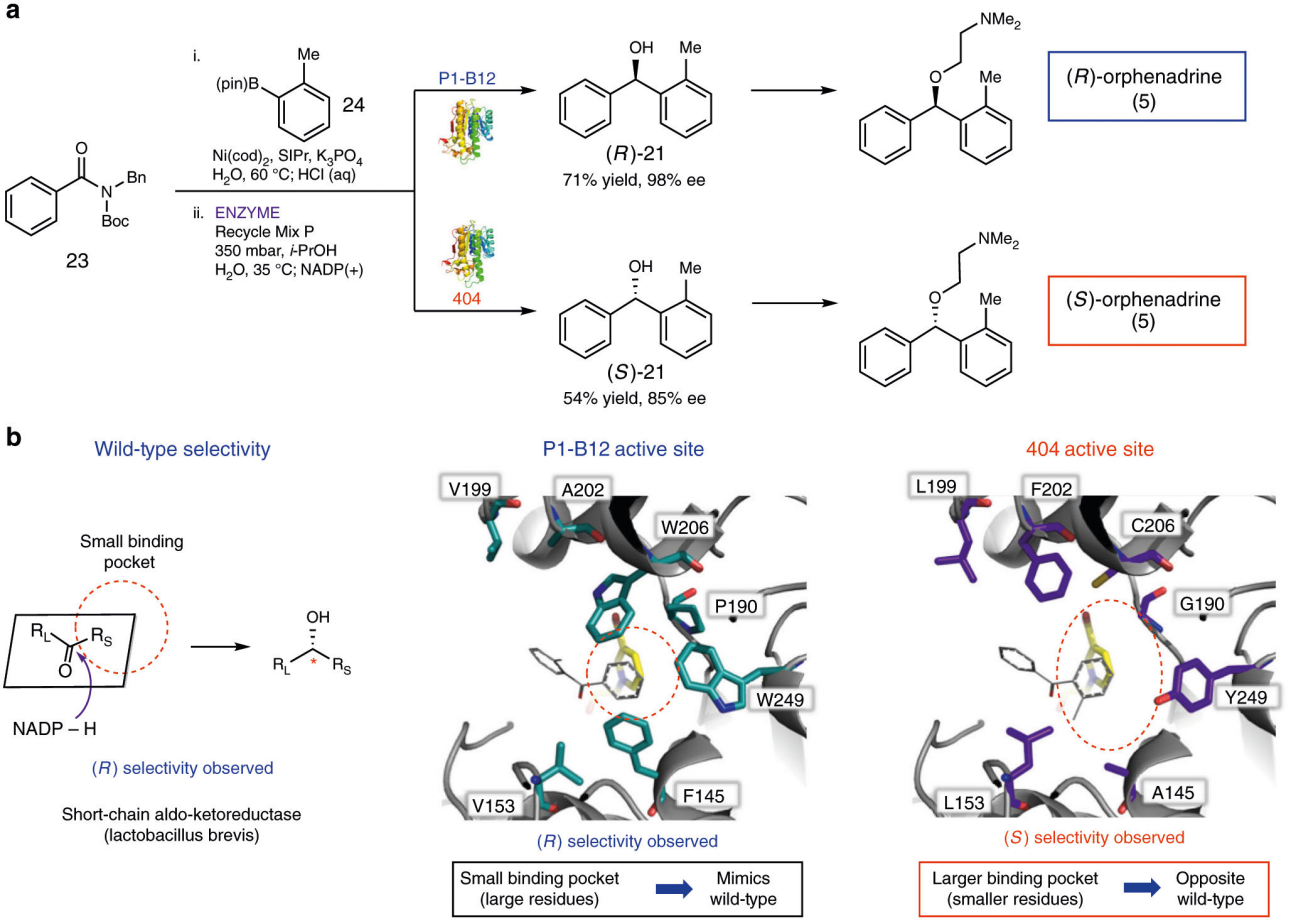
Access to both enantiomers of orphenadrine and model of the origins of stereoselectivity. **a** Formal syntheses of (*R*)- and (*S*)-orphenadrine using the one-pot, chemoenzymatic methodology. Conditions (Coupling: step 1): substrate (1.0 equiv), boronate (3.0 equiv), Ni(cod)_2_ (15 mol%), SIPr (30 mol%),K_3_PO_4_ (2.0 equiv), and H_2_O (0.5 M), heated at 60 °C for 24 h, followed by quenching with 1 M HCl. Conditions (Reduction: step 2): KRED (10 mg/mL), Recycle Mix P (18.2 mg/mL), *i*-PrOH (52 equiv), and H_2_O (0.025 M), heated at 35 °C and stirred for 24 h at 350 mbar; Na_2_NADP(+)•3H_2_O (0.044 equiv), heated at 35 °C and stirred for an additional 24 h at 350 mbar. Reactions were performed on 0.1 mmol scale and the yields reported reflect the average of two isolation experiments. Enantiomeric excesses (ee) were determined via chiral SFC. **b** Analysis of binding pocket and origins of stereoselectivities for the wild-type KRED, KRED P1-B12, and KRED 404 (ketone **14** is docked). Protein coordinates taken from “PDB ID 1ZK4, 1.0 Å resolution” and then used in modeling studies. The ketoreductases are shown in gray and NADPH is shown in yellow. Key residues in the binding pocket are shown in aqua green for P1-B12 and in purple for 404 (nitrogen shown in red, oxygen in blue)

Given that the use of KRED P1-B12 consistently delivers enantioenriched alcohols with the same sense of chirality, reflecting a general limitation of most enzymatic processes, we questioned if an alternate KRED could be used complementarily in the one-pot, chemoenzymatic methodology. This notion was tested in the context of a synthetic application, where we sought to prepare both enantiomers of the antihistamine orphenadrine (**5**, Fig. 4a). Thus, amide **23** and boronic ester **24** were subjected to our typical reaction conditions for the conversion of amides to enantioenriched alcohols, with the only variable being the choice of KRED. Using KRED P1-B12, the (*R*) enantiomer of **21** was obtained in 71% yield and 98% ee as previously noted. However, by switching to KRED 404, the (*S*) enantiomer of **21** was prepared in 54% yield and 85% ee. Alcohol **21** is known to readily undergo alkylation in one additional step to give **5**; thus, the preparation of the (*R*) and (*S*) enantiomers of **21** constitutes formal syntheses of both antipodes of **5**^60^. Given that diarylmethanols are commonly seen in bioactive substances as mentioned earlier, we hope this methodology will enable the preparation of enantioenriched pharmaceutical substances.

### Analysis of binding pockets.

Finally, we sought to understand why KREDs P1-B12 and 404, which share a common evolutionary ancestor, give rise to opposite enantiomers of alcohol product **21** (Fig. 4b). Both enzymes were engineered from the WT short-chain aldo-KRED *Lactobacillus brevis*^55^. The WT has been studied previously and features two distinct, adjacent binding pockets in the active site of the enzyme^55,61^. The smaller substituent is thought to occupy the smaller, hydrophobic binding pocket, while the larger substituent occupies the larger, solvent exposed binding pocket. With the carbonyl’s orientation fixed via hydrogen bonding interactions and the NADPH cofactor bound behind the substrate (as depicted), the reduction occurs stereoselectively. In the case of acetophenone, the native substrate for the WT enzyme, the (*R*) enantiomer of phenethanol is obtained^55^.

The influence of the mutations present in KREDs P1-B12 and 404 can be understood based on the analysis and docking studies summarized in Fig. 4b. By analogy to the acetophenone-bound WT crystal structure^55^, P1-B12 and 404 were modeled with ketone substrate **14**. In the smaller binding pockets of P1-B12 and 404, residue 190 (Phe/Tyr in WT) has been replaced by a proline and glycine residue, respectively. This is thought to enlarge the small binding pockets relative to the WT KRED, thereby allowing larger ketone substituents, such as the aryl groups in **14**, to be accommodated in KREDs P1-B12 and 404. We attribute the differing sense of stereoinduction displayed by KREDs P1-B12 and 404 to additional variation in the small binding sites of the two engineered enzymes. Of the 19 mutations that differentiate P1-B12 from 404, a total of 8 line the substrate binding pocket and 5 of the mutations are in the small binding pockets, further influencing the size of the hydrophobic binding pocket. In the case of P1-B12, while the small binding pocket is larger compared to that of the WT, capable of accommodating aryl groups, it is still the smaller of the two binding pockets present in the enzyme, ultimately retaining the sense of stereoselectivity seen in the WT(i.e., R_S_ fits into the small binding pocket leading to the (*R*) major product). KRED 404, on the other hand, has additional small binding pocket mutations (smaller residues) relative to P1-B12 and the WT. In turn, this leads to a further enlargement of that binding site, which can consequently accommodate bulkier substituents. Thus, in the case of substrate **14**, the larger *o*-tolyl ketone substituent fits into the small binding pocket, leading to the (*S*) enantiomer of the corresponding alcohol **21** being the major product. The fact that both binding pockets are now relatively large in KRED 404 may explain why KRED 404 displays slightly lower stereoselectivity compared to P1-B12 (85% ee (*S*) vs 98% ee (*R*)). Nonetheless, it stands to reason that directed evolution could be used to further improve the stereoselectivity of KRED 404. This notion is supported by the ~10% increase in ee observed in the reduction of **14** when employing KRED 404 in place of KRED 402 (see Fig. 2b), which are differentiated by only four mutations (see Supplementary Tables 4 and 5). Taken together, these studies underscore the value of directed evolution as a powerful means of altering biocatalytic selectivities and engineering enzymes that display complementary senses of stereoinduction. This, in turn, is expected to foster the development of other one-pot, chemoenzymatic methodologies.

## Discussion

We have developed a chemoenzymatic methodology that enables the conversion of amides to enantioenriched alcohols. The transformation relies on two reactions that take place sequentially in one-pot, namely, the nickel-catalyzed Suzuki–Miyaura coupling of amides and the enzymatic reduction of diaryl ketone intermediates. Products are obtained in synthetically useful yields and ee’s up to 99%. Key to the success of these studies were the findings that the Suzuki–Miyaura coupling can take place in aqueous medium, that the engineered KRED P1-B12 can achieve the desired stereoselective reduction using an *i*-PrOH–NADPH cofactor recycling system, and that the two reactions could be performed sequentially in the same pot following reaction optimization. We also demonstrate that a different KRED, derived with the same WT ancestor, gives the opposite sense of stereochemical induction, which enabled formal syntheses of both enantiomers of the pharmaceutical orphenadrine. The stereoselectivities were understood on the basis of docking studies and an analysis of the enzyme binding pockets. These studies represent an enantioselective conversion of the amide to another functional group using catalytic C–N amide bond activation and provide an example of a chemoenzymatic methodology that forges both a C–C bond and an associated stereocenter. The success of this endeavor sets the stage for the development of other asymmetric transformations of amides employing chemoenzymatic strategies, as well as tandem variants of the reaction described herein.

## Methods

### Representative procedure for the one-pot, chemoenzymatic methodology.

A 2-dram vial containing a magnetic stir bar was flame-dried under reduced pressure and then allowed to cool under N_2_. The vial was charged with amide substrate (0.100 mmol, 1.00 equiv), boronic acid, pinacol ester (0.300 mmol, 3.00 equiv), and K_3_PO_4_ (0.200 mmol, 2.00 equiv). The vial was flushed with N_2_ and then taken into a glove box. In the glove box, the vial was charged with SIPr (0.0300 mmol, 30.0 mol%) and Ni(cod)_2_ (0.015 mmol, 15.0 mol%). Subsequently, the vial was removed from the glove box and degassed H_2_O (0.200 mL, 0.500 M) was added. The vial was then capped with a teflon-lined screw cap under a flow of N_2_, sealed with teflon tape, and stirred at 60 °C for 24 h.

The crude reaction mixture was removed from heat and cooled to 23 °C before being neutralized with 1.0 M HCl. To the reaction mixture was added *i*-PrOH (52.0 equiv) and the resultant mixture was stirred for 5 min before stopping stirring and allowing the layers to separate in the reaction vessel. Recycle Mix P (18.2 mg/mL) and KREDP1-B12 (10.0 mg/mL) were then added as a single solution in deionized H_2_O (4.00 mL). The reaction was then heated to 35 °C, placed under reduced pressure (350 mbar) and the mixture was stirred at a rate of 900 RPM for 24 h.

After 24 h, the vacuum was relieved and Na_2_NADP(+)•3H_2_O (0.044 equiv) was added. The reaction was again heated to 35 °C, placed under reduced pressure (350 mbar), and the mixture was stirred at a rate of 900 RPM for an additional 24 h.

The vacuum was relieved, the reaction was cooled to 23 °C, and the reaction mixture was transferred to a separatory funnel with deionized H_2_O (15 mL), brine (5.0 mL), and EtOAc (30 mL). The layers were separated and the aqueous layer was extracted with EtOAc (3 × 30 mL). The combined organic layers were dried over anhydrous Na_2_SO_4_, filtered, and the volatiles were removed under reduced pressure. The crude material was purified by preparative TLC.

### Synthetic procedures.

See Supplementary Methods, Supplementary Fig. 1, and Supplementary Tables 1–3.

### Bioinformatics and sequence alignment.

See Supplementary Methods and Supplementary Tables 4 and 5.

### Characterization.

See Supplementary Methods, Supplementary Figs. 2–41 for 1H and 13C NMR spectra, and Supplementary Figs. 42–78 for SFC traces of synthesized products.

## Data availability

The datasets generated during and/or analysed during the current study are available from the corresponding author on reasonable request.

## Supplementary Material

SI

## Figures and Tables

**Fig. 1:**
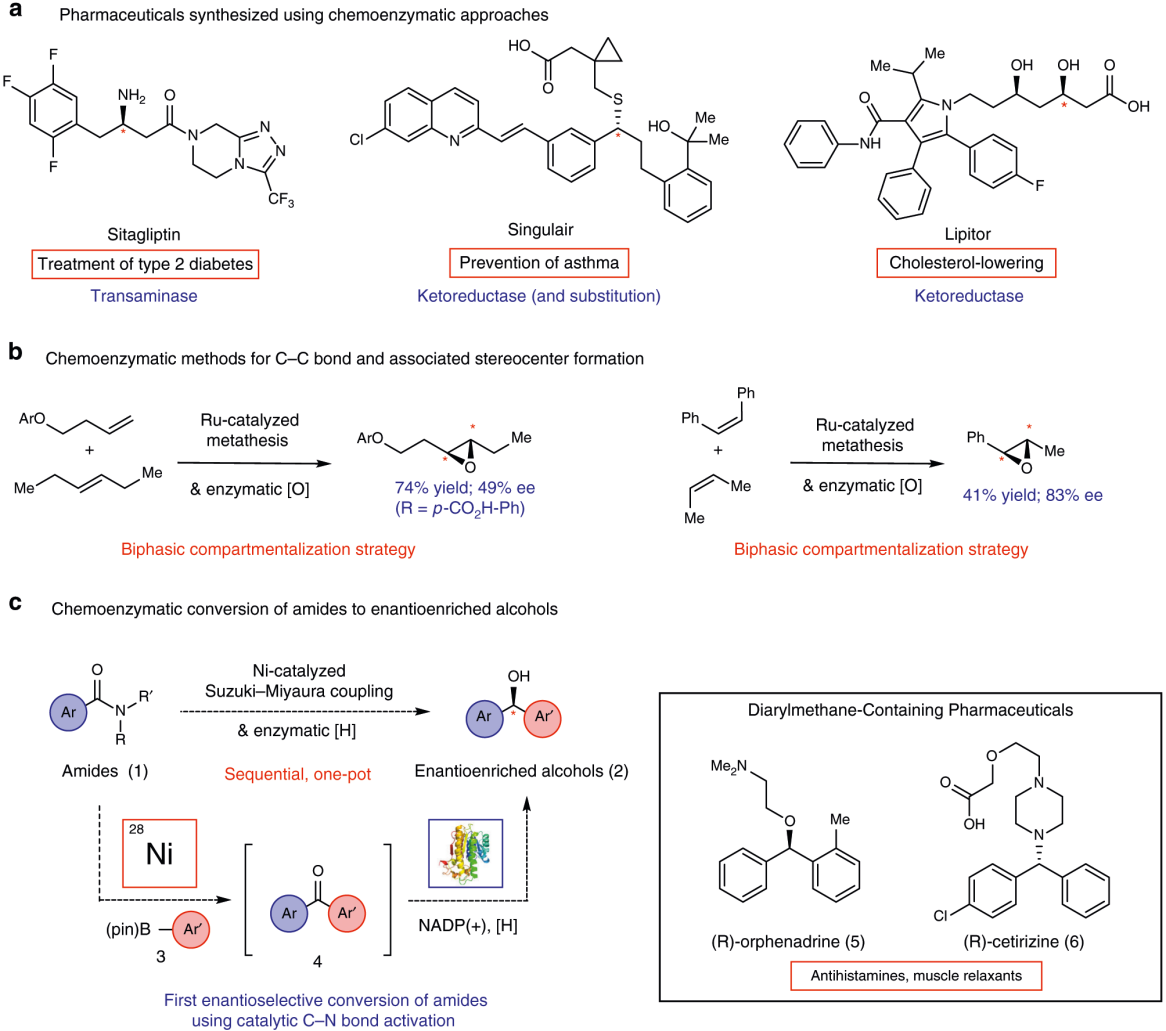
Landmarks in chemoenzymatic chemistry and the targeted chemoenzymatic transformation. **a** Pharmaceuticals accessed using chemoenzymatic synthesis. **b** Breakthroughs in chemoenzymatic methods development that rely on C–C bond and stereocenter formation at the reacting carbon atom(s), which provide streamlined retrosynthetic disconnections. **c** Current study involving the chemoenzymatic conversion of amides to enantioenriched diarylmethanols, a common motif in bioactive molecules and building block in stereoselective synthesis

## References

[1] Turner NJ & O’Reilly E Biocatalytic retrosynthesis. Nat. Chem. Biol 9, 285–288 (2013). 23594772 10.1038/nchembio.1235

[2] Bornscheuer UT Engineering the third wave of biocatalysis. Nature 485, 185–194 (2012). 22575958 10.1038/nature11117

[3] de Souza ROMA, Miranda LSM & Bornsheuer UT A retrosynthesis approach for biocatalysis in organic synthesis. Chem. Eur. J 23, 12040–12063 (2017). 28514518 10.1002/chem.201702235

[4] Hönig M, Sondermann P, Turner NJ & Carreira EM Enantioselective chemo- and biocatalysis: partners in retrosynthesis. Angew. Chem., Int. Ed 56, 8942–8973 (2017). 10.1002/anie.201612462 28407390

[5] Wallace S & Balskus EP Opportunities for merging chemical and biological synthesis. Curr. Opin. Biotechnol 30, 1–8 (2014). 24747284 10.1016/j.copbio.2014.03.006 PMC4199929

[6] Schoemaker HE, Mink D & Wubbolts MG Dispelling the myths – biocatalysis in industrial synthesis. Science 299, 1694–1697 (2003). 12637735 10.1126/science.1079237

[7] Simon RC, Mutti FG & Kroutil W Biocatalytic synthesis of enantiopure building blocks for pharmaceuticals. Drug Discov. Today Technol 10, e37–e44 (2013). 24050228 10.1016/j.ddtec.2012.08.002

[8] Hughes G & Lewis JC Introduction: biocatalysis in industry. Chem. Rev 118, 1–3 (2018). 29316793 10.1021/acs.chemrev.7b00741

[9] Sugai T, Higashibayashi S & Hanaya K Recent examples of the use of biocatalysts with high accessibility and availability in natural product synthesis. Tetrahedron 74, 3469–3487 (2018).

[10] Rudroff F Opportunities and challenges for combining chemo- and biocatalysis. Nat. Catal 1, 12–22 (2018).

[11] Denard CA, Hartwig JF & Zhao H Multistep one-pot reactions combining biocatalysis and chemical catalysts for asymmetric synthesis. ACS Catal. 3, 2856–2864 (2013).

[12] Gröger H & Hummel W Combining the ‘two worlds’ of chemocatalysis and biocatalysis towards multi-step one-pot processes in aqueous media. Curr. Opin. Chem. Biol 19, 171–179 (2014). 24709123 10.1016/j.cbpa.2014.03.002

[13] Ríos-Lombardía N, García-Álvarez J & González-Sabín J One-pot combination of metal- and biocatalysis in water for the synthesis of chiral molecules. Catalysts 8, 75 (2018).

[14] Sirasani G, Tong L & Balskus EP A biocompatible alkene hydrogenation merges organic synthesis with microbial metabolism. Angew. Chem., Int. Ed 53, 7785–7788 (2014). 10.1002/anie.201403148 PMC4127630 24916924

[15] Wallace S & Balskus EP Interfacing microbial styrene production with a biocompatible cyclopropanation reaction. Angew. Chem., Int. Ed 54, 7106–7109 (2015). 10.1002/anie.201502185 PMC4494747 25925138

[16] Dockrey SAB, Lukowski AL, Becker MR & Narayan ARH Biocatalytic site- and enantioselective oxidative dearomatization of phenols. Nat. Chem 10, 119–125 (2018). 29359749 10.1038/nchem.2879 PMC6503525

[17] Ro D-K Production of the antimalarial drug precursor artemisinic acid in engineered yeast. Nature 440, 940–943 (2006). 16612385 10.1038/nature04640

[18] Trost BM Total synthesis of (–)-lasonolide A. J. Am. Chem. Soc 138, 11690–11701 (2016). 27548113 10.1021/jacs.6b05127 PMC5728428

[19] Loskot SA, Romney DK, Arnold FH & Stoltz BM Enantioselective total synthesis of nigelladine A via late-stage C–H oxidation enabled by an engineered P450 enzyme. J. Am. Chem. Soc 139, 10196–10199 (2017). 28721734 10.1021/jacs.7b05196 PMC5679227

[20] Corey EJ & Cheng X-M “The Basis for Retrosynthetic Analysis” in The Logic of Chemical Synthesis (pp. 1–16. Wiley, New York, NY, 1995).

[21] Machajewski TD & Wong C-H The catalytic asymmetric aldol reaction. Angew. Chem., Int. Ed 39, 1352–1374 (2000). 10.1002/(sici)1521-3773(20000417)39:8<1352::aid-anie1352>3.0.co;2-j 10777624

[22] Palomo C, Oiarbide M & García JM Current progress in the asymmetric aldol addition reaction. Chem. Soc. Rev 33, 65–75 (2004). 14767502 10.1039/b202901d

[23] Cordova A (ed) Catalytic Asymmetric Conjugate Reactions. (Wiley-VCH, Weinheim, 2010).

[24] Hawner C & Alexakis A Metal-catalyzed asymmetric conjugate addition reaction: formation of quaternary stereocenters. Chem. Commun 46, 7295–7306 (2010). 10.1039/c0cc02309d 20734008

[25] Dean SM, Greenberg WA & Wong C-H Recent advances in aldolasecatalyzed asymmetric synthesis. Adv. Synth. Catal 349, 1308–1320 (2007).

[26] Clapés P, Wolf-Dieter F, Sprenger GA & Samland AK Recent progress in stereoselective synthesis with aldolases. Curr. Opin. Chem. Biol 14, 154–167 (2010). 20071212 10.1016/j.cbpa.2009.11.029

[27] Fischbach MA & Walsh CT Assembly-line enzymology for polyketide and nonribosomal peptide antibiotics: logic, machinery, and mechanisms. Chem. Rev 106, 3468–3496 (2006). 16895337 10.1021/cr0503097

[28] Denard CA Cooperative tandem catalysis by an organometallic complex and a metalloenzyme. Angew. Chem., Int. Ed 53, 465–469 (2014). 10.1002/anie.201305778 24536102

[29] Denard CA Development of a one-pot tandem reaction combining ruthenium-catalyzed alkene metathesis and enantioselective enzymatic oxidation to produce aryl epoxides. ACS Catal. 5, 3817–3822 (2015).

[30] Wang Y, Bartlett MJ, Denard CA, Hartwig JF & Zhao H Combining Rh-catalyzed diazocoupling and enzymatic reduction to efficiently synthesize enantioenriched 2-substituted succinate derivatives. ACS Catal. 7, 2548–2552 (2017).

[31] Weires NA, Baker EL & Garg NK Nickel-catalysed Suzuki–Miyaura coupling of amides. Nat. Chem 8, 75–79 (2016). 26673267 10.1038/nchem.2388

[32] Boit TB, Weires NA, Kim J & Garg NK Nickel-catalyzed Suzuki–Miyaura coupling of aliphatic amides. ACS Catal. 8, 1003–1008 (2018). 29682398 10.1021/acscatal.7b03688 PMC5906067

[33] Shi S, Meng G & Szostak M Synthesis of biaryls through nickel-catalyzed Suzuki–Miyaura coupling of amides by carbon–nitrogen bond cleavage. Angew. Chem., Int. Ed 55, 6959–6963 (2016). 10.1002/anie.201601914 27101428

[34] Huisman GW, Liang J & Krebber A Practical chiral alcohol manufacture using ketoreductases. Curr. Opin. Chem. Biol 14, 122–129 (2010). 20071211 10.1016/j.cbpa.2009.12.003

[35] Moore JC, Pollard DJ, Kosjek B & Devine PN Advances in the enzymatic reduction of ketones. Acc. Chem. Res 40, 1412–1419 (2007). 18052114 10.1021/ar700167a

[36] Greenberg A, Breneman CM, Liebman JF (eds) The Amide Linkage: Structural Significance in Chemistry, Biochemistry, and Materials Science. (Wiley, New York, NY, 2002).

[37] Dander JE & Garg NK Breaking amides using nickel catalysis. ACS Catal. 7, 1413–1423 (2017). 28626599 10.1021/acscatal.6b03277 PMC5473294

[38] Meng G, Shi S & Szostak M Cross-coupling of amides by N–C bond activation. Synlett 27, 2530–2540 (2016).

[39] Takise R, Muto K & Yamaguchi J Cross-coupling of aromatic esters and amides. Chem. Soc. Rev 46, 5864–5888 (2017). 28685781 10.1039/c7cs00182g

[40] Tasker SZ, Standley EA & Jamison TF Recent advances in homogeneous nickel catalysis. Nature 509, 299–309 (2014). 24828188 10.1038/nature13274 PMC4344729

[41] Ananikov VP Nickel: the “spirited horse” of transition metal catalysis. ACS Catal. 5, 1964–1971 (2015).

[42] Lennox AJJ & Lloyd-Jones GC Selection of boron reagents for Suzuki–Miyaura coupling. Chem. Soc. Rev 43, 412–443 (2014). 24091429 10.1039/c3cs60197h

[43] Miyaura N & Suzuki A Palladium-catalyzed cross-coupling reactions of organoboron compounds. Chem. Rev 95, 2457–2483 (1995).

[44] Truppo MD, Pollard D & Devine P Enzyme-catalyzed enantioselective diaryl ketone reductions. Org. Lett 9, 335–338 (2007). 17217298 10.1021/ol0627909

[45] De Wildeman SMA, Sonke T, Schoemaker HE & May O Biocatalytic reductions: from lab curiosity to “first choice”. Acc. Chem. Res 40, 1260–1266 (2007). 17941701 10.1021/ar7001073

[46] Schmidt F, Stemmler RT, Rudolph J & Bolm C Catalytic asymmetric approaches towards enantiomerically enriched diarylmethanols and diarylmethylamines. Chem. Soc. Rev 35, 454–470 (2006). 16636728 10.1039/b600091f

[47] Medina JM, Moreno J, Racine S, Du S & Garg NK Mizoroki–Heck cyclizations of amide derivatives for the introduction of quaternary centers. Angew. Chem., Int. Ed 56, 6567–6571 (2017). 10.1002/anie.201703174 PMC5669036 28467029

[48] Nagao Y, Hisanaga T, Egami H, Kawato Y & Hamashima Y Desymmetrization of bisallylic amides through catalytic enantioselective bromocyclization with BINAP monoxide. Chem. Eur. J 23, 16758–16762 (2017). 29044749 10.1002/chem.201704847

[49] Quasdorf KW, Tian X & Garg NK Suzuki–Miyaura coupling of aryl carbamates. J. Am. Chem. Soc 130, 14422–14423 (2008).18839946

[50] Quasdorf KW Suzuki–Miyaura cross-coupling of aryl carbamates and sulfamates: experimental and computational studies. J. Am. Chem. Soc 133, 6352–6363 (2011). 21456551 10.1021/ja200398c PMC3091075

[51] Inada K & Miyaura N Synthesis of biaryls via cross-coupling reaction of arylboronic acids with aryl chlorides catalyzed by NiCl_2_/triphenylphosphine complexes. Tetrahedron 56, 8657–8660 (2000).

[52] Guan B-T, Wang Y, Li B-J, Yu D-G & Shi Z-J Biaryl construction via Ni-catalyzed C–O activation of phenolic carboxylates. J. Am. Chem. Soc 130, 14468–14470 (2008). 18847272 10.1021/ja8056503

[53] Handa S, Skack ED & Lipshutz BH Nanonickel-catalyzed Suzuki–Miyaura cross-couplings in water. Angew. Chem., Int. Ed 54, 11994–11998 (2015). 10.1002/anie.201505136 PMC4677689 26305994

[54] Romney DK, Arnold FH, Lipshutz BH & Li C-J Chemistry takes a bath: reactions in aqueous media. J. Org. Chem 83, 7319–7322 (2018). 30025465 10.1021/acs.joc.8b01412

[55] Schlieben NH Atomic resolution structures of (*R*)-specific alcohol dehydrogenase from Lactobacillus brevis provide the structural bases of its substrate and cosubstrate specificity. J. Mol. Biol 349, 801–813 (2005). 15896805 10.1016/j.jmb.2005.04.029

[56] Alvizo O, Collier SJ, Hennemann H-GJ, Oh SH & Zha W Ketoreductase polypeptides for the preparation of phenylephrine. US Patent 9,834,758. 5 12 2017.

[57] Savile C, Gruber JM, Mundorff E, Huisman G & Collier SJ Ketoreductase polypeptides for the production of 3-aryl-3-hydroxypropanamine from a 3-aryl-3-ketopropanamine. US Patent 8288141. 10 10 2016.

[58] Tobisu M & Chatani N Cross-couplings using aryl ethers via C–O bond activation enabled by nickel catalysts. Acc. Chem. Res 48, 1717–1726 (2015). 26036674 10.1021/acs.accounts.5b00051

[59] Sui Y-Z Cu(II)-catalyzed asymmetric hydrosilylation of diaryl- and aryl heteroaryl ketones: application in the enantioselective synthesis of orphenadrine and neobenodine. Chem. Eur. J 18, 7486–7492 (2012). 22573364 10.1002/chem.201200379

[60] Kumar R, Hoshimoto Y, Yabuki H, Ohashi M & Ogoshi S Nickel(0)-catalyzed enantio- and diastereoselective synthesis of benzoxasiloles: ligand-controlled switching from inter- to intramolecular aryl-transfer process. J. Am. Chem. Soc 137, 11838–11845 (2015). 26301429 10.1021/jacs.5b07827

[61] Noey EL Origins of stereoselectivity in evolved ketoreductases. Proc. Natl. Acad. Sci. USA 112, E7065–7072 (2016). 10.1073/pnas.1507910112 PMC4697376 26644568

