# Supplementary material for: Chemoenzymatic conversion of amides to enantioenriched alcohols in aqueous medium
Source: Commun Chem. Author manuscript; Available in PMC 2020 Feb 10. (PMC7010078; doi:10.1038/s42004-019-0182-8)
Supplement: SI [file NIHMS1068772-supplement-SI.pdf]

Chemoenzymatic conversion of amides to enantioenriched alcohols in aqueous medium

Jacob E. Dander, Maude Giroud, Sophie Racine, Evan R. Darzi, Oscar Alvizo, David Entwistle, and Neil K. Garg*

Department of Chemistry and Biochemistry, University of California, Los Angeles, California 90095

Supplementary Methods 2

Part I: Experimental Section 2
Materials and Methods 2
In-Hood Vacuum Pump Set-Up and Specifications 3
Experimental Procedures 4
A. Syntheses of Amide Substrates 4
B. Suzuki–Miyaura Reaction Optimization Experiments 6
C. Scope of the Suzuki–Miyaura Coupling of Amides in Aqueous Media 8
D. Evaluation of KREDs 14
E. One-Pot Reaction Optimization Experiments 16
F. Scope of the One-Pot Reaction 19
G. Verification of Enantiopurity 27
i. Preparation of Racemic Alcohols 27
ii. Enantioselective Diarylmethanol Synthesis by Asymmetric Arylation 29

Part II: Bioinformatics Section 32
Sequence Alignments 32

Supplementary References 33

^{1}H NMR Spectra 35
^{13}C NMR Spectra 69
SFC Traces 75

Supplementary Methods

Part I: Experimental Section

Materials and Methods Unless stated otherwise, reactions were conducted in flame-dried glassware under an atmosphere of nitrogen and commercially obtained reagents were used as received. Anhydrous solvents were either freshly distilled or passed through activated alumina columns, unless otherwise stated. Non-commercially available reagents and substrates were synthesized following protocols specified in Section A, Section C, and Section G in the Experimental Procedures. Non-commercially available substrates were synthesized following protocols specified in Section A in the Experimental Procedures. Prior to use, toluene was purified by distillation and taken through five freeze-pump-thaw cycles. Deionized water used as a reaction solvent was purified using a Q-POD® Ultrapure Water Remote Dispenser from Millipore equipped with a Millipak Express 40 Q-POD (0.22 μm filter), and it was sparged with N_2 for a minimum of 30 min prior use. Isopropyl alcohol HPLC grade was obtained from Millipore Sigma. Benzylamine was obtained from Sigma–Aldrich. Boronate esters **SI-9**, **SI-11**, **SI-12**, and **SI-16** were obtained from Combi-Blocks, and boronate esters **23**, **SI-10**, **SI-13**, **SI-14**, and **SI-15** were obtained from Sigma–Aldrich. Carboxylic acid **SI-8**, *N*,*N*-dimethylpyridin-4-amine (DMAP), 1,3,5-trimethoxybenzaldehyde (**SI-22**), and benzaldehyde (**SI-24**) were obtained from Sigma–Aldrich. $Ni(cod)_2$ and 1,3-bis(2,6-diisopropylphenyl)-4,5-dihydroimidazol-2-ylidine (SIPr) were obtained from Strem Chemicals. K_3PO_4 was obtained from Acros. 1,3,5-Trimethoxybenzene (TMB), *rac*-2-methylbenzhydrol (**21**), and *rac*-4-methylbenzhydrol (**22**) were obtained from Alfa Aesar. 3-(Ethyliminomethyleneamino)-*N*,*N*-dimethylpropan-1-amine (EDC) and di-*tert*-butyl dicarbonate (Boc_2O) were obtained from Oakwood. Benzotriazol-1-ol (HOBt) was obtained from Chemimpex. Ketoreductases (KREDs) 401–404, P1-B12, and Recycle Mix P were obtained from Codexis. Benzophenones **14** and **16** were obtained from TCI. Phenyl boronic acid (**SI-23**) was obtained from Oakwood, and *N*-methyl-5-indole boronic acid (**SI-25**) was obtained from Combi-Blocks. Diethylzinc was obtained from Sigma–Aldrich as a 1.1 M solution in toluene. Phenyl magnesium bromide (**SI-21**) was obtained from Sigma–Aldrich as a 1.0 M solution in THF. $Na_2NADP(+)\cdot 3H_2O$ was obtained from VWR. (*S*)-Methyl-1-tritylaziridine-2-carboxylate (**SI-20**) was obtained from Combi-Blocks. Reaction temperatures were controlled using an IKAmag

temperature modulator, and unless stated otherwise, performed at room temperature (approximately 23 °C). Thin-layer chromatography (TLC) was conducted with EMD gel 60 F_{254} pre-coated plates (0.25 mm for analytical chromatography and 0.50 mm for preparative chromatography) and visualized using a combination of UV and potassium permanganate staining techniques. Silicycle Siliaflash P60 (particle size 40–63 μm) was used for flash column chromatography. ^{1}H NMR spectra were recorded on Bruker spectrometers (at 300, 400, and 500 MHz) and are reported relative to residual solvent signals. Data for ^{1}H NMR spectra are reported as follows: chemical shift (δ ppm), multiplicity, coupling constant (Hz), integration. Data for ^{13}C NMR are reported in terms of chemical shift (125 MHz). For NMR spectra see Supplementary Figures 2–41. IR spectra were recorded on a Perkin-Elmer UATR Two FT-IR spectrometer and are reported in terms of frequency absorption (cm^{-1}). DART-MS spectra were collected on a Thermo Exactive Plus MSD (Thermo Scientific) equipped with an ID-CUBE ion source and a Vapur Interface (IonSense Inc.). Both the source and MSD were controlled by Excalibur software version 3.0. The analyte was spotted onto OpenSpot sampling cards (IonSense Inc.) using $CHCl_3$ as the solvent. Ionization was accomplished using UHP He plasma with no additional ionization agents. The mass calibration was carried out using Pierce LTQ Velos ESI (+) and (–) Ion calibration solutions (Thermo Fisher Scientific). Uncorrected melting points were measured using a digimelt MPA160 melting point apparatus. Optical rotations were measured with a Rudolf Autopol III Automatic Polarimeter. Determination of enantiopurity was carried out on a Mettler Toledo SFC (supercritical fluid chromatography) using Daicel ChiralPak AD–3, Daicel ChiralPak AD–H, Daicel ChiralPak AS–H, Daicel ChiralPak IA–3, Daicel ChiralPak IB–H, Daicel ChiralPak IC–3, and Daicel ChiralPak OJ–H columns. Data for SFC spectra are reported in enantiomeric excess (ee). For SFC chromatograms see Supplementary Figures 42–78.

In-Hood Vacuum Pump Set-Up and Specifications

Some protocols described in Sections E and F of the Experimental Procedures require that reactions be conducted under reduced pressure. For these procedures, a vacuum was applied to reaction mixtures using a Büchi V-700 vacuum pump equipped with a Büchi V-850 vacuum controller set to 350 mbar (Supplementary Figure 1). An in-line 125 mL Woulff bottle and glass three-neck round bottom flask (1000 mL) were sequentially connected to the vacuum pump via

VWR brand "Select Grade" PVC tubing (1/4" i.d. x 1/2" o.d. x 1/8" wall thickness) and aided in stabilizing the applied vacuum. The PVC tubing was connected to the 2-dram reaction vials via the combined use of 24/40-hose barb adapters and 24/40 rotary evaporator adapters equipped with 15-425 connectors; joints were sealed with Teflon and/or electrical tape. A Granville-Phillips 475 Convectron® Vacuum Gauge Controller was used to assess the applied vacuum (347–373 mbar range of pressures observed over 1 min).

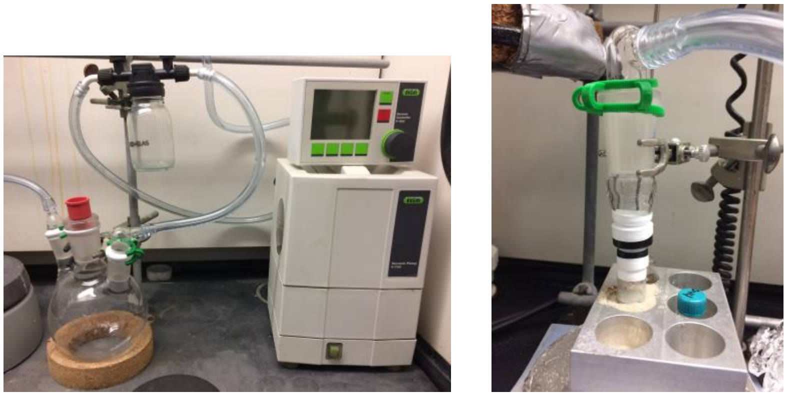

Supplementary Figure 1. In-hood vacuum pump set-up.

Experimental Procedures

A. Syntheses of Amide Substrates

Note: Supplementary information for the syntheses of amides **23**,[1] **SI-1**[2] **SI-2**,[2] **SI-3**,[1] **SI-4**,[2] **SI-5**,[3] and **SI-6**[2] has been published and spectral data match those previously reported.

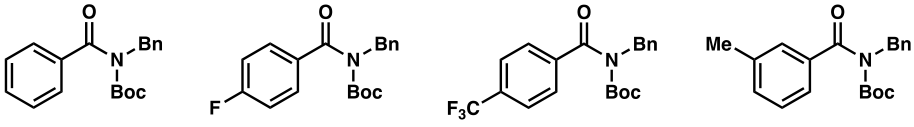

23 ***SI-1*** ***SI-2*** ***SI-3***

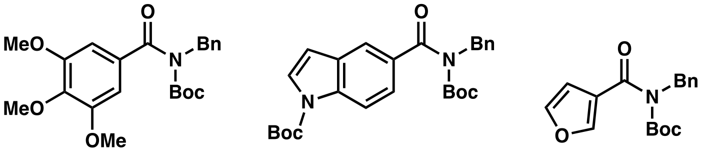

SI-4 ***SI-5*** ***SI-6***

Synthesis of the remaining substrate, **SI-7**, is as follows:

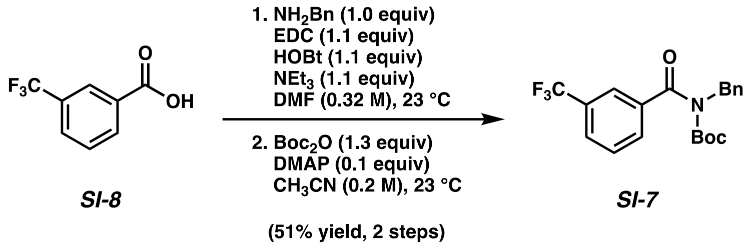

Amide SI-7: To a solution of carboxylic acid **SI-8** (3.0 g, 16.0 mmol, 1.0 equiv) in DMF (50 mL, 0.32 M) was added EDC (3.3 g, 17.0 mmol, 1.1 equiv), HOBt (2.7 g, 17.0 mmol, 1.1 equiv), triethylamine (2.4 mL, 17.0 mmol, 1.1 equiv), and benzylamine (1.9 mL, 17.0 mmol, 1.1 equiv). The resulting mixture was stirred at 23 °C for 15 h, and then diluted with deionized water (100 mL). The layers were separated and the aqueous layer was extracted with EtOAc (3 x 100 mL). The combined organic layers were then washed successively with deionized water (2 x 100 mL) and brine (100 mL) before being dried over Na_2SO_4 and subsequently filtered. The volatiles were removed under reduced pressure. The resulting crude solid material was used in the subsequent step without further purification.

To a flask containing the crude material from the previous step was added acetonitrile (80 mL, 0.2 M) and the resulting mixture was stirred. DMAP (190 mg, 1.60 mmol, 0.1 equiv) and Boc_2O (4.5 g, 20.0 mmol, 1.3 equiv) were then added to the resultant solution and the reaction vessel was flushed with N_2 for 5 min. The reaction mixture was then stirred at 23 °C for 38 h. The volatiles were removed under reduced pressure and the resulting crude residue was purified by flash chromatography (9:1 hexanes:EtOAc). Subsequently, the purified material was recrystallized from heptanes to yield amide **SI-7** (3.03 g, 51% yield, over two steps) as a white solid. Amide **SI-7**: m.p. 57–60 °C; R_f 0.32 (4:1 hexanes:EtOAc); ^{1}H NMR (500 MHz, $CDCl_3$): δ 7.75 (br s, 1H), 7.72 (br d, *J* = 7.87, 1H), 7.69 (br d, *J* = 7.69, 1H), 7.53 (t, *J* = 7.69, 1H), 7.43–7.41 (m, 2H), 7.34 (t, *J* = 7.69, 2H), 7.28 (tt, *J* = 7.37, 2.24, 1H), 5.00 (s, 2H), 1.14 (s, 9H); ^{13}C NMR (125 MHz, $CDCl_3$): δ 171.7, 153.1, 138.6, 137.6, 130.7, 128.9, 128.7, 128.3, 127.7, 127.6 (q, $^3J_{CF}$ = 3.79), 124.4 (q, $^3J_{CF}$ = 8.85), 123.7 (d, $^1J_{CF}$ = 272.51), 84.0, 49.1, 27.5; ^{19}F{^{1}H} NMR (376 MHz, $CDCl_3$): δ –62.81; IR (film): 2981, 1733, 1675, 1615, 1359, 1335, 1319, 1222, 1124, 1071, 978, 849, 747, 699, 652 cm^{-1}; HRMS-APCI (*m/z*) $[M + H]^+$ calcd for $C_{20}H_{21}NO_3F_3^+$, 380.1468; found 380.1450.

B. Suzuki–Miyaura Reaction Optimization Experiments

Representative Procedure A for reaction optimization. Ketone 14 (Supplementary Table 1, entry 1):

A 1-dram vial containing a magnetic stir bar was flame-dried under reduced pressure and then allowed to cool under N_2. The vial was charged with amide substrate **23** (31.1 mg, 0.1 mmol, 1.0 equiv), 2-methylphenylboronic acid, pinacol ester (**24**, 65.4 mg, 0.3 mmol, 3.0 equiv), and K_3PO_4 (42.4 mg, 0.2 mmol, 2.0 equiv). The vial was flushed with N_2 and then taken into a glove box. In the glove box, the vial was charged with SIPr (3.91 mg, 0.01 mmol, 10 mol%), Ni(cod)$_2$ (2.75 mg, 0.01 mmol, 10 mol%), and PhMe (0.1 mL, 1.0 M). Subsequently, the vial was removed from the glove box and degassed H_2O (3.60 μL, 0.2 mmol, 2.0 equiv) was added. The vial was then capped with a Teflon-lined screw cap under a flow of N_2, sealed with Teflon tape, and stirred at 50 °C for 24 h. After cooling to 23 °C, the mixture was diluted with hexanes (0.5 mL) and filtered over a plug of silica gel (10 mL of EtOAc eluent). The volatiles were removed under reduced pressure and the yield was determined by ^{1}H NMR analysis with 1,3,5-trimethoxybenzene as an external standard. The spectral data match those previously reported in the literature.[4]

Representative Procedure B for reaction optimization. Ketone 14 (Supplementary Table 1, entry 7):

A 1-dram vial containing a magnetic stir bar was flame-dried under reduced pressure and then allowed to cool under N_2. The vial was charged with amide substrate **23** (31.1 mg, 0.1 mmol, 1.0 equiv), 2-methylphenylboronic acid, pinacol ester (**24**, 65.4 mg, 0.3 mmol, 3.0 equiv), and K_3PO_4 (42.4 mg, 0.2 mmol, 2.0 equiv). The vial was flushed with N_2 and then taken into a glove box. In the glove box, the vial was charged with SIPr (11.7 mg, 0.03 mmol, 30 mol%) and Ni(cod)$_2$ (4.2 mg, 0.015 mmol, 15 mol%). Subsequently, the vial was removed from the glove box and degassed H_2O (0.2 mL, 0.5 M) was added. The vial was then capped with a Teflon-lined screw cap under a flow of N_2, sealed with Teflon tape, and stirred at 60 °C for 24 h. After cooling to 23 °C, the mixture was diluted with EtOAc (1.0 mL) and the layers were separated. The aqueous layer was extracted with EtOAc (3 x 1 mL) and the combined organic layers were dried via passage through a plug of anhydrous $MgSO_4$ (10 mL of EtOAc eluent). The volatiles were removed under reduced

pressure and the yield was determined by ^{1}H NMR analysis with 1,3,5-trimethoxybenzene as an external standard. The spectral data match those previously reported in the literature.[4]

Optimization efforts that deviate from the above conditions are indicated below.

Supplementary Table 1. Optimization of Reaction Conditions.[a]

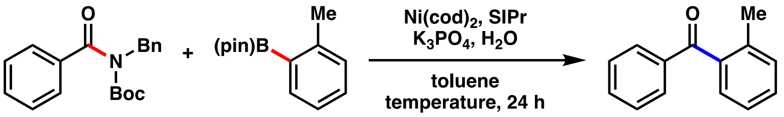

23 **24** **14**

Entry	*Rep. Procedure*	*Toluene*	*H_2O*	*$Ni(cod)_2$ (mol%)*	*SIPr (mol%)*	*Temp. (° C)*	*Yield*
1	A	1.0 M	2.0 equiv	10	10	50	87%
2	A	–	2.0 equiv	10	10	50	81%
3	B	–	1.0 M	10	10	50	39%
4	B	–	0.5 M	10	10	50	73%
5	B	–	0.5 M	10	10	60	77%
6	B	–	0.5 M	10	20	60	85%
7	B	–	0.5 M	15	30	60	91%

[a] Yields were determined using ^{1}H NMR analysis with 1,3,5-trimethoxybenzene (TMB) as an external standard.

C. Scope of the Suzuki–Miyaura Coupling of Amides in Aqueous Media

Representative Procedure A for the Suzuki–Miyaura coupling (Figure 2, synthesis of ketone 7 used as an example).

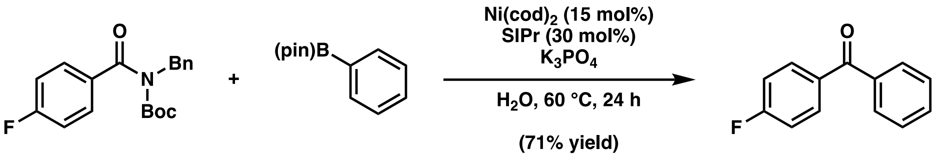

SI-1 *SI-9* *7*

Ketone 7: A 1-dram vial containing a magnetic stir bar was flame-dried under reduced pressure and then allowed to cool under N_2. The vial was charged with amide substrate **SI-1** (32.9 mg, 0.1 mmol, 1.0 equiv), phenylboronic acid, pinacol ester (**SI-9**, 61.0 mg, 0.3 mmol, 3.0 equiv), and K_3PO_4 (42.4 mg, 0.2 mmol, 2.0 equiv). The vial was flushed with N_2 and then taken into a glove box. In the glove box, the vial was charged with SIPr (11.7 mg, 0.03 mmol, 30 mol%) and Ni(cod)$_2$ (4.2 mg, 0.015 mmol, 15 mol%). Subsequently, the vial was removed from the glove box and degassed H_2O (0.2 mL, 0.5 M) was added. The vial was then capped with a Teflon-lined screw cap under a flow of N_2, sealed with Teflon tape, and stirred at 60 °C for 24 h. After cooling to 23 °C, the mixture was diluted with EtOAc (1.0 mL) and the layers were separated. The aqueous layer was extracted with EtOAc (3 x 1 mL) and the combined organic layers were dried by passage through a plug of anhydrous $MgSO_4$ (10 mL of EtOAc eluent), before the volatiles were removed under reduced pressure.

The crude material was purified by sequential preparative TLC (3:1:1 hexanes:CH_2Cl_2:Et_2O; 10:1 hexanes:EtOAc) to generate ketone **7** (71% yield, average of two experiments) as a colorless oil. Ketone **7**: R_f 0.40 (9:1 hexanes:EtOAc). The spectral data match those previously reported in the literature.[5]

Representative Procedure B for the Suzuki–Miyaura coupling (Figure 2, synthesis of ketone 8 used as an example):

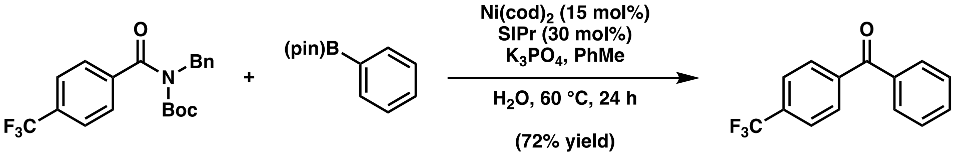

SI-2 *SI-9* *8*

Ketone 8. A 1-dram vial containing a magnetic stir bar was flame-dried under reduced pressure and then allowed to cool under N_2. The vial was charged with amide substrate **SI-2** (38.0 mg, 0.1 mmol, 1.0 equiv), phenylboronic acid, pinacol ester (**SI-9**, 61.2 mg, 0.3 mmol, 3.0 equiv), and K_3PO_4 (42.4 mg, 0.2 mmol, 2.0 equiv). The vial was flushed with N_2 and then taken into a glove box. In the glove box, the vial was charged with SIPr (11.7 mg, 0.03 mmol, 30 mol%), Ni(cod)$_2$ (4.2 mg, 0.015 mmol, 15 mol%), and PhMe (50 μL). Subsequently, the vial was removed from the glove box and degassed H_2O (0.2 mL, 0.5 M) was added. The vial was then capped with a Teflon-lined screw cap under a flow of N_2, sealed with Teflon tape, and stirred at 60 °C for 24 h. After cooling to 23 °C, the mixture was diluted with EtOAc (1.0 mL) and the layers were separated. The aqueous layer was extracted with EtOAc (3 x 1 mL) and the combined organic layers were dried by passage through a plug of anhydrous $MgSO_4$ (10 mL of EtOAc eluent), before the volatiles were removed under reduced pressure.

The crude material was purified by sequential preparative TLC (9:1 hexanes:EtOAc, eluted twice; 9:1 hexanes:EtOAc, eluted twice) to generate ketone **8** (72% yield, average of two experiments) as a white solid. Ketone **8**: R_f 0.57 (9:1 hexanes:EtOAc). The spectral data match those previously reported in the literature.[6]

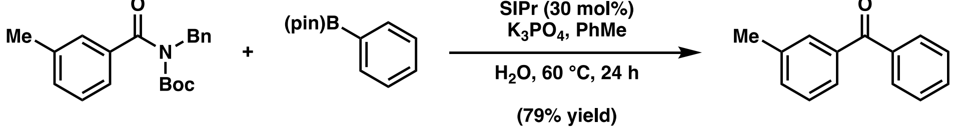

SI-3 *SI-9* *9*

Ketone 9. Followed representative procedure B. Purification by sequential preparative TLC (9:1 hexanes/EtOAc, eluted twice; 5:1:1 hexanes:CH_2Cl_2:Et_2O) generated ketone **9** (79% yield, average

of two experiments) as a colorless oil. Ketone **9**: R_f 0.46 (9:1 hexanes:EtOAc). The spectral data match those previously reported in the literature.[4]

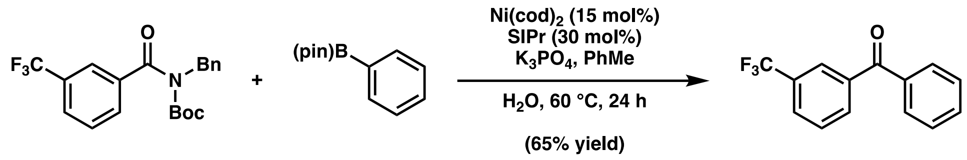

SI-7 *SI-9* *10*

Ketone 10. Followed representative procedure B. Purification by sequential preparative TLC (9:1 hexanes:EtOAc; 100% benzene, eluted twice) generated ketone **10** (65% yield, average of two experiments) as a colorless oil. Ketone **10**: R_f 0.48 (9:1 hexanes:EtOAc). The spectral data match those previously reported in the literature.[7]

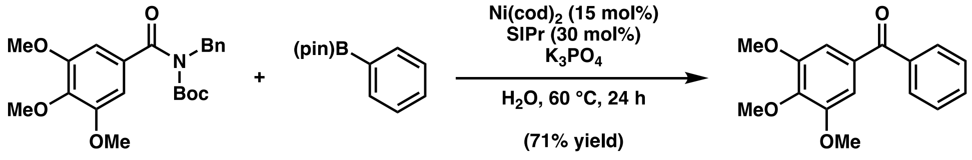

SI-4 *SI-9* *11*

Ketone 11. Followed representative procedure A. Purification by sequential preparative TLC (4:1 hexanes:EtOAc; 30:40:1 hexanes:benzene:MeCN) generated ketone **11** (71% yield, average of two experiments) as a white solid. Ketone **11**: R_f 0.12 (10:1 hexanes:EtOAc). The spectral data match those previously reported in the literature.[8]

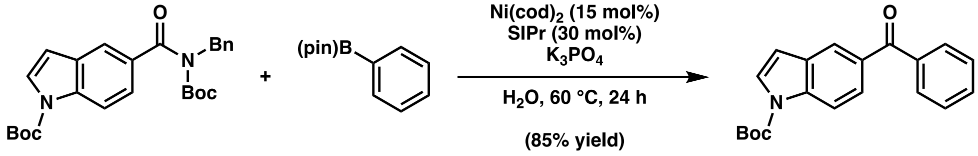

SI-5 *SI-9* *12*

Ketone 12. Followed representative procedure A. Purification by preparative TLC (10:1 hexanes:EtOAc) generated ketone **12** (85% yield, average of two experiments) as a colorless oil. Ketone **12**: R_f 0.38 (10:1 hexanes:EtOAc). The spectral data match those previously reported in the literature.[9]

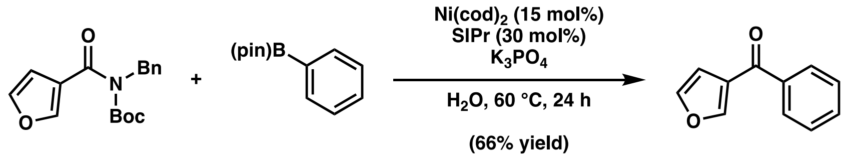

SI-6 *SI-9* *13*

Ketone 13. Followed representative procedure A. Purification by sequential preparative TLC (10:1 hexanes:EtOAc; 100% benzene) generated ketone **13** (66% yield, average of two experiments) as a colorless oil. Ketone **13**: R*f* 0.38 (10:1 hexanes:EtOAc). The spectral data match those previously reported in the literature.[10]

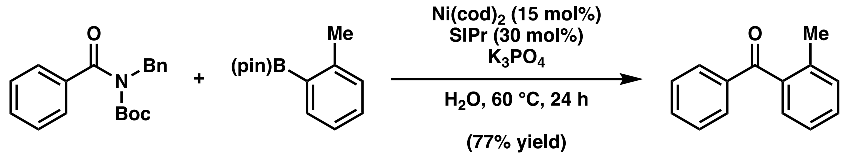

23 *24* *14*

Ketone 14. Followed representative procedure A. Purification by preparative TLC (9:1 hexanes:EtOAc) generated ketone **14** (77% yield, average of two experiments) as a colorless oil. Ketone **14**: R*f* 0.54 (9:1 hexanes:EtOAc). The spectral data match those previously reported in the literature.[4]

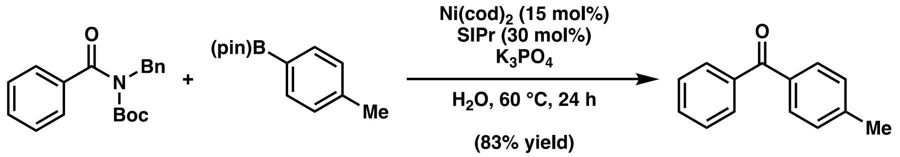

23 *SI-10* *15*

Ketone 15. A 1-dram vial containing a magnetic stir bar was flame-dried under reduced pressure and then allowed to cool under N_2. The vial was charged with amide substrate **23** (31.1 mg, 0.1 mmol, 1.0 equiv), 4-methylphenylboronic acid, pinacol ester (**SI-10**, 65.4 mg, 0.3 mmol, 3.0 equiv), and K_3PO_4 (42.4 mg, 0.2 mmol, 2.0 equiv). The vial was flushed with N_2 and then taken into a glove box. In the glove box, the vial was charged with SIPr (11.7 mg, 0.03 mmol, 30 mol%) and Ni(cod)$_2$ (4.2 mg, 0.015 mmol, 15 mol%). Subsequently, the vial was removed from the glove box and degassed H_2O (0.2 mL, 0.5 M) was added. The vial was then capped with a Teflon-lined screw cap under a flow of N_2, sealed with Teflon tape, and stirred at 60 °C for 24 h. After cooling

to 23 °C, the mixture was diluted with EtOAc (1.0 mL) and the layers were separated. The aqueous layer was extracted with EtOAc (3 x 1 mL) and the combined organic layers were dried by passage through a plug of anhydrous $MgSO_4$ (10 mL of EtOAc eluent), before the volatiles were removed under reduced pressure. To ease purification, unreacted 4-methylphenylboronic acid, pinacol ester was converted to the corresponding boronic acid following a known procedure:[11] A scintillation vial containing the crude material and a magnetic stir bar was purged with N_2 for 5 min. The crude material was dissolved in 7.5:1 THF:H_2O (1.7 mL), and stirred for 5 min at 23 °C. The resultant mixture was then treated with $NaIO_4$ (193 mg, 0.9 mmol, 9.0 equiv) and stirred for 30 min at 23 °C, before being treated with 1.0 M HCl (0.3 mL) and stirred for an additional 17 h at this temperature. This mixture was then diluted with deionized H_2O (5 mL) and EtOAc (12 mL) and the layers were separated. The aqueous phase was extracted with EtOAc (3 x 6 mL), and the combined organic layers were washed sequentially with deionized H_2O (2 x 10 mL) and brine (10 mL), dried over Na_2SO_4, filtered, and the volatiles were removed under reduced pressure. Purification by preparative TLC (29:1 hexanes:EtOAc) generated ketone **15** (83% yield, average of two experiments) as a colorless oil. Ketone **15**: R_f 0.54 (5:1 hexanes:EtOAc). The spectral data match those previously reported in the literature.[12]

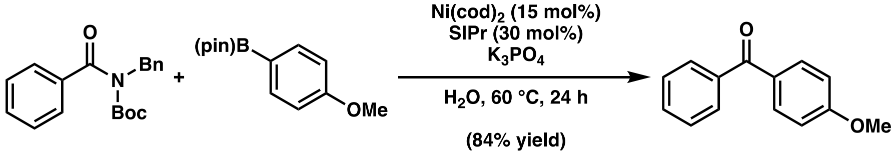

23 *SI-11* *16*

Ketone 16. Followed representative procedure A. Purification by sequential preparative TLC (9:1 hexanes:EtOAc eluted twice; 100% benzene, eluted three times) generated ketone **16** (84% yield, average of two experiments) as a light yellow oil. Ketone **16**: R_f 0.30 (9:1 hexanes:EtOAc). The spectral data match those previously reported in the literature.[4]

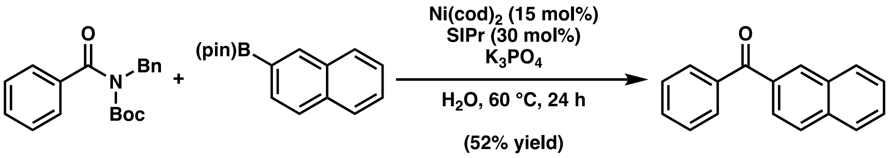

23 *SI-12* *17*

Ketone 17. Followed representative procedure A. Purification by sequential preparative TLC (9:1 hexanes:EtOAc, eluted twice; 5:1:1 hexanes:CH_2Cl_2:Et_2O; 100% benzene, eluted twice) generated ketone **17** (52% yield, average of two experiments) as a white solid. Ketone **17**: R_f 0.44 (9:1 hexanes:EtOAc). The spectral data match those previously reported in the literature.[13]

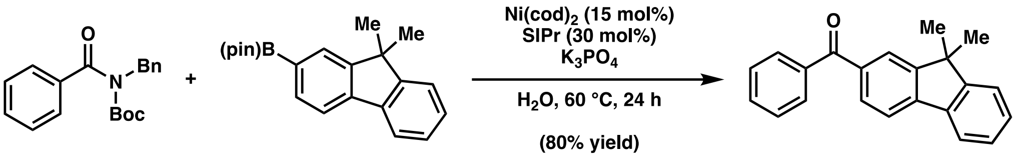

23 *SI-13* *18*

Ketone 18. Followed representative procedure A. Purification by sequential preparative TLC (9:1 hexanes:EtOAc, eluted twice; 100% benzene, eluted twice) generated ketone **18** (80% yield, average of two experiments) as a white solid. Ketone **18**: m.p. 122–124 ºC; R_f 0.40 (9:1 hexanes:EtOAc); ^{1}H NMR (500 MHz, $CDCl_3$): δ 7.95 (t, J = 1.15, 1H), 7.85–7.83 (m, 2H), 7.81–7.79 (m, 1H), 7.79 (d, J = 1.09, 2H), 7.61 (tt, J = 7.45, 1.36, 1H), 7.53–7.51 (m, 2H), 7.50–7.48 (m, 1H), 7.41–7.37 (m, 2H), 1.53 (s, 6H); ^{13}C NMR (125 MHz, $CDCl_3$): δ 196.9, 154.9, 153.8, 143.8, 138.4, 138.1, 136.4, 132.3, 130.3, 130.1, 128.7, 128.4, 127.4, 124.5, 123.0, 121.1, 119.6, 47.2, 27.1; IR (film): 3064, 2950, 2920, 2856, 1654, 1606, 1471, 1446, 1317, 1302, 1278, 1242, 759, 739, 722, 698, 684 cm^{-1}; HRMS-APCI (m/z) $[M + H]^+$ calcd for $C_{22}H_{19}O^+$, 299.1430; found 299.1418.

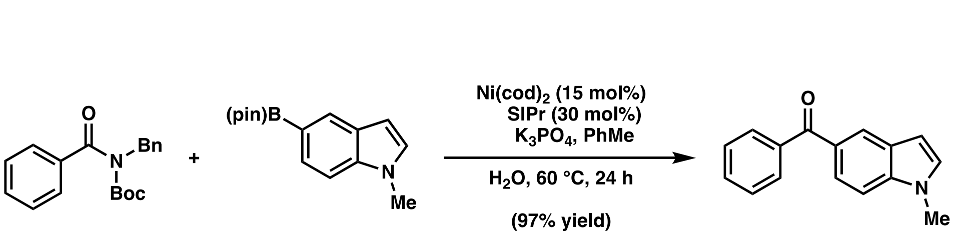

23 *SI-14* *19*

Ketone 19. Followed representative procedure B. Purification by sequential preparative TLC (9:1 hexanes:EtOAc; 5:1:1 hexanes:Et_2O:CH_2Cl_2; 100% benzene, eluted three times) generated ketone **19** (97% yield, average of two experiments) as a colorless oil. Ketone **19**: m.p. 82–84 ºC; R_f 0.14

(9:1 hexanes:EtOAc); ^{1}H NMR (500 MHz, $CDCl_3$): δ 8.11 (dd, J = 1.74, 0.64, 1H), 7.83 (dd, J = 8.68, 1.60, 1H), 7.81–7.79 (m, 2H), 7.57 (tt, J = 7.47, 1.36, 1H), 7.50–7.47 (m, 2H), 7.39 (d, J = 8.69, 1H), 7.14 (d, J = 3.12, 1H), 6.58 (dd, J = 3.20, 0.81, 1H), 3.86 (s, 3H); ^{13}C NMR (125 MHz, $CDCl_3$): δ 197.4, 139.3, 139.1, 131.7, 130.5, 130.0, 129.3, 128.2, 127.8, 125.6, 124.0, 109.2, 103.1, 33.2; IR (film): 1645, 1605, 1446, 1318, 1273, 1095, 729, 671 cm^{-1}; HRMS-APCI (m/z) $[M + H]^+$ calcd for $C_{16}H_{14}NO^+$, 236.1070; found 236.1060.

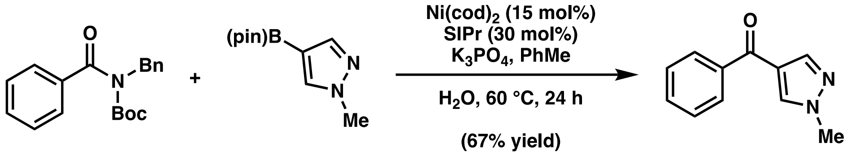

Ketone 20. Followed representative procedure B. Purification by sequential preparative TLC (1:1 hexanes:EtOAc; 1:1:1 hexanes:CH_2Cl_2:Et_2O) generated ketone **20** (67% yield, average of two experiments) as a colorless oil. Ketone **20**: R_f 0.31 (1:1 hexanes:EtOAc). The spectral data match those previously reported in the literature.[14]

D. Evaluation of KREDs

Note: The following protocol was adapted from the Codex® KRED Screening Kit – Screening Procedure P.

Representative Procedure for evaluating KREDs. Alcohol 21 (Supplementary Table 2, entry 1):

A 1-dram vial containing a magnetic stir bar was charged with KRED-401 (10 mg). To the vial was added Recycle Mix P (18.2 mg) as a solution in deionized H_2O (1.0 mL). The resultant mixture was stirred slowly at 23 °C until complete dissolution of the enzyme was observed. 2-Methylbenzophenone (**14**, 4.92 mg, 0.025 mmol, 1.0 equiv) was added to the mixture as a solution in i-PrOH (0.1 mL). The vial was then capped with a Teflon-lined screw cap and stirred at 35 °C and a rate of 450 RPM for 24 h. After cooling to 23 °C, the mixture was diluted with EtOAc (2.0 mL) and the layers were allowed to separate. The aqueous layer was extracted with EtOAc (3 x 2 mL) and the combined organic layers were dried by passage through a plug of anhydrous $MgSO_4$ (10 mL of EtOAc eluent). The volatiles were removed under reduced pressure and the crude

residue was purified by preparative TLC (10:1 hexanes:EtOAc) to yield alcohol **21** (1.4 mg, 28% yield, 87% ee) as a white solid. The spectral data match those previously reported in the literature for ***(R)*-21**.[15]

*Absolute stereochemistry was assigned by comparison with published $[\alpha]_D$ values for alcohols **(R)-21**[16] and **(R)-22**.[17]*

Screening efforts that deviate from the above conditions are indicated below.

Supplementary Table 2. Evaluation of KREDs in the reduction of ketones **14** and **16**.

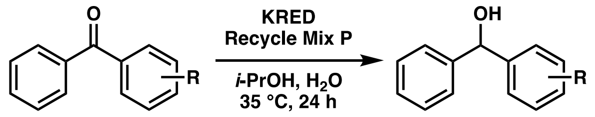

14; (R = o-Me)
16; (R = p-OMe)

21; (R = o-Me)
22; (R = p-OMe)

Entry	*R*	*KRED*	*Yield*	*ee*	*(R/S)*
1	2-Me	401	28%	87%	*(S)*
2	2-Me	402	33%	76%	*(S)*
3	2-Me	403	42%	80%	*(S)*
4	2-Me	404	59%	87%	*(S)*
5	2-Me	P1-B12	55%	99%	*(R)*
6	4-OMe	401	54%	39%	*(R)*
7	4-OMe	402	56%	8%	*(R)*
8	4-OMe	403	63%	3%	*(R)*
9	4-OMe	404	60%	9%	*(S)*
10	4-OMe	P1-B12	56%	94%	*(S)*

E. One-Pot Reaction Optimization Experiments

Representative Procedure for the Suzuki–Miyaura coupling (step 1, Supplementary Table 3, entries 1–5):

A 2-dram vial containing a magnetic stir bar was flame-dried under reduced pressure and then allowed to cool under N_2. The vial was charged with amide substrate **23** (31.1 mg, 0.1 mmol, 1.0 equiv), 2-methylphenylboronic acid, pinacol ester (**24**, 65.4 mg, 0.3 mmol, 3.0 equiv), and K_3PO_4 (42.4 mg, 0.2 mmol, 2.0 equiv). The vial was flushed with N_2 for 5 min and then taken into a glove box. In the glove box, the vial was charged with SIPr (11.7 mg, 0.03 mmol, 30 mol%) and $Ni(cod)_2$ (4.2 mg, 0.015 mmol, 15 mol%). Subsequently, the vial was removed from the glove box and degassed H_2O (0.2 mL, 0.5 M) was added. The vial was then capped with a Teflon-lined screw cap under a flow of N_2, sealed with Teflon tape, and stirred at 60 °C for 24 h.

*For all experiments in Supplementary Table **3**, the above procedure was employed for the first step (Suzuki–Miyaura coupling). Table entries vary only in the protocol used for the second step (enzymatic reduction) of the reaction, as described in the following Representative Procedures A–C.*

Representative Procedure A for optimization of the KRED reduction (step 2). Alcohol 21 (Supplementary Table 3, entry 1): The crude reaction mixture from step 1 was removed from heat and cooled to 23 °C. To the reaction mixture was added *i*-PrOH (400 µL, 52.0 equiv) and the resultant mixture was stirred for 5 min before stopping stirring and allowing the layers to separate in the reaction vessel. Recycle Mix P (78.2 mg) and KRED-P1-B12 (40.0 mg) were then added as a single solution in deionized H_2O (4 mL) with stirring at 23 °C. The reaction was heated to 35 °C and stirred at a rate of 450 RPM for 24 h.

The reaction mixture was cooled to 23 °C and transferred to a separatory funnel with deionized H_2O (15 mL), brine (5 mL), and EtOAc (30 mL). The layers were separated and the aqueous layer was extracted with EtOAc (3 x 30 mL). The combined organic layers were dried over anhydrous Na_2SO_4, filtered, and the volatiles were removed under reduced pressure. The yield was determined by ^{1}H NMR analysis with 1,3,5-trimethoxybenzene as an external standard. The spectral data match those previously reported in the literature for ***(R)*-21**.[15]

Representative Procedure B for optimization of the KRED reduction (step 2). Alcohol 21 (Supplementary Table 3, entries 2–4): The crude reaction mixture from step 1 was removed from heat and cooled to 23 °C before being neutralized with 1.0 M HCl (0.28 mL). To the reaction mixture was added *i*-PrOH (400 µL, 52.0 equiv) and the resultant mixture was stirred for 5 min before stopping stirring and allowing the layers to separate in the reaction vessel. Recycle Mix P (78.2 mg) and KRED-P1-B12 (40.0 mg) were then added as a single solution in deionized H_2O (4 mL). The reaction was then heated to 35 °C, placed under reduced pressure (350 mbar, see In-Hood Pump Set-Up and Specifications), and the mixture was stirred at a rate of 450 RPM for 24 h.

The vacuum was relieved, the reaction mixture was cooled to 23 °C, and transferred to a separatory funnel with deionized H_2O (15 mL), brine (5 mL), and EtOAc (30 mL). The layers were separated and the aqueous layer was extracted with EtOAc (3 x 30 mL). The combined organic layers were dried over anhydrous Na_2SO_4, filtered, and the volatiles were removed under reduced pressure. The yield was determined by ^1H NMR analysis with 1,3,5-trimethoxybenzene as an external standard. The spectral data match those previously reported in the literature for ***(R)*-21**.[15]

Representative Procedure C for optimization of the KRED reduction (step 2). Alcohol *(R)*-21 (Supplementary Table 3, entry 5): The crude reaction mixture from step 1 was removed from heat and cooled to 23 °C before being neutralized with 1.0 M HCl (0.28 mL). To the reaction mixture was added *i*-PrOH (400 µL, 52.0 equiv) and the resultant mixture was stirred for 5 min before stopping stirring and allowing the layers to separate in the reaction vessel. Recycle Mix P (78.2 mg) and KRED-P1-B12 (40.0 mg) were then added as a single solution in deionized H_2O (4 mL). The reaction was heated to 35 °C, placed under reduced pressure (350 mbar, see In-Hood Pump Set-Up and Specifications), and the mixture was stirred at a rate of 900 RPM for 24 h.

After 24 h, the vacuum was relieved and $Na_2NADP(+) \cdot 3H_2O$ (3.7 mg, 0.044 equiv) was added. The reaction was again heated to 35 °C, the pressure of the reaction vessel was reduced to 350 mbar using an in-hood vacuum pump, and the mixture was stirred at a rate of 900 RPM for an additional 24 h.

The vacuum was relieved, the reaction was cooled to 23 °C, and the reaction mixture was transferred to a separatory funnel with deionized H_2O (15 mL), brine (5 mL), and EtOAc (30 mL). The layers were separated and the aqueous layer was extracted with EtOAc (3 x 30 mL). The

combined organic layers were dried over anhydrous Na_2SO_4, filtered, and the volatiles were removed under reduced pressure. The yield was determined by ^1H NMR analysis with 1,3,5-trimethoxybenzene as an external standard. The spectral data match those previously reported in the literature for ***(R)*-21**.[15]

Optimization efforts that deviate from the above conditions are indicated below.

Supplementary Table 3. Optimization of the KRED reduction (step 2) in the sequential, one-pot reaction.[a]

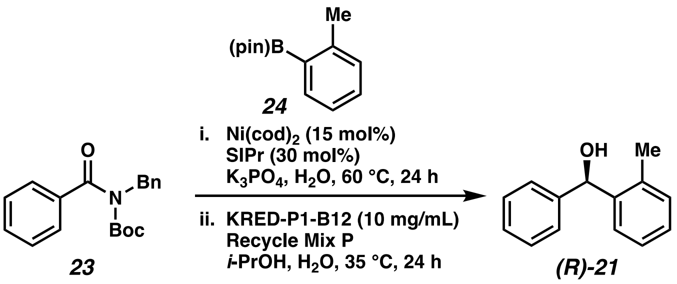

Entry	*Rep. Procedure*	*Pressure*	*Stir Rate*	*Time*	*NADP(+)*	*Yield*
**1**	**A**	**>1000 mbar**	**450 RPM**	**24 h**	**–**	**17%**
**2**	**B**	**350 mbar**	**450 RPM**	**24 h**	**–**	**27%**
**3**	**B**	**350 mbar**	**900 RPM**	**24 h**	**–**	**34%**
**4**	**B**	**350 mbar**	**900 RPM**	**48 h**	**–**	**48%**
**5**	**C**	**350 mbar**	**900 RPM**	**48 h**	**0.044 equiv after 24 h**	**63%**

[a] Yields were determined using ^1H NMR analysis with 1,3,5-trimethoxybenzene (TMB) as an external standard.

F. Scope of the One-Pot Reaction

Representative Procedure A for the one-pot, chemoenzymatic alcohol synthesis from amides (Figures 3 and 4, synthesis of alcohol *(R)*-25 used as an example).

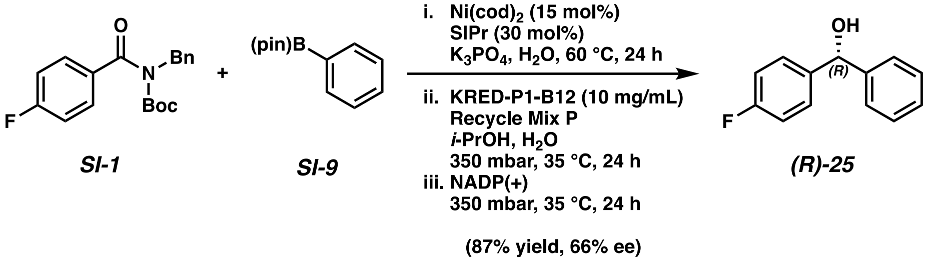

Alcohol *(R)*-25. A 2-dram vial containing a magnetic stir bar was flame-dried under reduced pressure and then allowed to cool under N_2. The vial was charged with amide substrate **SI-1** (32.9 mg, 0.1 mmol, 1.0 equiv), phenylboronic acid, pinacol ester (**SI-9**, 61.2 mg, 0.3 mmol, 3.0 equiv), and K_3PO_4 (42.4 mg, 0.2 mmol, 2.0 equiv). The vial was flushed with N_2 and then taken into a glove box. In the glove box, the vial was charged with SIPr (11.7 mg, 0.03 mmol, 30 mol%) and $Ni(cod)_2$ (4.2 mg, 0.015 mmol, 15 mol%). Subsequently, the vial was removed from the glove box and degassed H_2O (0.2 mL, 0.5 M) was added. The vial was then capped with a Teflon-lined screw cap under a flow of N_2, sealed with Teflon tape, and stirred at 60 °C for 24 h.

The crude reaction mixture was removed from heat and cooled to 23 °C before being neutralized with 1.0 M HCl (0.28 mL). To the reaction mixture was added *i*-PrOH (400 µL, 52.0 equiv) and the resultant mixture was stirred for 5 min before stopping stirring and allowing the layers to separate in the reaction vessel. Recycle Mix P (78.2 mg) and KRED-P1-B12 (40.0 mg) were then added as a single solution in deionized H_2O (4 mL). The reaction was then heated to 35 °C, placed under reduced pressure (350 mbar, see In-Hood Pump Set-Up and Specifications), and the mixture was stirred at a rate of 900 RPM for 24 h.

After 24 h, the vacuum was relieved and $Na_2NADP(+)\cdot 3H_2O$ (3.7 mg, 0.044 equiv) was added. The reaction was again heated to 35 °C, placed under reduced pressure (350 mbar, see In-Hood Pump Set-Up and Specifications), and the mixture was stirred at a rate of 900 RPM for an additional 24 h.

The vacuum was relieved, the reaction was cooled to 23 °C, and the reaction mixture was transferred to a separatory funnel with deionized H_2O (15 mL), brine (5 mL), and EtOAc (30 mL). The layers were separated and the aqueous layer was extracted with EtOAc (3 x 30 mL). The combined organic layers were dried over anhydrous Na_2SO_4, filtered, and the volatiles were removed under reduced pressure.

The crude material was purified by preparative TLC (plate neutralized with a 2% Et_3N in hexanes solution prior to loading crude material; 17:2:1 hexanes:benzene:Et_2O) to yield alcohol ***(R)*-25** (87% yield, average of two experiments, 66% ee) as a white solid. Alcohol ***(R)*-25**: $[\alpha]_D^{22.7}$ = –7.60° (*c* = 1.00, $CHCl_3$); R_f 0.23 (5:1 hexanes:EtOAc). Chiral SFC: 250 mm CHIRALPAK IC–3, 1% *i*-PrOH, 3.5 mL/min, λ = 216 nm, 35 °C, nozzle pressure = 100 bar CO_2, t_{R1} (major) = 17.9 min, t_{R2} (minor) = 19.5 min. The spectral data match those previously reported in the literature for ***(R)*-25**.[17] The major enantiomer product was assigned by comparison to published $[\alpha]_D$ values for ***(R)*-25**.[17]

Representative Procedure B for the one-pot chemoenzymatic alcohol synthesis from amides (Figure 3, synthesis of alcohol *(R)*-26 used as an example).

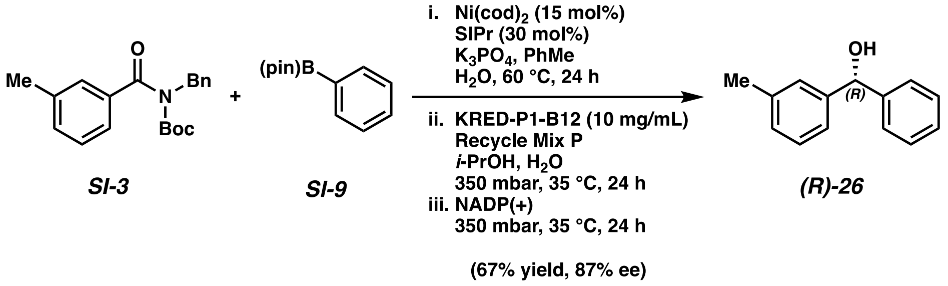

Alcohol *(R)*-26. A 2-dram vial containing a magnetic stir bar was flame-dried under reduced pressure and then allowed to cool under N_2. The vial was charged with amide substrate **SI-3** (32.5 mg, 0.1 mmol, 1.0 equiv), phenylboronic acid, pinacol ester (**SI-9**, 61.2 mg, 0.3 mmol, 3.0 equiv), and K_3PO_4 (42.4 mg, 0.2 mmol, 2.0 equiv). The vial was flushed with N_2 and then taken into a glove box. In the glove box, the vial was charged with SIPr (11.7 mg, 0.03 mmol, 30 mol%), $Ni(cod)_2$ (4.2 mg, 0.015 mmol, 15 mol%), and PhMe (50 µL). Subsequently, the vial was removed from the glove box and degassed H_2O (0.2 mL, 0.5 M) was added. The vial was then capped with

a Teflon-lined screw cap under a flow of N_2, sealed with Teflon tape, and stirred at 60 °C for 24 h.

The crude reaction mixture was removed from heat and cooled to 23 °C before being neutralized with 1.0 M HCl (0.28 mL). To the reaction mixture was added *i*-PrOH (400 µL, 52.0 equiv) and the resultant mixture was stirred for 5 min before stopping stirring and allowing the layers to separate in the reaction vessel. Recycle Mix P (78.2 mg) and KRED-P1-B12 (40.0 mg) were then added as a single solution in deionized H_2O (4 mL). The reaction was then heated to 35 °C, placed under reduced pressure (350 mbar, see In-Hood Pump Set-Up and Specifications), and the mixture was stirred at a rate of 900 RPM for 24 h.

After 24 h, the vacuum was relieved, the reaction was cooled to 23 °C, and $Na_2NADP(+){\bullet}3H_2O$ (3.7 mg, 0.044 equiv) was added. The reaction was again heated to 35 °C, the pressure of the reaction vessel was reduced to 350 mbar using an in-hood vacuum pump, and the mixture was stirred at a rate of 900 RPM for an additional 24 h.

The vacuum was relieved, the reaction was cooled to 23 °C, and the reaction mixture was transferred to a separatory funnel with deionized H_2O (15 mL), brine (5 mL), and EtOAc (30 mL). The layers separated and the aqueous layer was extracted with EtOAc (3 x 30 mL). The combined organic layers were dried over anhydrous Na_2SO_4, filtered, and the volatiles were removed under reduced pressure.

The crude material was purified by preparative TLC (plate neutralized with a 2% Et_3N in hexanes solution prior to loading crude material; 17:2:1 hexanes:benzene:Et_2O) to yield alcohol ***(R)*-26** (67% yield, average of two experiments, 87% ee) as a white solid. Alcohol ***(R)*-26**: $[\alpha]_D^{25.4}$ = +2.30° (c = 1.00, CH_2Cl_2); R_f 0.35 (5:1 hexanes:EtOAc). Chiral SFC: 250 mm CHIRALPAK AD–3, 5% MeOH, 3.5 mL/min, λ = 216 nm, 35 °C, nozzle pressure = 100 bar CO_2, t_{R1} (minor) = 7.0 min, t_{R2} (major) = 7.6 min. The spectral data match those previously reported in the literature for ***rac*-26**.[18] The major enantiomer product was assigned by comparison to published $[\alpha]_D$ values for ***(R)*-26**.[19]

Any modifications of the conditions shown in the representative procedures above are specified in the following schemes and text.

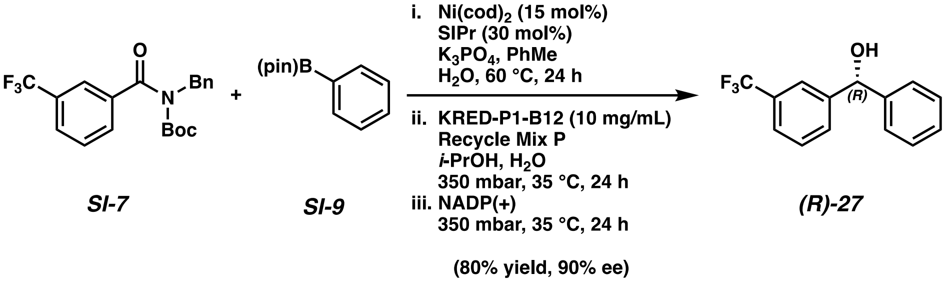

Alcohol *(R)*-27: Followed representative procedure B. Purification by preparative TLC (plate neutralized with a 2% Et_3N in hexanes solution prior to loading crude material; 17:2:1 hexanes:benzene:Et_2O) yielded alcohol ***(R)*-27** (80% yield, average of two experiments, 90% ee) as a colorless oil. Alcohol ***(R)*-27**: $[\alpha]_D^{25.5}$ = –15.80° (c = 1.00, $CHCl_3$); R_f 0.30 (5:1 hexanes:EtOAc). Chiral SFC: 250 mm CHIRALPAK IB–H, 2% MeOH, 3.5 mL/min, λ = 216 nm, 35 °C, nozzle pressure = 100 bar CO_2, t_{R1} (minor) = 10.9 min, t_{R2} (major) = 11.8 min. The spectral data match those previously reported in the literature for ***rac*-27**.[20] The major enantiomer product was assigned by comparison to published $[\alpha]_D$ values for ***(R)*-27**.[21]

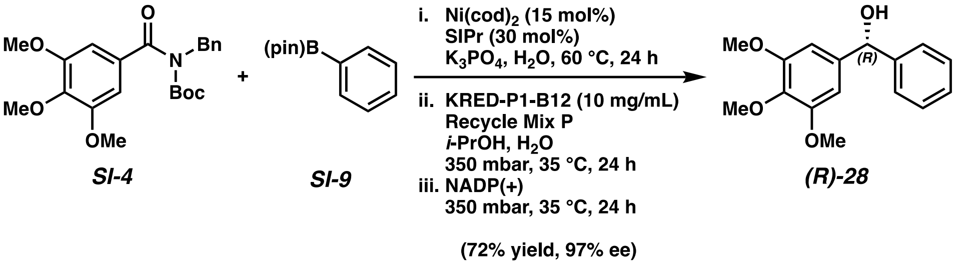

Alcohol *(R)*-28: Followed representative procedure A. Purification by preparative TLC (plate neutralized with a 2% Et_3N in hexanes solution prior to loading crude material; 3:2:1 hexanes:benzene:Et_2O) yielded alcohol ***(R)*-28** (72% yield, average of two experiments, 97% ee) as a white solid. Alcohol ***(R)*-28**: $[\alpha]_D^{23.2}$ = –8.80° (c = 1.00, $CHCl_3$); R_f 0.24 (2:1 hexanes:EtOAc). Chiral SFC: 250 mm CHIRALPAK AD–3, 15% *i*-PrOH, 3.5 mL/min, λ = 216 nm, 35 °C, nozzle pressure = 100 bar CO_2, t_{R1} (minor) = 6.0 min, t_{R2} (major) = 7.2 min. The spectral data match those previously reported in the literature for ***rac*-28**.[22] The major enantiomer product was assigned by comparison to the $[\alpha]_D$ and Chiral SFC retention time values obtained for ***(S)*-28** as described in Section Gii of the Experimental Procedures.

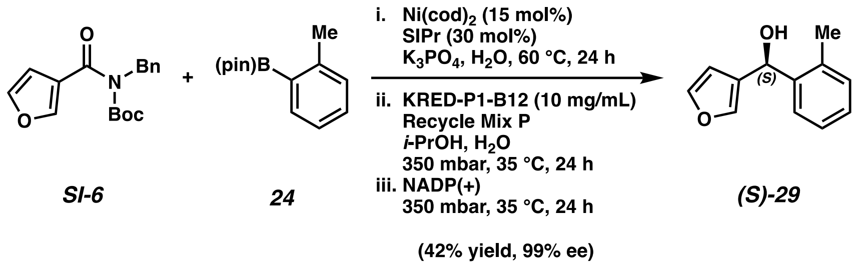

Alcohol *(S)*-29: Followed representative procedure A. Purification by preparative TLC (plate neutralized with a 2% Et_3N in hexanes solution prior to loading crude material; 9:1 hexanes:EtOAc) yielded alcohol ***(S)*-29** (42% yield, average of two experiments, 99% ee) as a colorless oil. Alcohol ***(S)*-29**: $[\alpha]_D^{26.8}$ = +8.00° (*c* = 0.100, $CHCl_3$); R_f 0.31 (4:1 hexanes:EtOAc); ^1H NMR (500 MHz, $CDCl_3$): 7.54 (dd, *J* = 7.5, 1.3, 1H), 7.37 (t, *J* = 1.70, 1H), 7.25–7.19 (overlap, 3H), 7.17–7.13 (m, 1H), 6.34 (dd, *J* = 1.9, 0.76, 1H), 5.97 (d, *J* = 3.8, 1H), 2.30 (s, 3H), 2.01 (d, *J* = 4.25, 1H); ^{13}C NMR (125 MHz, $CDCl_3$): δ 143.6, 141.1, 140.3, 135.1, 130.7, 128.3, 127.8, 126.4, 125.8, 109.6, 66.5, 19.2; IR (film): 3359, 2958, 2926, 2854, 1664, 1501, 1157 cm^{-1}; HRMS-APCI (*m/z*) $[M]^+$ calcd for $C_{12}H_{12}O_2^+$, 188.0831; found 188.0824. Chiral SFC: 250 mm CHIRALPAK AD–H, 5% *i*-PrOH, 3.5 mL/min, λ = 210 nm, 40 °C, nozzle pressure = 100 bar CO_2, t_{R1} (major) = 8.2 min, t_{R2} (minor) = 9.8 min. The major enantiomer product was assigned by analogy to the proposed selectivity model outlined in Figure 4B.

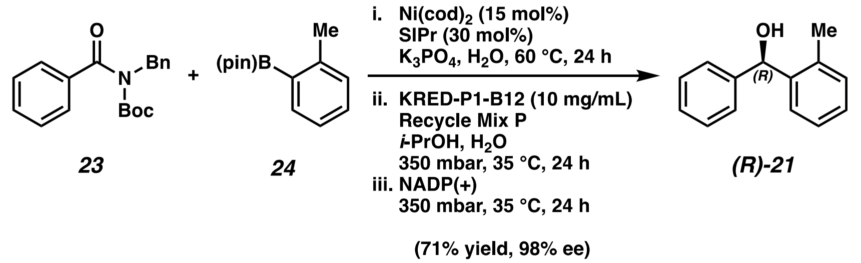

Alcohol *(R)*-21: Followed representative procedure A. Purification by preparative TLC (plate neutralized with a 2% Et_3N in hexanes solution prior to loading crude material; 17:2:1 hexanes:benzene:Et_2O) yielded alcohol ***(R)*-21** (71% yield, average of two experiments, 98% ee) as a white solid. Alcohol ***(R)*-21**: $[\alpha]_D^{24.3}$ = –5.67° (*c* = 1.00, $CHCl_3$); R_f = 0.31 (5:1 hexanes:EtOAc). Chiral SFC: 250 mm CHIRALPAK IC–3, 5% *i*-PrOH, 3.5 mL/min, λ = 216 nm, 35 °C, nozzle pressure = 100 bar CO_2, t_{R1} (minor) = 8.4 min, t_{R2} (major) = 9.3 min. The spectral

data match those previously reported in the literature for ***(R)*-21**.[15] The major enantiomer product was assigned by comparison to published $[\alpha]_D$ values for ***(R)*-21**.[16]

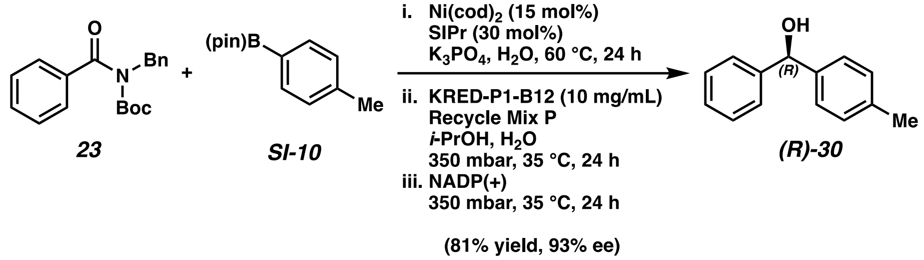

Alcohol *(R)*-30: Followed representative procedure A. Purification by preparative TLC (plate neutralized with a 2% Et_3N in hexanes solution prior to loading crude material; 17:2:1 hexanes:benzene:Et_2O) yielded alcohol ***(R)*-30** (81% yield, average of two experiments, 93% ee) as a white solid. Alcohol ***(R)*-30**: $[\alpha]_D^{23.3}$ = +8.60° (*c* = 1.00, $CHCl_3$); R_f = 0.41 (5:1 hexanes:EtOAc). Chiral SFC: 250 mm CHIRALPAK AD–3, 5% MeOH, 3.5 mL/min, λ = 216 nm, 35 °C, nozzle pressure = 100 bar CO_2, t_{R1} (minor) = 9.5 min, t_{R2} (major) = 10.1 min. The spectral data match those previously reported in the literature for ***(R)*-30**.[17] The major enantiomer product was assigned by comparison to published $[\alpha]_D$ values for ***(R)*-30**.[17]

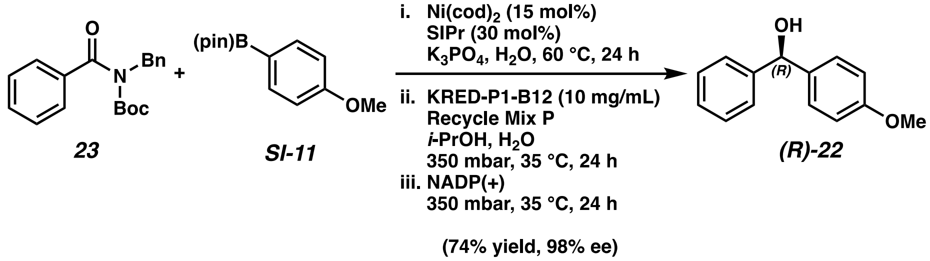

Alcohol *(R)*-22: Followed representative procedure A. Purification by preparative TLC (plate neutralized with a 2% Et_3N in hexanes solution prior to loading crude material; 12:2:1 hexanes:benzene:Et_2O) yielded alcohol ***(R)*-22** (74% yield, average of two experiments, 98% ee) as a white solid. Alcohol ***(R)*-22**: $[\alpha]_D^{27.2}$ = +14.40° (*c* = 1.00, $CHCl_3$); R_f 0.18 (5:1 hexanes:EtOAc). Chiral SFC: 250 mm CHIRALPAK IA–3, 5% *i*-PrOH, 3.5 mL/min, λ = 216 nm, 35 °C, nozzle pressure = 100 bar CO_2, t_{R1} (major) = 17.7 min, t_{R2} (minor) = 19.3 min. The spectral data match those previously reported in the literature for ***(S)*-22**.[17] The major enantiomer product was assigned by comparison to published $[\alpha]_D$ values for ***(R)*-22**.[17]

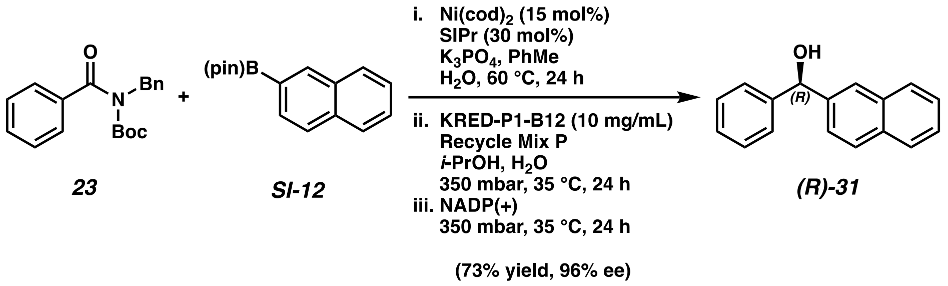

Alcohol *(R)*-31: Followed representative procedure B. Purification by preparative TLC (plate neutralized with a 2% Et_3N in hexanes solution prior to loading crude material; 17:2:1 hexanes:benzene:Et_2O, eluted twice) yielded alcohol ***(R)*-31** (73% yield, average of two experiments, 96% ee) as a white solid. Alcohol ***(R)*-31**: $[\alpha]_D^{25.2}$ = +4.50° (*c* = 1.00, C_6H_6); R_f 0.26 (5:1 hexanes:EtOAc). Chiral SFC: 250 mm CHIRALPAK IB–H, 20% *i*-PrOH, 3.5 mL/min, λ = 210 nm, 40 °C, nozzle pressure = 100 bar CO_2, t_{R1} (minor) = 4.1 min, t_{R2} (minor) = 4.7 min. The spectral data match those previously reported in the literature for ***(R)*-31**.[23] The major enantiomer product was assigned by comparison to published $[\alpha]_D$ values for ***(R)*-31**.[24]

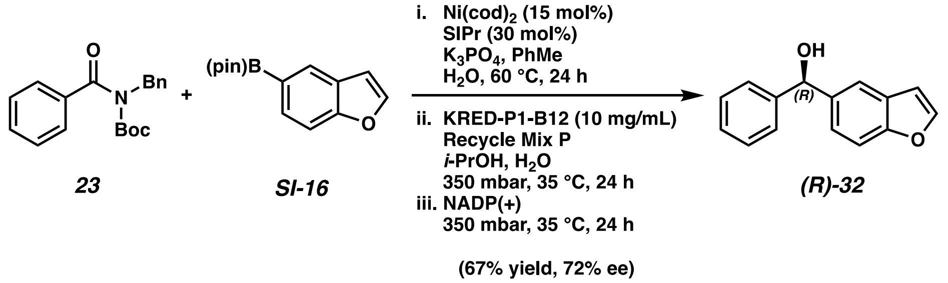

Alcohol *(R)*-32: Followed representative procedure B. Purification by preparative TLC (plate neutralized with a 2% Et_3N in hexanes solution prior to loading crude material; 17:2:1 hexanes:benzene:Et_2O, eluted twice) yielded alcohol ***(R)*-32** (67% yield, average of two experiments, 72% ee) as a clear oil. Alcohol ***(R)*-32**: m.p. 67–70 °C $[\alpha]_D^{24.3}$ = +3.81° (*c* = 0.7, C_6H_6); R_f 0.21 (5:1 hexanes:EtOAc); ^1H NMR (500 MHz, $CDCl_3$): 7.64–7.61 (m, 2H), 7.47–7.44 (m, 1H), 7.43–7.40 (m, 2H), 7.36–7.32 (m, 2H), 7.31 (dd, *J* = 8.5, 1.9, 1H), 7.29–7.25 (m, 1H), 6.74 (dd, *J* = 2.3, 0.97, 1H), 5.97 (d, *J* = 2.1, 1H), 2.24 (d, *J* = 3.2, 1H); ^{13}C NMR (125 MHz, $CDCl_3$): (12 of 13 observed) δ 154.4, 145.5, 144.1, 138.7, 128.5, 127.5, 126.5, 123.3, 119.3, 111.4, 106.7, 76.4; IR (film): 3352, 3028, 1467, 1451, 1262, 1031 cm^{-1}; HRMS-APCI (*m/z*) $[M+H]^+$

calcd for $C_{15}H_{13}O_2^+$, 225.0910; found 225.0907. Chiral SFC: 250 mm CHIRALPAK OJ–H, 15% *i*-PrOH, 3.5 mL/min, λ = 210 nm, 40 °C, nozzle pressure = 100 bar CO_2, t_{R1} (major) = 7.5 min, t_{R2} (minor) = 8.7 min. The major enantiomer product was assigned by analogy to the selectivity model outlined in Figure 4B.

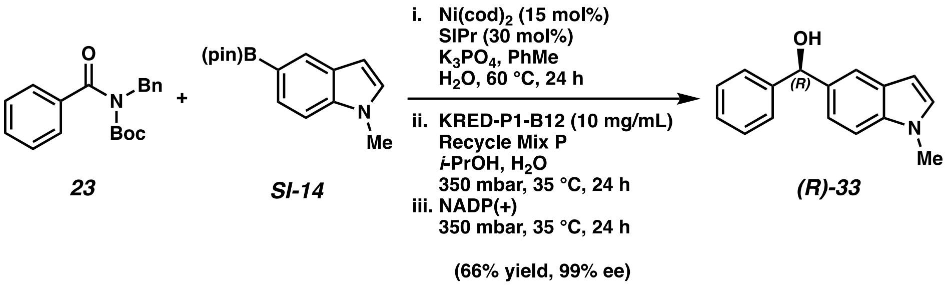

Alcohol *(R)*-33: Followed representative procedure B. Purification by preparative thin layer chromatography (plate neutralized with a 2% Et_3N in hexanes solution prior to loading crude material; 7:2:1 hexanes:benzene:Et_2O) yielded alcohol ***(R)*-33** (66% yield, average of two experiments, 99% ee) as an off-white solid. Alcohol ***(R)*-33**: $[\alpha]_D^{27.1}$ = +26.40° (c = 1.00, $CHCl_3$); R_f 0.15 (5:1 hexanes:EtOAc). Chiral SFC: 250 mm CHIRALPAK AS–H, 20% *i*-PrOH, 3.5 mL/min, λ = 216 nm, 35 °C, nozzle pressure = 100 bar CO_2, t_{R1} (major) = 2.9 min, t_{R2} (minor) = 3.3 min. The spectral data match those obtained for ***rac*-33** as described in Section Gi of the Experimental Procedures. The major enantiomer product was assigned by comparison to the $[\alpha]_D$ and Chiral SFC retention time values obtained for ***(R)*-33** as described in Section Gii of the Experimental Procedures.

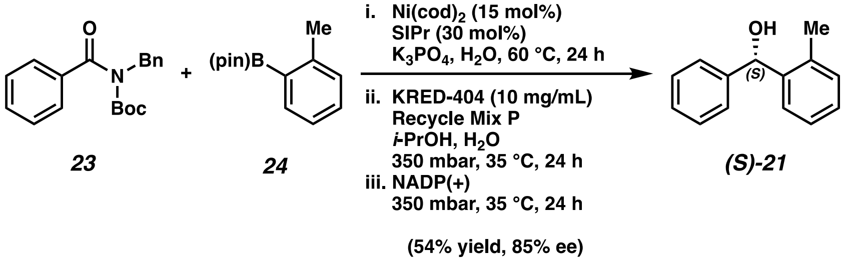

Alcohol *(S)*-21: Followed representative procedure A using KRED-404 in place of KRED-P1-B12. Purification by preparative TLC (plate neutralized with a 2% Et_3N in hexanes solution prior to loading crude material; 17:2:1 hexanes:benzene:Et_2O) yielded alcohol ***(S)*-21** (54% yield,

average of two experiments, 85% ee) as a white solid. Alcohol ***(S)*-21**: $[\alpha]_D^{23.0}$ = +6.31° (*c* = 1.00, $CHCl_3$); R_f 0.31 (5:1 hexanes:EtOAc). Chiral SFC: 250 mm CHIRALPAK IC–3, 5% *i*-PrOH, 3.5 mL/min, λ = 216 nm, 35 °C, nozzle pressure = 100 bar CO_2, t_{R1} (major) = 8.8 min, t_{R2} (minor) = 9.9 min. The spectral data match those previously reported in the literature for ***(R)*-21**.[15] The major enantiomer product was assigned by comparison to published $[\alpha]_D$ values for ***(S)*-21**.[16]

G. Verification of Enantiopurity

i. Preparation of Racemic Alcohols

Note: Alcohols ***rac*-21** and ***rac*-22** were obtained commercially (see Materials and Methods). Supplementary information for the syntheses of ketones **SI-17**[25] and **SI-18[26]** as well as alcohols ***rac*-25**[27] ***rac*-26**,[28] ***rac*-27**,[20] ***rac*-28**,[22] ***rac*-30**,[27] and ***rac*-31**[29] have been published and spectral data match those previously reported.

Procedures for the synthesis of *rac*-29, *rac*-32, and *rac*-33 used for SFC assay development.

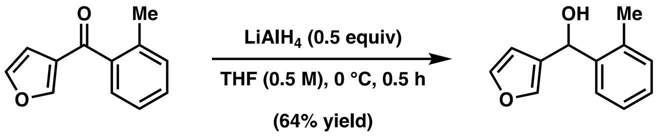

SI-17 *rac-29*

Alcohol *rac*-29. A 1-dram vial equipped with a magnetic stir bar was flame-dried under reduced pressure and then allowed to cool under N_2. To the vial was added ketone **SI-17** (5.30 mg, 28.5 μmol, 1.0 equiv) and THF (0.055 mL, 0.4 M). The resultant solution was cooled to 0 °C and stirring began. At 0 °C, $LiAlH_4$ (14.2 μL, 14.2 μmol, 0.5 equiv, 1.0 M in THF) was added dropwise across 1 min and the reaction was left to stir at this temperature for 0.5 h.

The reaction was quenched at 0 °C with deionized H_2O (1.0 mL) and subsequently warmed to 23 °C. The reaction mixture was then extracted with CH_2Cl_2 (3 x 1 mL) and the combined organic layers were dried over Na_2SO_4, filtered, and the volatiles were removed under reduced pressure.

The crude material was purified by preparative TLC (4:1 hexanes:EtOAc) to generate alcohol ***rac*-29** (3.4 mg, 64% yield) as a clear oil. The spectral data match those obtained for **(*S*)-**

29 as described in section F. Chiral SFC: 250 mm CHIRALPAK AD–H, 5% *i*-PrOH, 3.5 mL/min, λ = 210 nm, 40 °C, nozzle pressure = 100 bar CO_2, t_{R1} = 9.8 min, t_{R2} = 8.3 min.

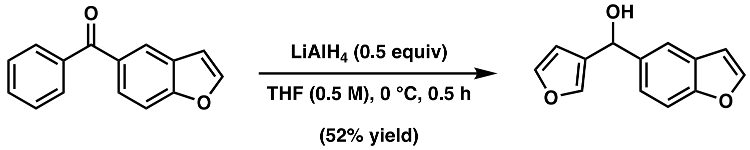

SI-18 *rac-32*

Alcohol *rac*-32. A 1-dram vial equipped with a magnetic stir bar was flame-dried under reduced pressure and then allowed to cool under N_2. To the vial was added ketone **SI-18** (6.10 mg, 27.4 μmol, 1.0 equiv) and THF (0.055 mL, 0.4 M). The resultant solution was cooled to 0 °C and stirring began. At 0 °C, $LiAlH_4$ (13.7 μL, 13.7 μmol, 0.5 equiv, 1.0 M in THF) was added dropwise across 1 min and the reaction was left to stir at this temperature for 0.5 h.

The reaction was quenched at 0 °C with deionized H_2O (1.0 mL) and subsequently warmed to 23 °C. The reaction mixture was then extracted with CH_2Cl_2 (3 x 1 mL) and the combined organic layers were dried over Na_2SO_4, filtered, and the volatiles were removed under reduced pressure.

The crude material was purified by preparative TLC (4:1 hexanes:EtOAc) to generate alcohol ***rac*-32** (3.2 mg, 52% yield) as a clear oil. The spectral data match those obtained for **(*S*)-32** as described in section F. Chiral SFC: 250 mm CHIRALPAK OJ–H, 15% *i*-PrOH, 3.5 mL/min, λ = 210 nm, 40 °C, nozzle pressure = 100 bar CO_2, t_{R1} = 8.6 min, t_{R2} = 7.5 min.

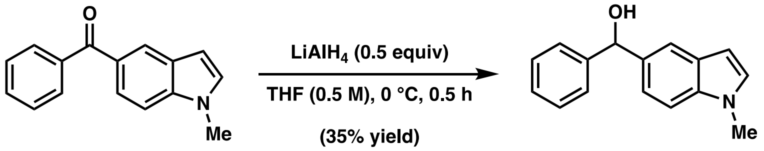

19 *rac-33*

Alcohol *rac*-33. A 1-dram vial equipped with a magnetic stir bar was flame-dried under reduced pressure and then allowed to cool under N_2. To the vial was added ketone **19** (20.0 mg, 85.0 μmol, 1.0 equiv) and THF (0.20 mL, 0.4 M). The resultant solution was cooled to 0 °C and stirring began. At 0 °C, $LiAlH_4$ (42.5 μL, 42.5 μmol, 0.5 equiv, 1.0 M in THF) was added dropwise across 1 min and the reaction was left to stir at this temperature for 0.5 h.

The reaction was quenched at 0 °C with deionized H_2O (1.0 mL) and subsequently warmed to 23 °C. The reaction mixture was then extracted with CH_2Cl_2 (3 x 1 mL) and the combined organic layers were dried over Na_2SO_4, filtered, and the volatiles were removed under reduced pressure.

The crude material was purified by preparative TLC (4:1 hexanes:EtOAc) to generate alcohol ***rac*-33** (7.1 mg, 35% yield) as a white film. Alcohol ***rac*-33**: R_f 0.21 (2:1 hexanes:EtOAc); ^{1}H NMR (500 MHz, $CDCl_3$): δ 7.64–7.62 (m, 1H), 7.45–7.41 (m, 2H), 7.35–7.30 (m, 2H), 7.30–7.27 (m, 1H), 7.25–7.20 (m, 2H), 7.05 (d, *J* = 3.2, 1H), 6.46 (dd, *J* = 3.1, 0.8, 1H), 5.98 (d, *J* = 3.4, 1H), 3.78 (s, 3H), 2.18 (d, *J* = 3.6, 1H); ^{13}C NMR (125 MHz, $CDCl_3$): δ 144.7, 136.4, 135.4, 129.5, 128.5, 128.4, 127.3, 126.6, 120.9, 119.3, 109.6, 101.3, 77.0, 33.1; IR (film): 3380, 3027, 2924, 1603, 1512, 1492, 1450 cm^{-1}; HRMS-APCI (*m/z*) $[M+H]^+$ calcd for $C_{16}H_{16}NO^+$, 238.1226; found 238.1226. Chiral SFC: 250 mm CHIRALPAK AS–H, 20% *i*-PrOH, 3.5 mL/min, λ = 216 nm, 35 °C, nozzle pressure = 100 bar CO_2, t_{R1} = 2.9 min, t_{R2} = 3.2 min.

ii. Enantioselective Diarylmethanol Synthesis by Asymmetric Arylation.

Note: Enantioenriched diarylmethanol products synthesized in this report for which $[\alpha]_D$ values have not been previously disclosed were synthesized via asymmetric arylation to facilitate stereochemical assignments.[30] For the conditions outlined below, originally reported by Braga[31] and modified by Jarvo,[32] arylation occurs from the *re* face of the prochiral aldehyde substrate.

Synthesis of the *(S)*-(1-tritylaziridin-2-yl)diphenylmethanol ligand SI-19.

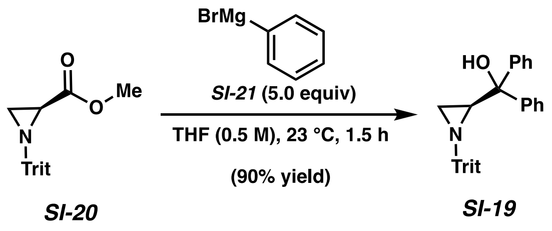

Ligand SI-19: A scintillation vial equipped with a magnetic stir bar was flame-dried under reduced pressure and then allowed to cool under N_2. To the vial was added *(S)*-methyl-1-tritylaziridine-2-carboxylate (**SI-20**, 150 mg, 0.437 mmol, 1.0 equiv) and the vial was flushed with N_2 for 5 min. THF (0.87 mL, 0.5 M) was then added and the mixture was stirred at 23 °C until homogeneous. Then, phenylmagnesium bromide (**SI-21**, 2.18 mL, 2.18 mmol, 5.0 equiv, 1.0 M in

THF) was added dropwise across 10 min and the solution was stirred at 23 °C for 1.5 h.

The reaction was quenched by the addition of sat. aq. NH_4Cl (6 mL) and the layers were separated. The aqueous layer was extracted with Et_2O (3 x 6 mL) and the combined organic layers were dried over Na_2SO_4, filtered, and the volatiles were removed under reduced pressure. The crude material was purified by flash chromatography (49:1 hexanes:EtOAc w/ 1% Et_3N) to yield ligand **SI-19** (182.8 mg, 90% yield) as a white foam. Ligand **SI-19**: $[\alpha]_D^{22.9}$ = –80.8° (c = 1.00, $CHCl_3$); R_f 0.60 (5:1 hexanes:EtOAc); The spectral data match those previously reported in the literature.[31]

Representative Procedure for asymmetric arylation (synthesis of *(S)*-28 used as an example).

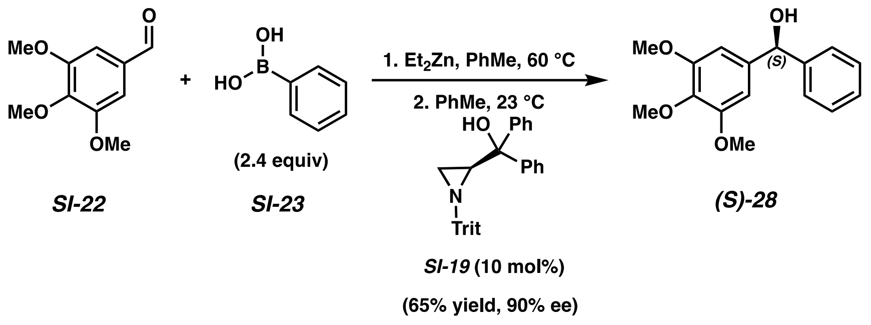

Alcohol *(S)*-28: A scintillation vial equipped with a magnetic stir bar was flame-dried under reduced pressure and then allowed to cool under N_2. To the vial was added phenylboronic acid (**SI-23**, 146.3 mg, 1.20 mmol, 2.4 equiv) and the vial was flushed with N_2 for 5 min before being taken into a glove box. In the glove box, toluene (2.50 mL) was added and the mixture was briefly stirred at 23 °C. $ZnEt_2$ (3.18 mL, 3.50 mmol, 7.0 equiv, 1.1 M in toluene) was then added and the septum cap was replaced with a Teflon-lined plastic screw cap. The vial was removed from the glove box, sealed with Teflon tape, and stirred at 60 °C for 12 h. The reaction was then removed from heat and cooled to 23 °C. Ligand **SI-19** (1.25 mL, 0.05 mmol, 0.1 equiv, 0.04 M in toluene) was then added and the reaction mixture was stirred at 23 °C for 10 min. Subsequently, 3,4,5-trimethoxybenzaldehyde (**SI-22**, 1.25 mL, 0.5 mmol, 1.0 equiv, 0.4 M in toluene) was added to the vial and the resultant solution was stirred at 23 °C for 3 h.

The reaction was neutralized by careful addition of 1.0 M HCl (~2.0 mL) at 23 °C, monitored by pH paper. The crude reaction mixture was then transferred to a separatory funnel

with deionized H_2O (20 mL), brine (10 mL), and EtOAc (30 mL). The layers were allowed to separate and the aqueous layer was extracted with EtOAc (2 x 30 mL). The combined organic layers were washed with brine (30 mL), dried over Na_2SO_4, filtered, and the volatiles were removed under reduced pressure.

The crude material was purified by three sequential flash chromatography columns (4:1 → 2:1 hexanes:EtOAc; 5:1:1 hexanes:benzene:Et_2O; 2:1:1 hexanes:CH_2Cl_2:Et_2O) to yield ***(S)*-28** (89.0 mg, 65% yield, 90% ee) as a white solid. Alcohol ***(S)*-28**: $[\alpha]_D^{24.8}$ = +5.60° (c = 1.00, $CHCl_3$); R_f 0.24 (2:1 hexanes:EtOAc). Chiral SFC: 250 mm CHIRALPAK AD–3, 15% *i*-PrOH, 3.5 mL/min, λ = 216 nm, 35 °C, nozzle pressure = 100 bar CO_2, t_{R1} (major) = 6.1 min, t_{R2} (minor) = 7.3 min. The spectral data match those previously reported in the literature for ***rac*-28**.[22]

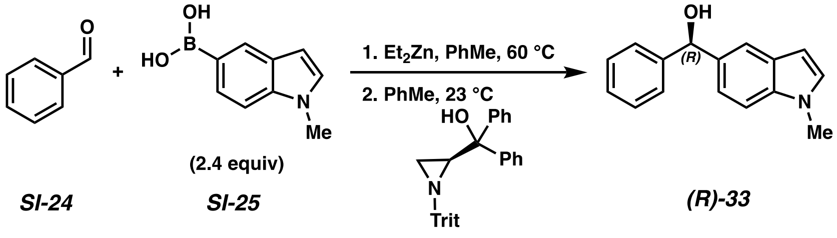

SI-24 *SI-25* *SI-19* (10 mol%) (94% yield, 72% ee) *(R)-33*

Alcohol *(R)*-33: Followed the representative procedure. Purification by flash chromatography (8:1 hexanes:EtOAc) yielded alcohol ***(R)*-33** (111.5 mg, 94% yield, 72% ee) as an off-white solid. Alcohol ***(R)*-33**: $[\alpha]_D^{22.8}$ = +28.40° (c = 1.00, $CHCl_3$); R_f 0.15 (5:1 hexanes:EtOAc). Chiral SFC: 250 mm CHIRALPAK AS–H, 20% *i*-PrOH, 3.5 mL/min, λ = 216 nm, 35 °C, nozzle pressure = 100 bar CO_2, t_{R1} (major) = 2.9 min, t_{R2} (minor) = 3.3 min. The spectral data match those obtained for ***rac*-33** as described in Section Gi of the Experimental Procedures.

Part II: Bioinformatics Section

Computational Modelling of the Ketone in the Active Site of the Enzyme (Figure 4)

The 1ZK4.pdb crystal structure was used as the starting template for the structural analysis. The structure was prepared by adding missing atoms such as hydrogens using the Amber 14 force filed as implemented in MOE from the chemical computing group.[33] Computational design methods were used to replace amino acids that differed between the 1ZK4 crystal structure and those from either KRED-404 or P1-B12 using Rosetta.[34] The acetophenone bound in the active site of the 1ZK4 crystal structure was used to guide the placement of ketone **14**. After placement of the ketone the system was minimized using MOE in order to obtain an optimal conformation of the ketone relative to amino acids around the substrate binding pocket and the cofactor.

Sequence Alignments

Supplementary Table 4. KRED P1-B12 and KRED 404 active site sequence mutations relative to WT enzyme.

Position	Wild-Type	P1-B12 †	KRED-404 †
94	A	D	A
96	S	V	S
145	E	F	A
153	L	V	L
190	Y	P	G
202	A	A	F
206	M	W	C
249	Y	W	Y

† Mutation relative to the wild-type enzyme in and around the active site are shown in red.

Supplementary Table 5. KRED 402 and KRED 404 sequence mutations relative to WT enzyme.

Position	KRED-402 †	KRED-404 †
16	T	S
76	I	T
190	C	G
196	V	I

† Mutation around the active site are shown in red.

Supplementary References

[1] Johnson II, D. C. & Widlanski, T. S. A reversible safety-catch method for the hydrogenolysis of *N*-benzyl moieties. *Tetrahedron Lett.* **45**, 8483–8487 (2004).

[2] Weires, N. A., Baker, E. L. & Garg, N. K. Nickel-catalysed Suzuki–Miyaura coupling of amides. *Nat. Chem.* **8**, 75–79 (2016).

[3] Baker, E. L., Yamano, M. M., Zhou, Y., Anthony, S. M. & Garg, N. K. A two-step approach to achieve secondary amide transamidation enabled by nickel catalysis. *Nat. Commun.* **7**, 11154 (2016).

[4] Meng, L., Su, J., Zhang, L., Zhang, Z. & Wang, Z. Direct electrosynthesis of ketones from benzylic methylenes by electrooxidative C–H activation. *Chem. Eur. J.* **19**, 5542–5545 (2013).

[5] Gunasekaran, S. & Venkatasubramanian, N. Oxidation of diphenylmethanol by bromamine T. A kinetic and mechanistic study. *J. Chem. Soc., Perkin Trans. 2*. **0**, 949–953 (1983).

[6] Xin, B., Zhang, Y. & Cheng, K. Phosphine-free cross-coupling reaction of arylboronic acids with carboxylic anhydrides or acyl chlorides in aqueous media. *J. Org. Chem.* **71**, 5725–5731 (2006).

[7] Posternak, A. G., Garlyauskayte, R. Y. & Yagupolskii, L. M. A novel Brønsted acid catalyst for Friedel–Crafts acylation. *Tetrahedron Lett.* **50**, 446–447 (2009).

[8] Kuang, Y. & Wang, Y. Dirhodium(II)-catalyzed cross-coupling reactions of aryl aldehydes with arylboronic acids in water. *Eur. J. Org. Chem.* **6**, 1163–1166 (2014).

[9] Schmink, J. R. & Krska, S. W. Reversed-polarity synthesis of diaryl ketones via Palladium-catalyzed cross-coupling of acylsilanes. *J. Am. Chem. Soc.* **133**, 19574–19577 (2011).

[10] Meng, G., Szostak, R. & Szostak, M. *N*-Methylamino pyrimidyl amides (MAPA): highly reactive, electronically-activated amides in catalytic N–C(O) cleavage. *Org. Lett.* **19**, 4656–4659 (2017).

[11] Yamamoto, T., Ishibashi, A. & Suginome, M. Regioselective synthesis of *o*-benzenediboronic acids via Ir-catalyzed *o*-C–H borylation directed by a pyrazolylaniline-modified boronyl group. *Org. Lett.* **19**, 886–889 (2017).

[12] Collet, F., Song, B., Rudolphi, F. & Goossen, L. J. Development of decarboxylative coupling processes for the synthesis of azomethines and ketones. *Eur. J. Org. Chem.* **32**, 6486–6501 (2011).

[13] Halima, T. B., Zhang, W., Yalaoui, I., Hong, X., Yang, Y.-F., Houk, K. N. & Newman, S. G. Palladium-catalyzed Suzuki–Miyaura coupling of aryl esters. *J. Am. Chem. Soc.* **139**, 1311–1318 (2017).

[14] Huan, P.-Q. & Chena, H. Ni-Catalyzed cross-coupling reactions of *N*-acylpyrrole-type amides with organoboron reagents. *Chem. Commun*. **53**, 12584–12587 (2017).

[15] Yamamoto, K., Tsurumi, K., Sakurai, F., Kondo, K. & Aoyama, T. Development of asymmetric nickel-catalyzed arylation of aromatic aldehydes with arylboron reagents. *Synthesis* **22**, 3585–3590 (2008).

[16] Watanabe, M., Kuwahara, S., Harada, N., Koizumi, M. & Ohkuma, T. Enantioresolution by the chiral phthalic acid method: absolute configurations of (2-methylphenyl)phenylmethanol and related compounds. *Tetrahedron: Asymmetry* **10**, 2075–2078 (1999).

[17] Lee, T., Wilson, T. W., Berg, R., Ryberg, P. & Hartwig, J. F. Rhodium-catalyzed enantioselective silylation of arene C–H bonds: desymmetrization of diarylmethanols. *J. Am. Chem. Soc.* **137**, 6742–6745 (2015).

[18] Fontes, M., Verdaguer, X., Solà, L., Pericàs, M. A. & Riera, A. 2-Piperidino-1,1,2-triphenylethanol: a highly effective catalyst for the enantioselective arylation of aldehydes. *J. Org. Chem.* **69**, 2532–2543 (2004).

[19] Chang, S.-J., Zhou, S. & Gau, H.-M. Enantioselective addition of ArTi(O*i*-Pr)$_3$ to aldehydes catalyzed by a titanium complex of an *N*-sulfonylated amino alcohol. *RSC Adv.* **5**, 9368–9373 (2015).
[20] Lee, C.-T. & Lipshutz, B. H. Nonracemic diarymethanols from CuH-catalyzed hydrosilylation of diaryl ketones. *Org. Lett.* **10**, 4187–4190 (2008).
[21] Naito, J., Kosaka, M., Sugito, T., Watanabe, M., Harada, N. & Pirkle, W. H. Enantioresolution of fluorinated diphenylmethanols and determination of their absolute configurations by X-ray crystallographic and 1H NMR anisotropy methods. *Chirality* **16**, 22–35 (2004).
[22] Tanpure, R. P., Harkrider, A. R., Strecker, T. E., Hamel, E., Trawick, M. L. & Pinney, K. G. Application of the McMurry coupling reaction in the synthesis of tri- and tetra-arylethylene analogues as potential cancer chemotherapeutic agents. *Bioorg. Med. Chem.* **17**, 6993–7001 (2009).
[23] Zhou, S., Wu, K.-H., Chen, C.-A. & Gau, H.-M. Highly enantioselective arylation of aldehydes and ketones using AlArEt$_2$(THF) as aryl sources. *J. Org. Chem.* **74**, 3500–3505 (2009).
[24] Jagt, R. B. C., Toullec, P. Y., de Vries, J. G., Feringa, B. L. & Minnaard, A. J. Rhodium/phosphoramidite-catalyzed asymmetric arylation of aldehydes with arylboronic acids. *Org. Biomol. Chem.* **4**, 773–775 (2006).
[25] Moon, S.-Y., Jung, S.-H., Kim, U. B. & Kim, W.-S. Synthesis of Ketones via organolithium addition to acid chlorides using continuous flow chemistry. *RSC Adv.* **5**, 79385–79390 (2015).
[26] Ziegler, D. S., Karaghiosoff, K. & Knochel, P. Generation of aryl and heteroaryl magnesium reagents in toluene by Br/Mg or Cl/Mg exchange. *Angew. Chem., Int. Ed.* **57**, 6701–6704 (2018).
[27] Yamamoto, T., Ohta, T. & Ito, Y. Palladium-catalyzed addition of arylboronic acids to aldehydes. *Org. Lett.* **7**, 4153–4155 (2005).
[28] Jia, X., Fang, L., Lin, A., Pan, Y. & Zhu, C. Highly efficient and facile aryl transfer to aldehydes using ArB(OH)$_2$–GaMe$_3$. *Synlett* **3**, 495–499 (2009).
[29] Kuriyama, M., Shimazawa, R., Enomoto, T., Shirai, T. Palladium-catalyzed 1,2-addition of potassium aryl- and alkyenyltrifluoroborates to aldehydes using thioether–imidazolinium carbene ligands. *J. Org. Chem.* **73**, 6939–6942 (2008).
[30] Schmidt, F., Stemmler, R. T., Rudolph, J. & Bolm, C. Catalytic asymmetric approaches towards enantiomerically enriched diarymethanols and diarylmethylamines. *Chem. Soc. Rev.* **35**, 454–470 (2006).
[31] Braga, A. L., Paixão, M. W., Westermann, B., Schneider, P. H. & Wessjohann, L. A. Acceleration of arylzinc formation and its enantioselective addition to aldehydes by microwave irradiation and aziridine-2-methanol catalysts. *J. Org. Chem.* **73**, 2879–2882 (2008).
[32] Greene, M. A., Yonova, I. M., Williams, F. J. & Jarvo, E. R. Traceless directing group for stereospecific nickel-catalyzed alkyl–alkyl cross-coupling reactions. *Org. Lett.* **14**, 4293–4296 (2012).
[33] *Molecular Operating Environment* (MOE), 2013.08; Chemical Computing Group ULC, 1010 Sherbooke St. West, Suite #910, Montreal, QC, Canada, H3A 2R7 (2018).
[34] Kuhlman, B., Dantas, G., Ireton, G. C., Varani, G., Stoddard, B. L.& Baker, D. Design of a novel globular protein fold with atomic-level accuracy. *Science* **302**, 1364–1368 (2003).

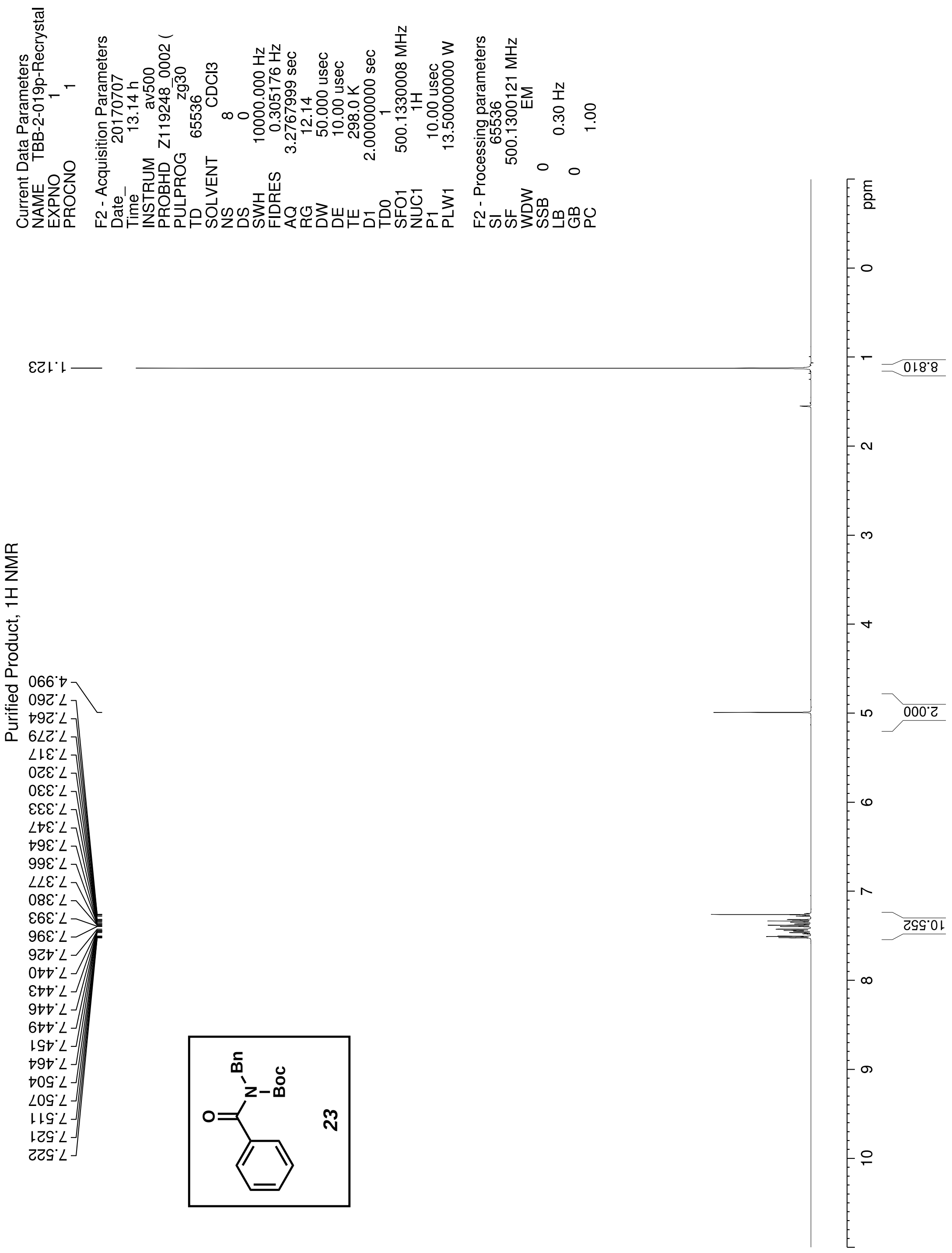

Supplementary Figure 2. ^{1}H NMR spectrum for compound **23**.

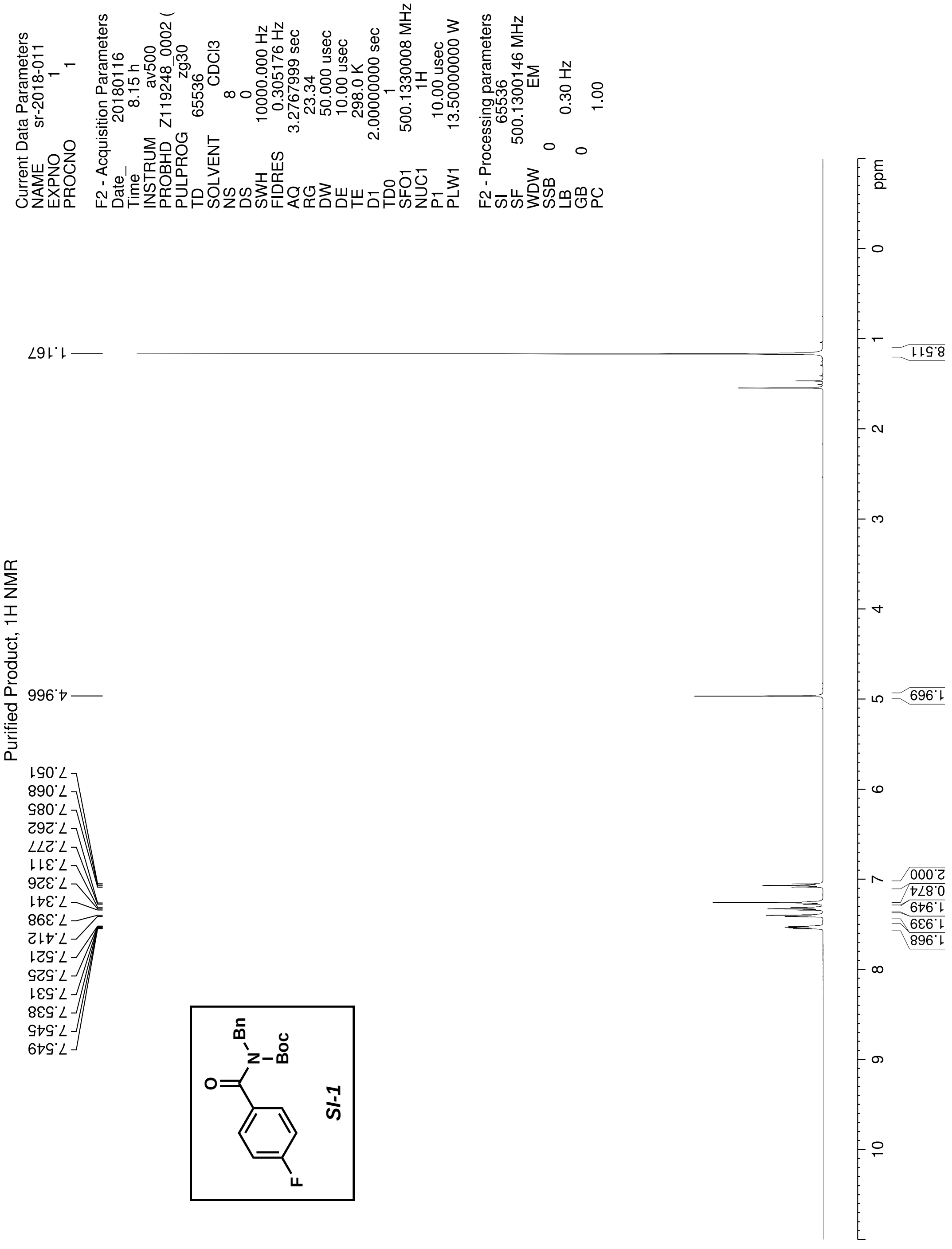

Supplementary Figure 3. ^{1}H NMR spectrum for compound ***SI-1***.

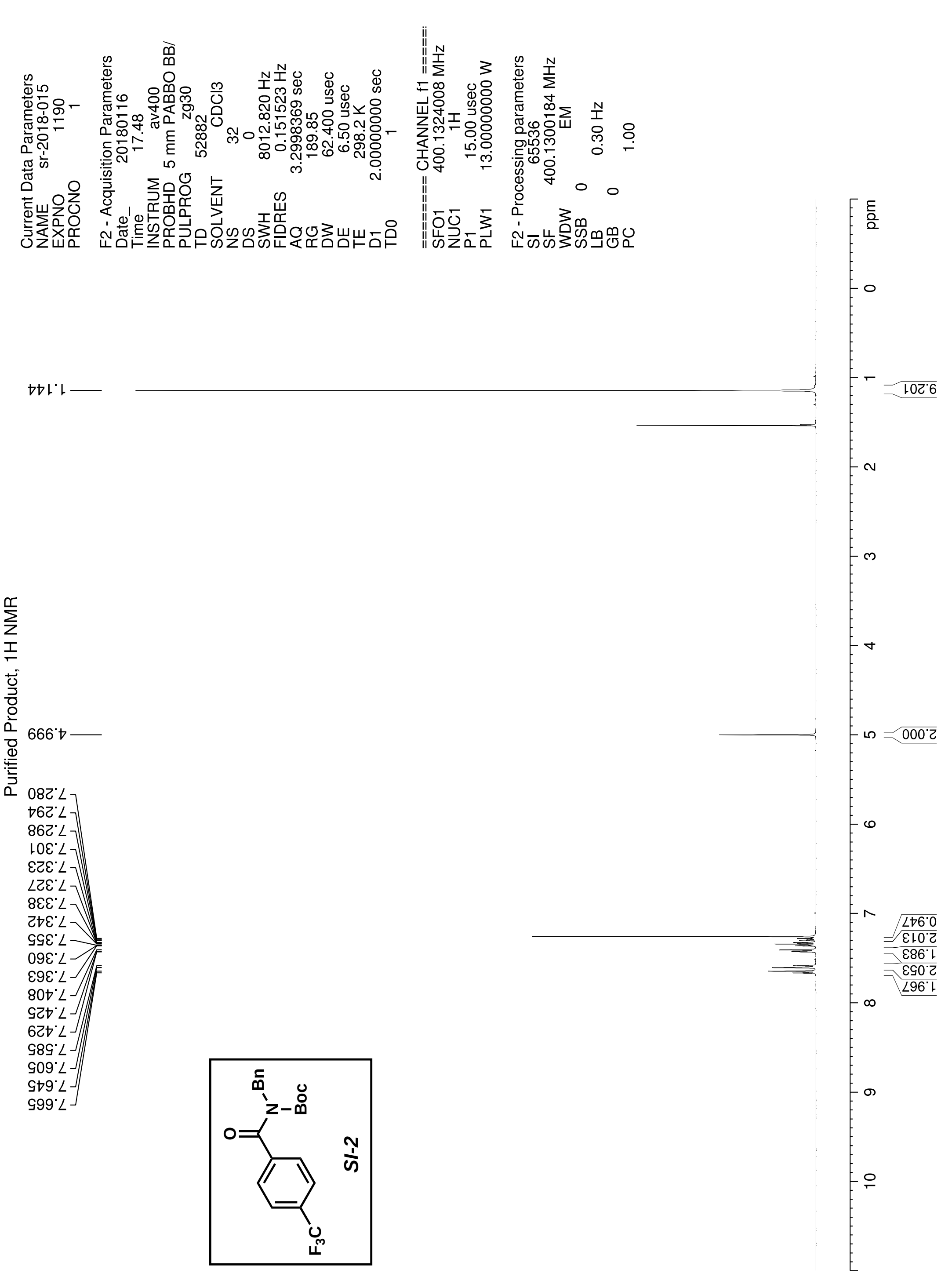

Supplementary Figure 4. ^{1}H NMR spectrum for compound ***SI-2***.

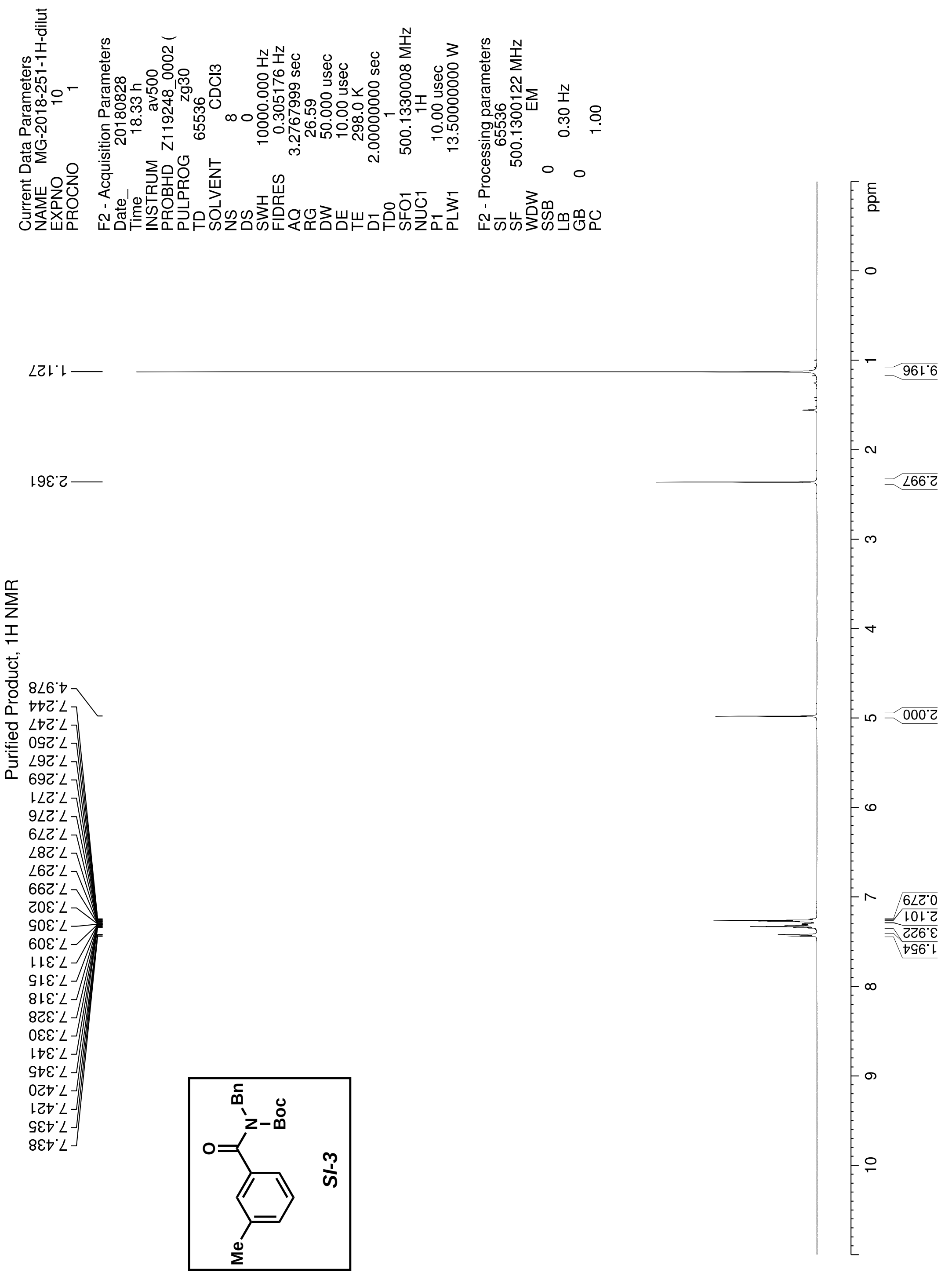

Supplementary Figure 5. ^{1}H NMR spectrum for compound ***SI-3***.

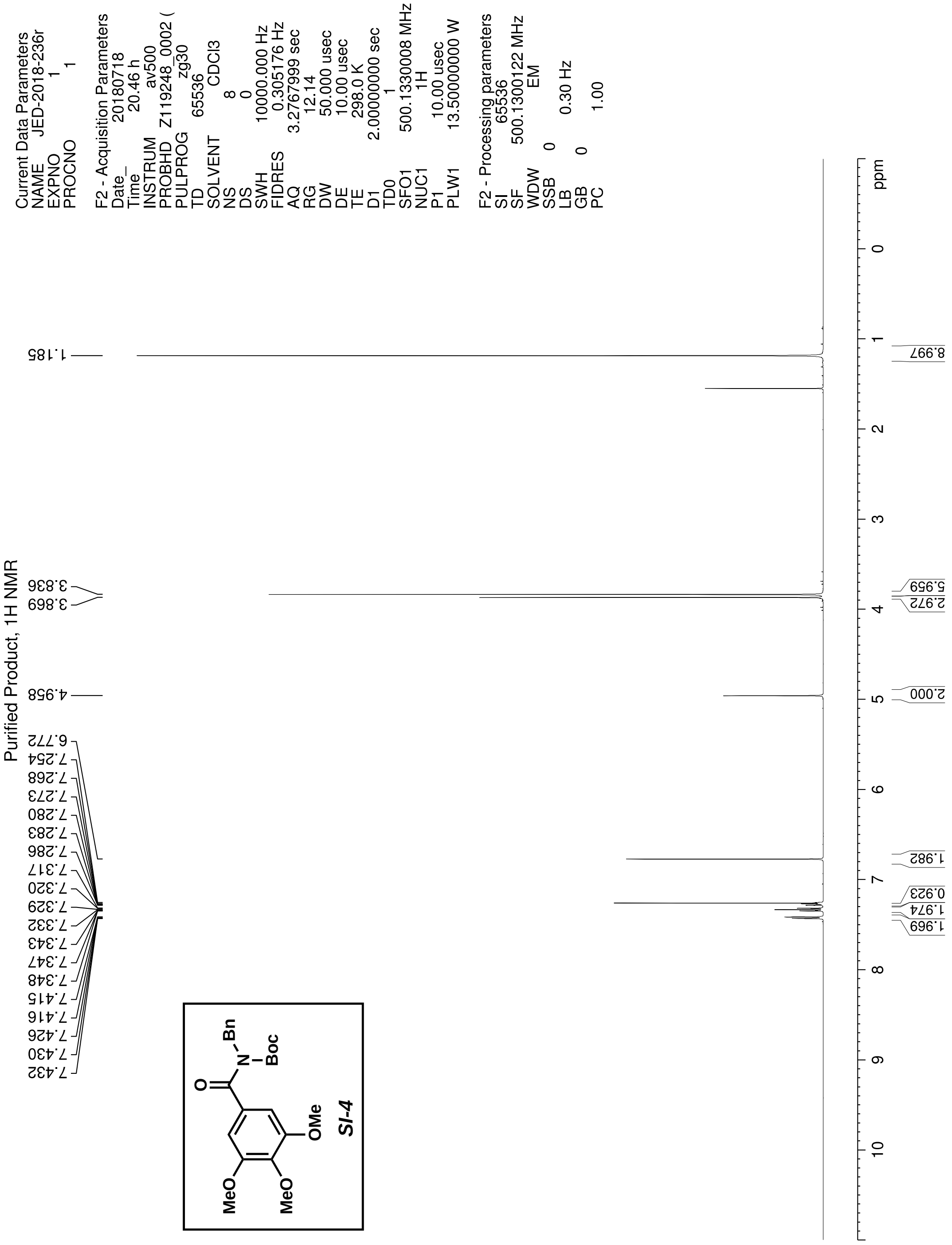

Supplementary Figure 6. ^{1}H NMR spectrum for compound ***SI-4***.

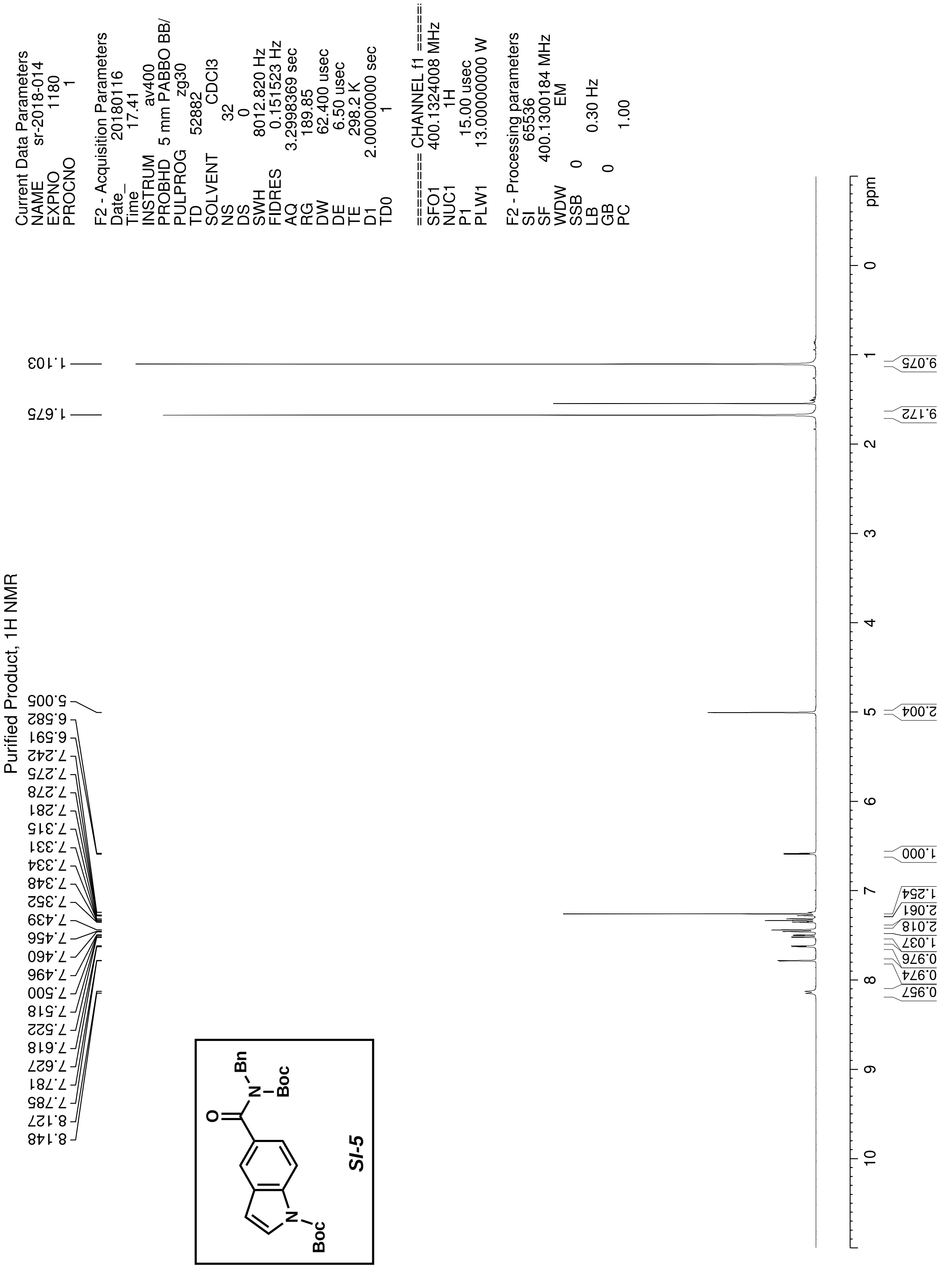

Supplementary Figure 7. ^{1}H NMR spectrum for compound ***SI-5***.

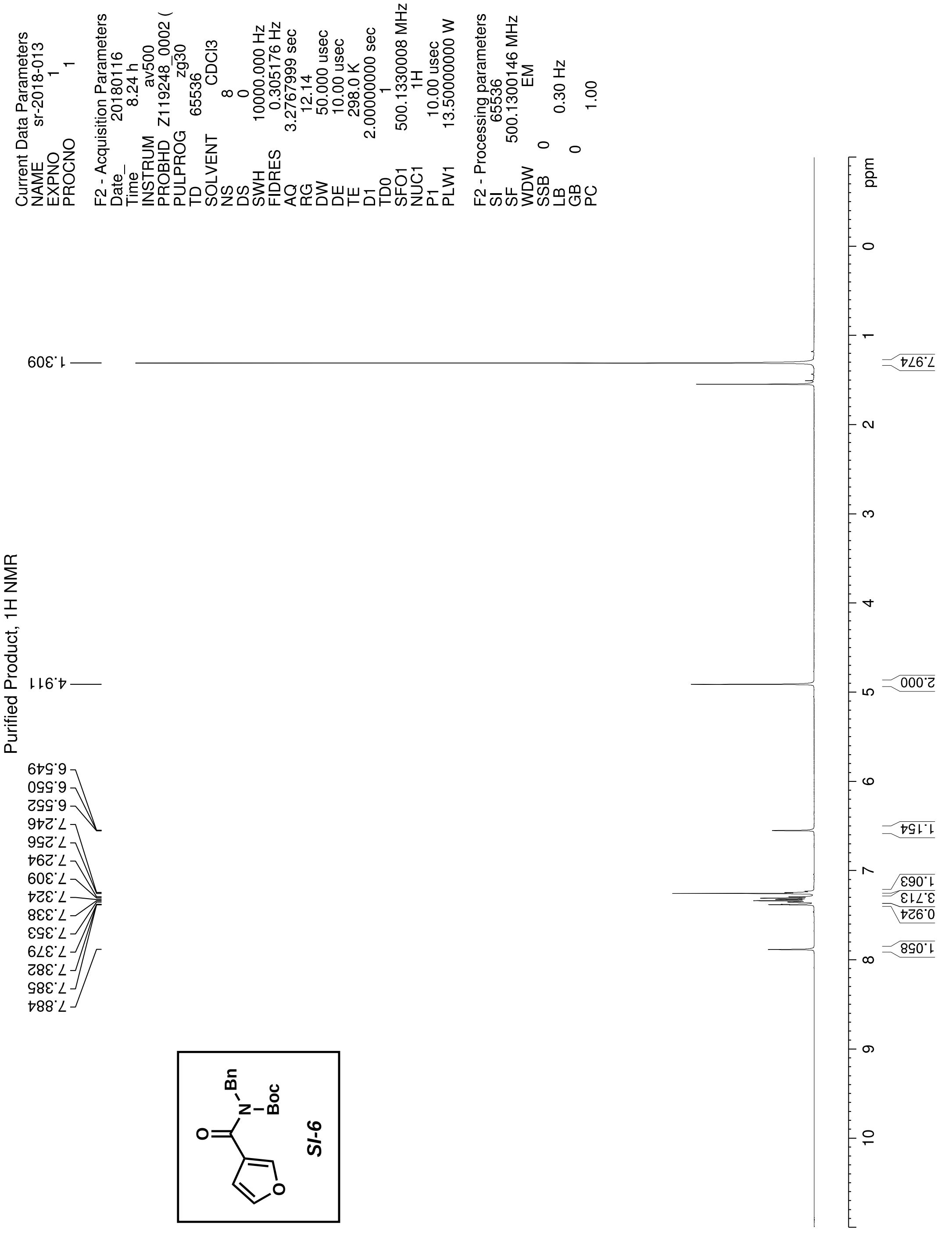

Supplementary Figure 8. ^{1}H NMR spectrum for compound ***SI-6***.

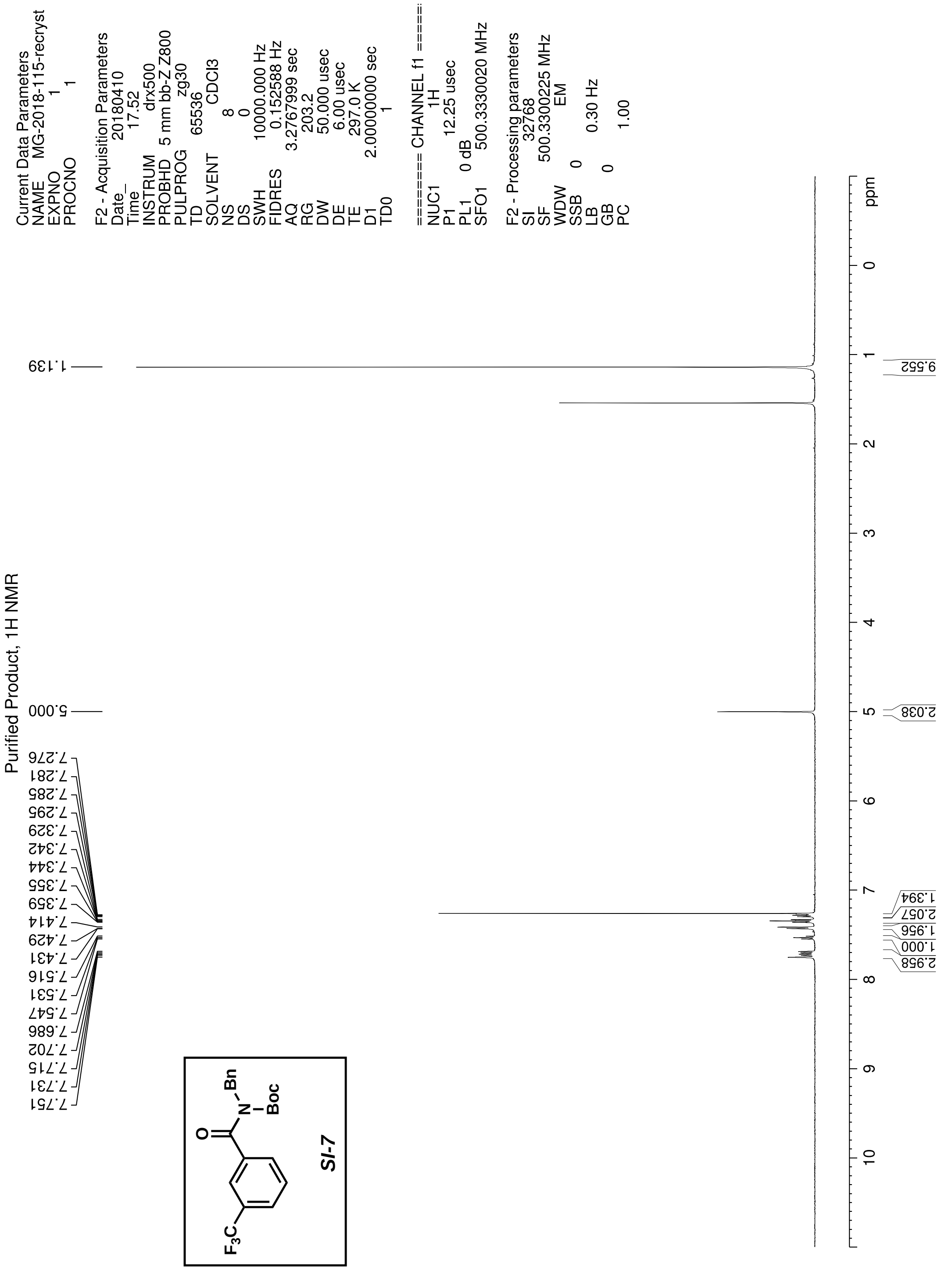

Supplementary Figure 9. ^{1}H NMR spectrum for compound ***SI-7***.

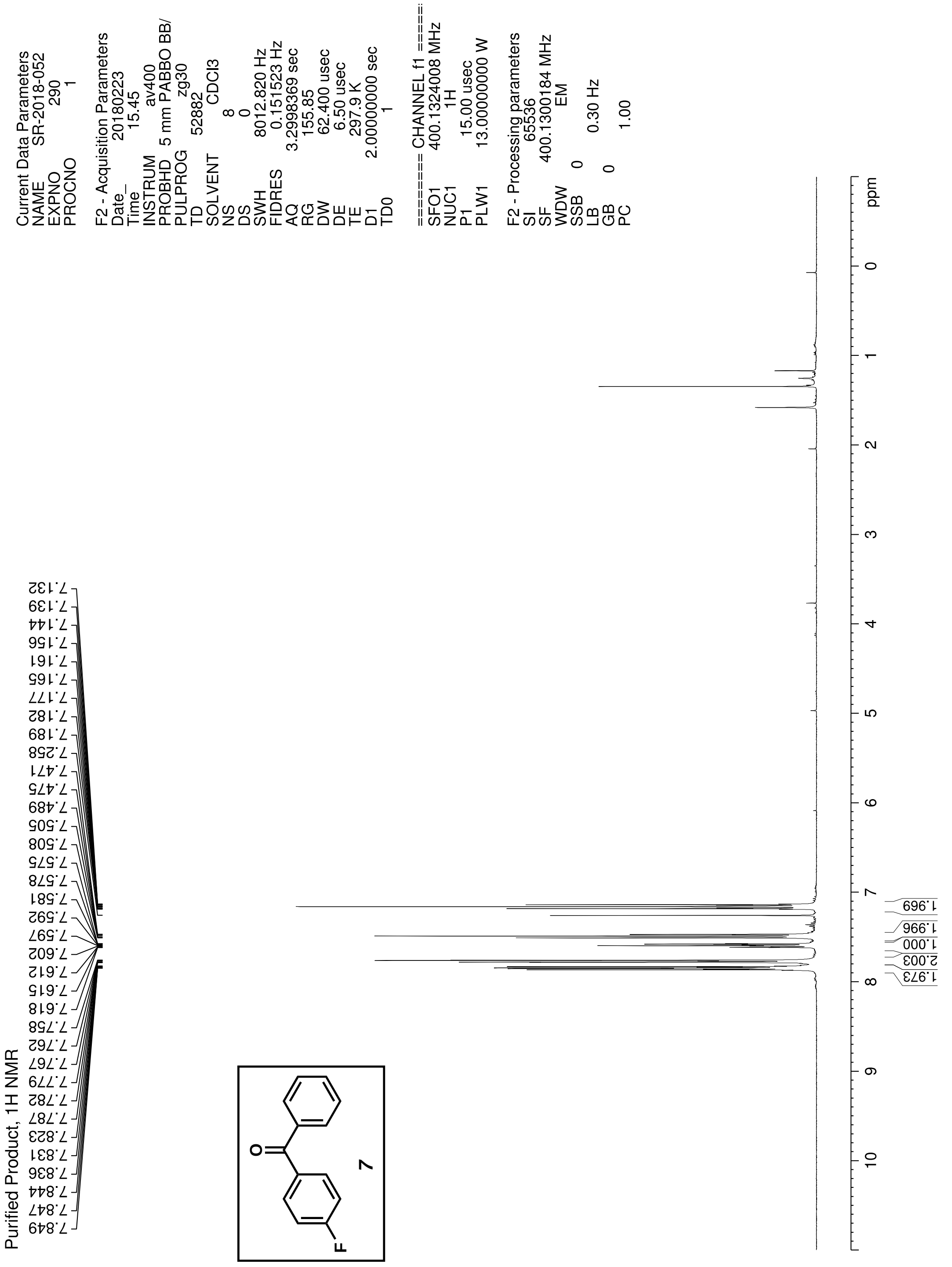

Supplementary Figure 10. ^{1}H NMR spectrum for compound ***7***.

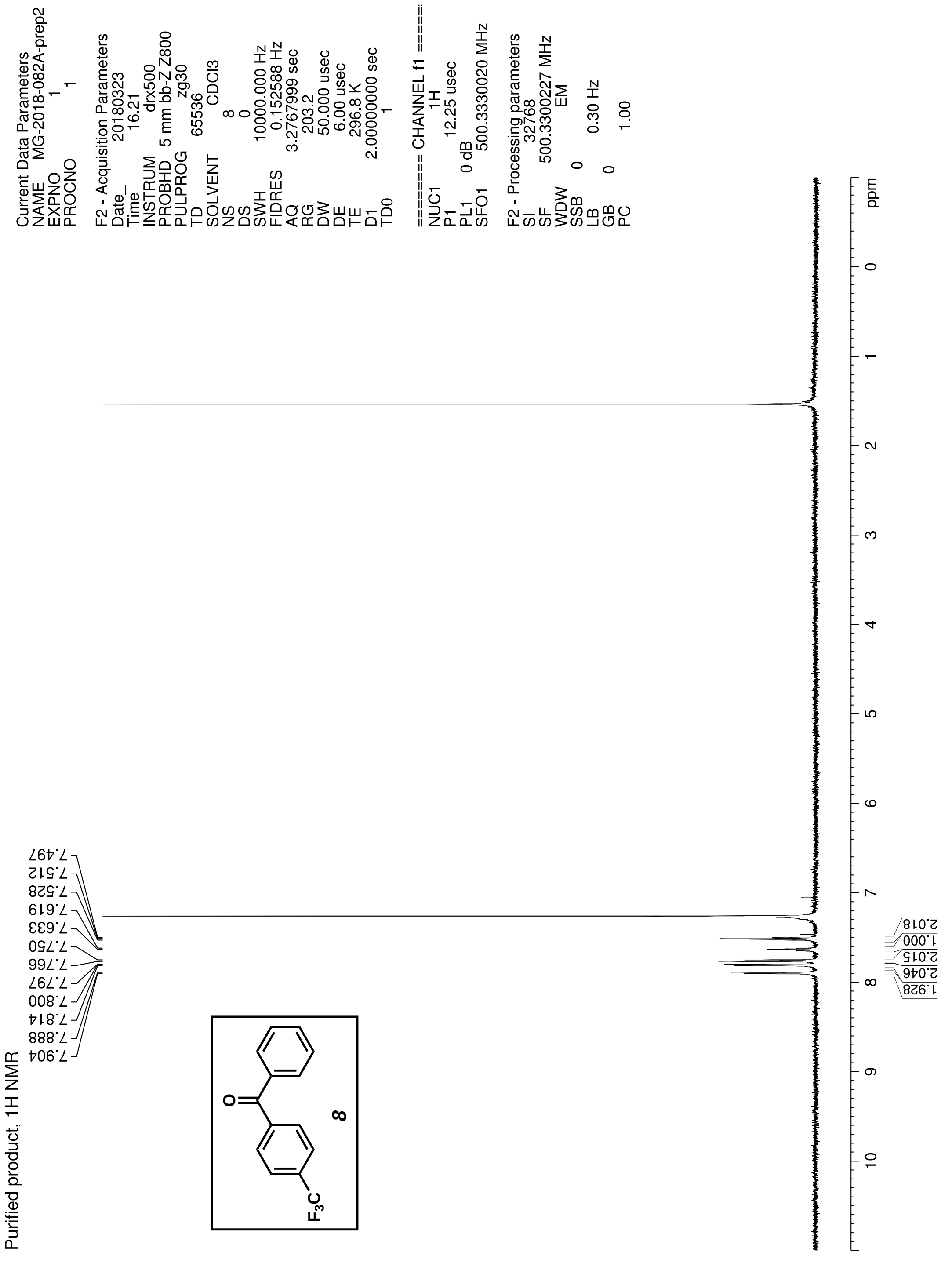

Supplementary Figure 11. ^{1}H NMR spectrum for compound ***8***.

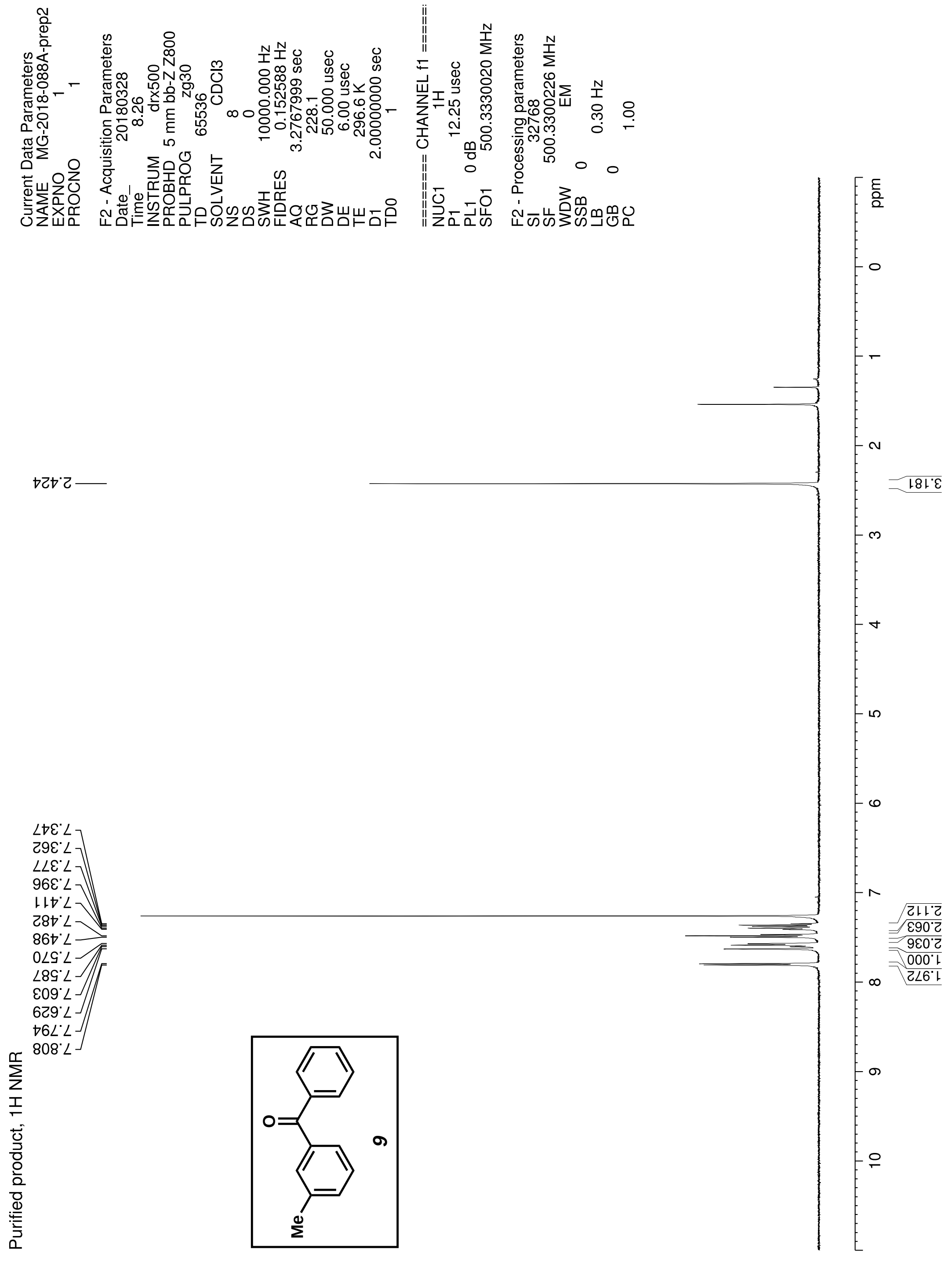

Supplementary Figure 12. ^{1}H NMR spectrum for compound ***9***.

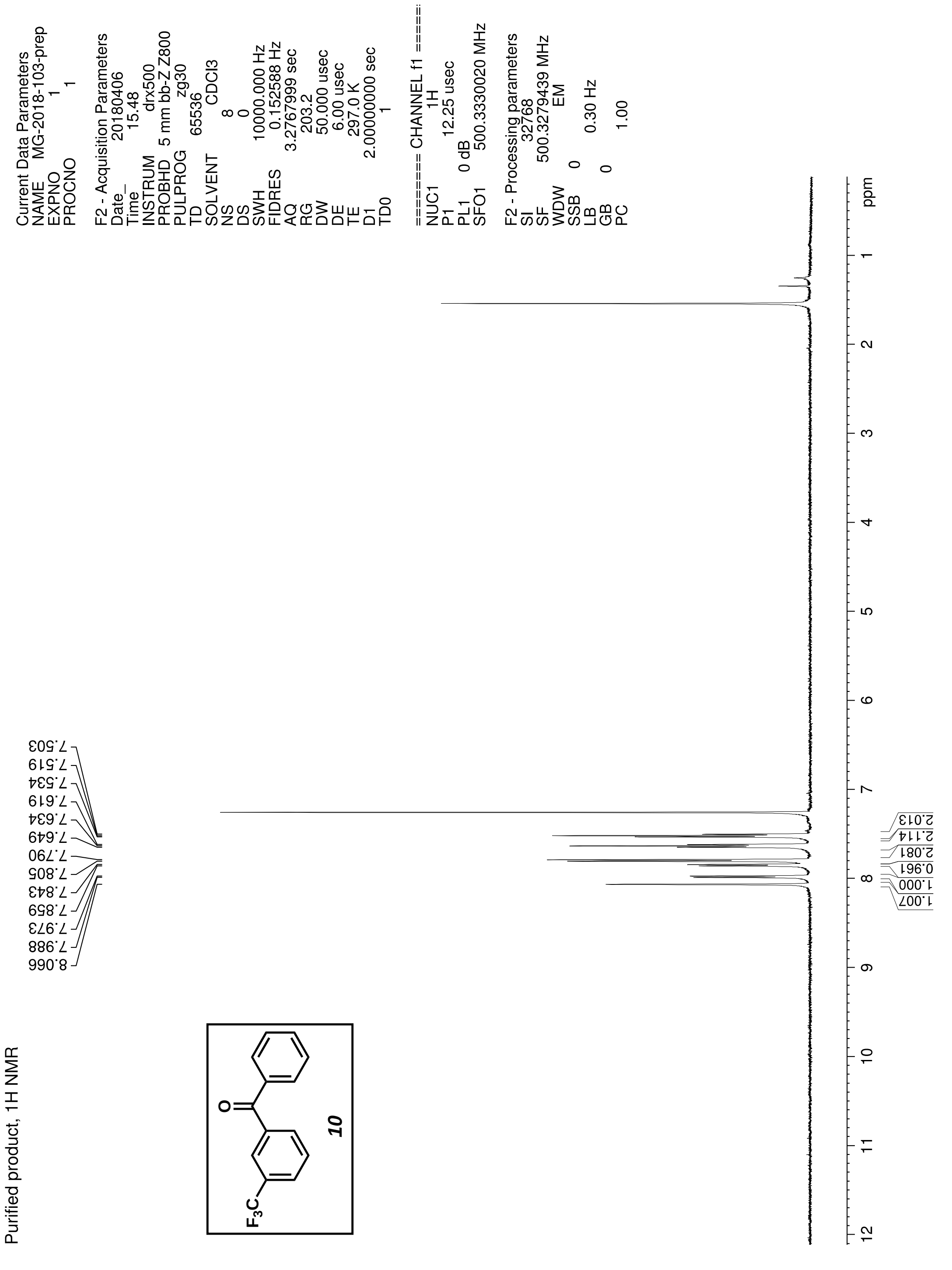

Supplementary Figure 13. ^{1}H NMR spectrum for compound ***10***.

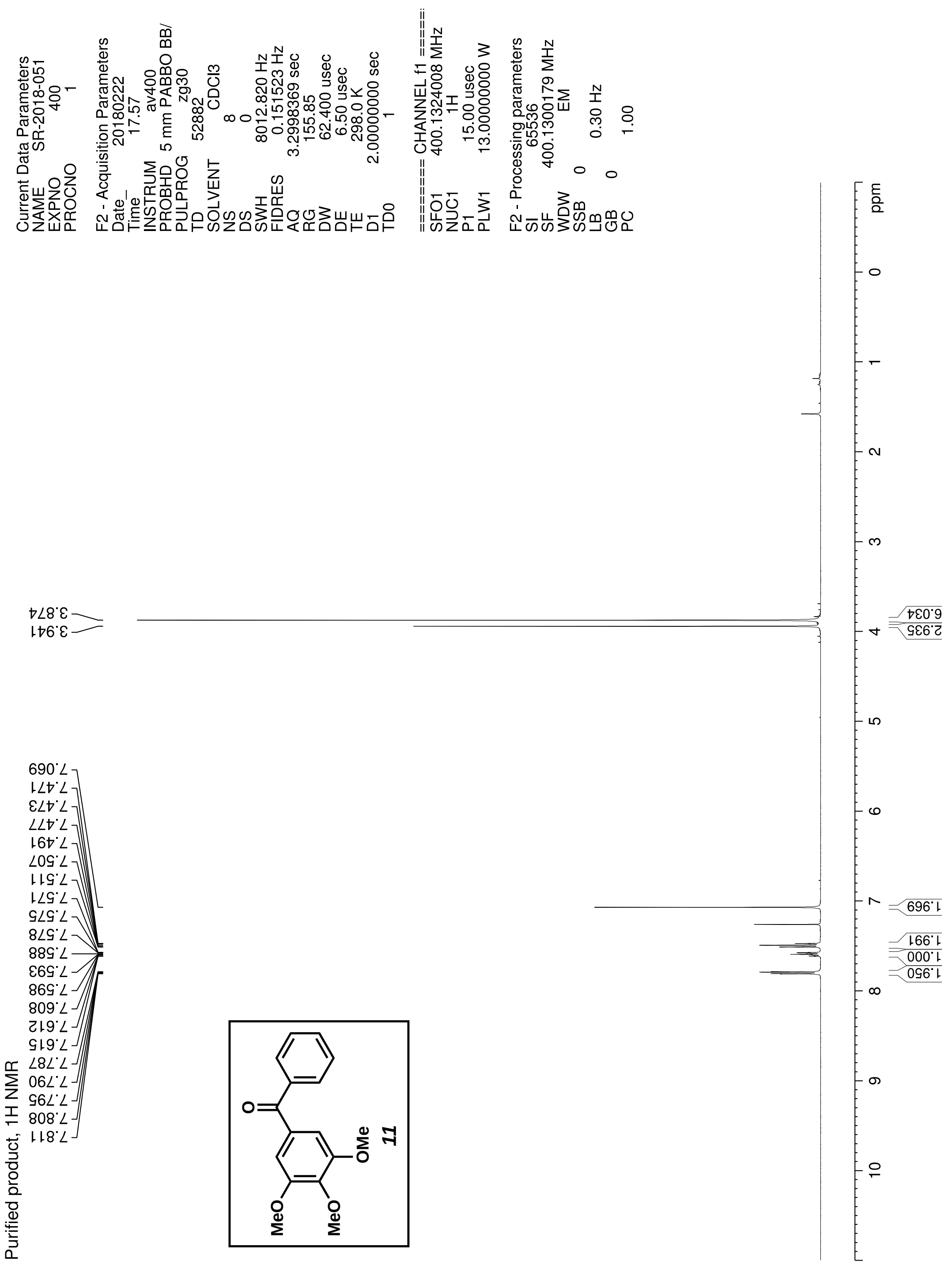

Supplementary Figure 14. ^{1}H NMR spectrum for compound ***11***.

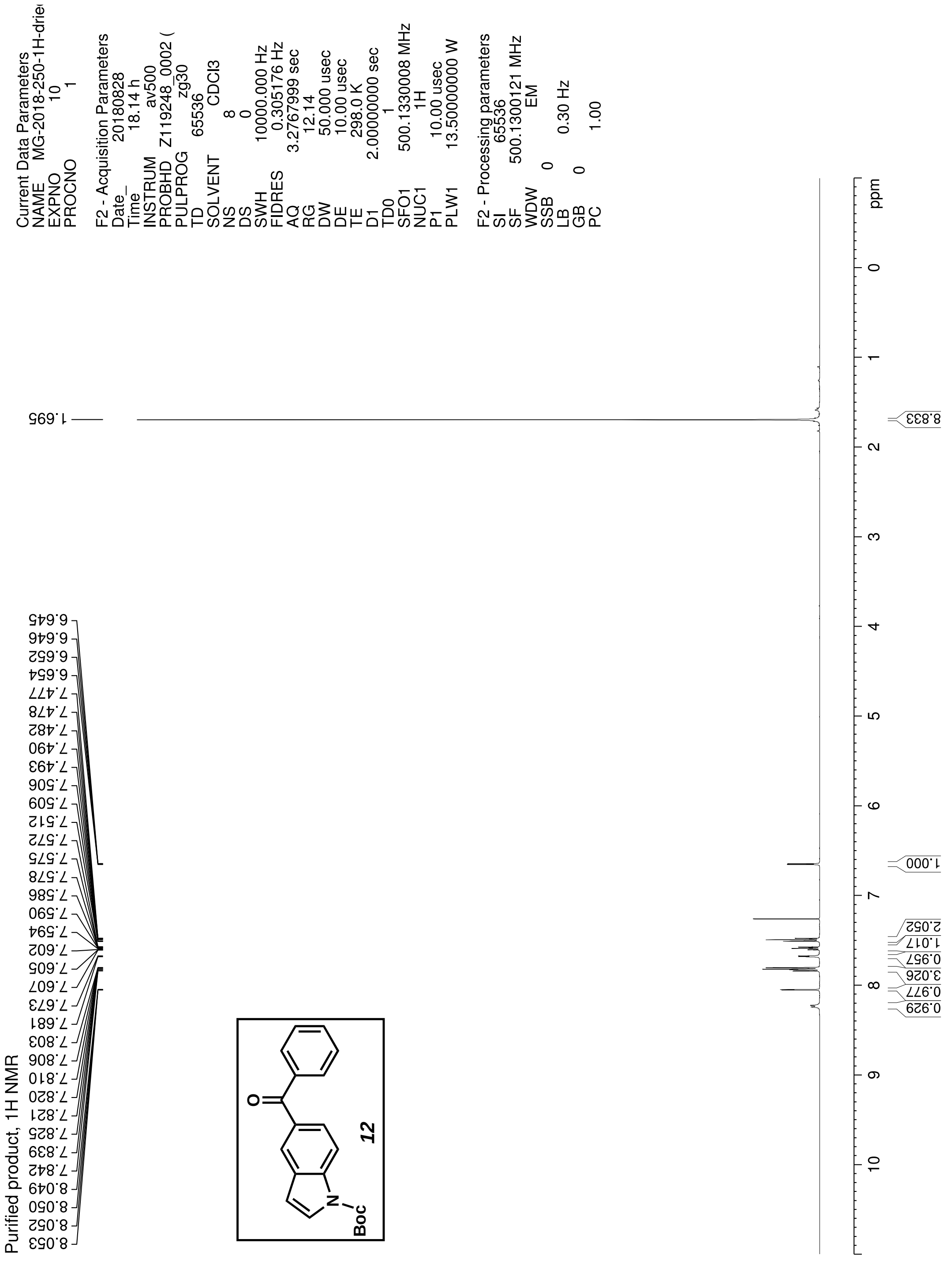

Supplementary Figure 15. ^{1}H NMR spectrum for compound ***12***.

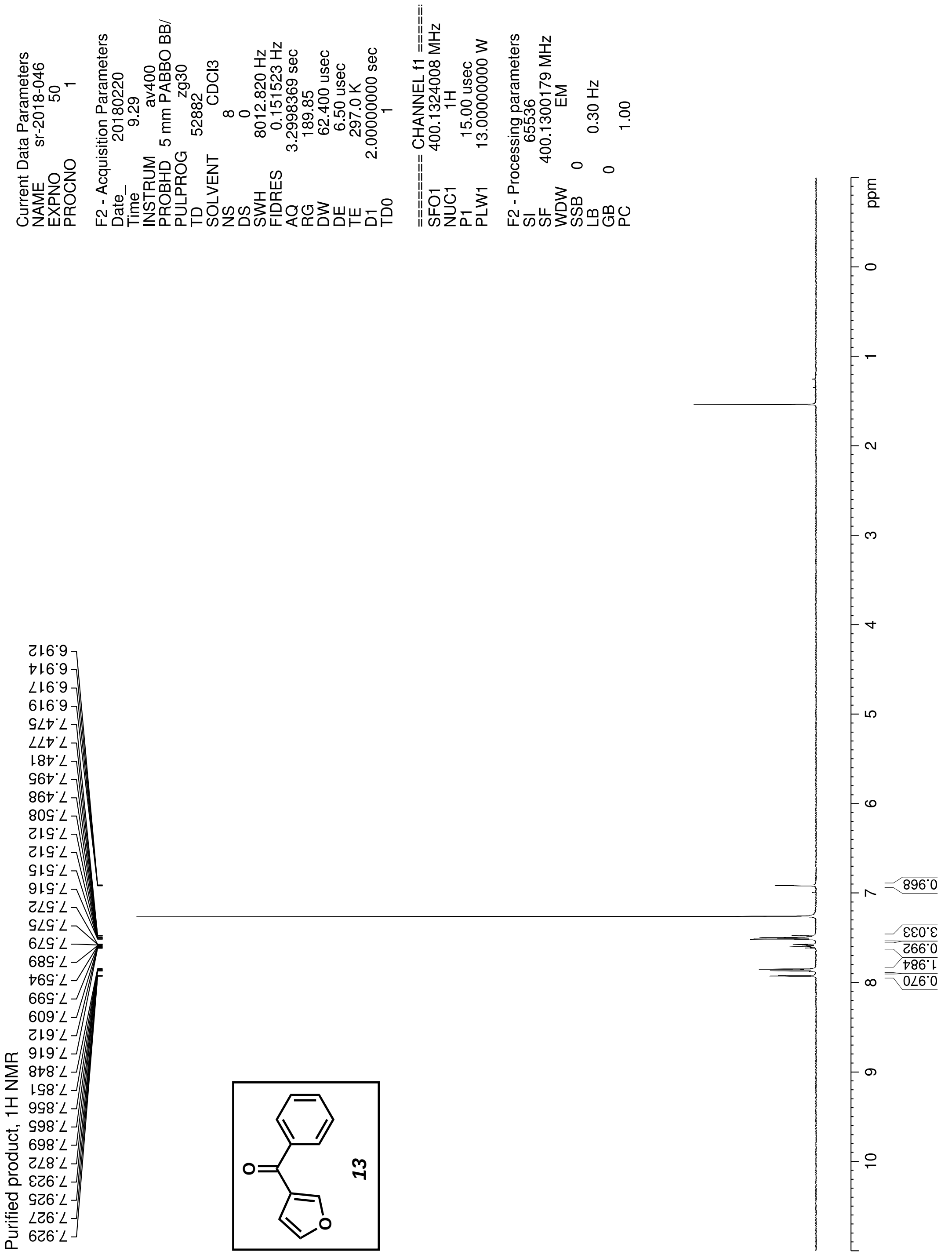

Supplementary Figure 16. ^{1}H NMR spectrum for compound ***13***.

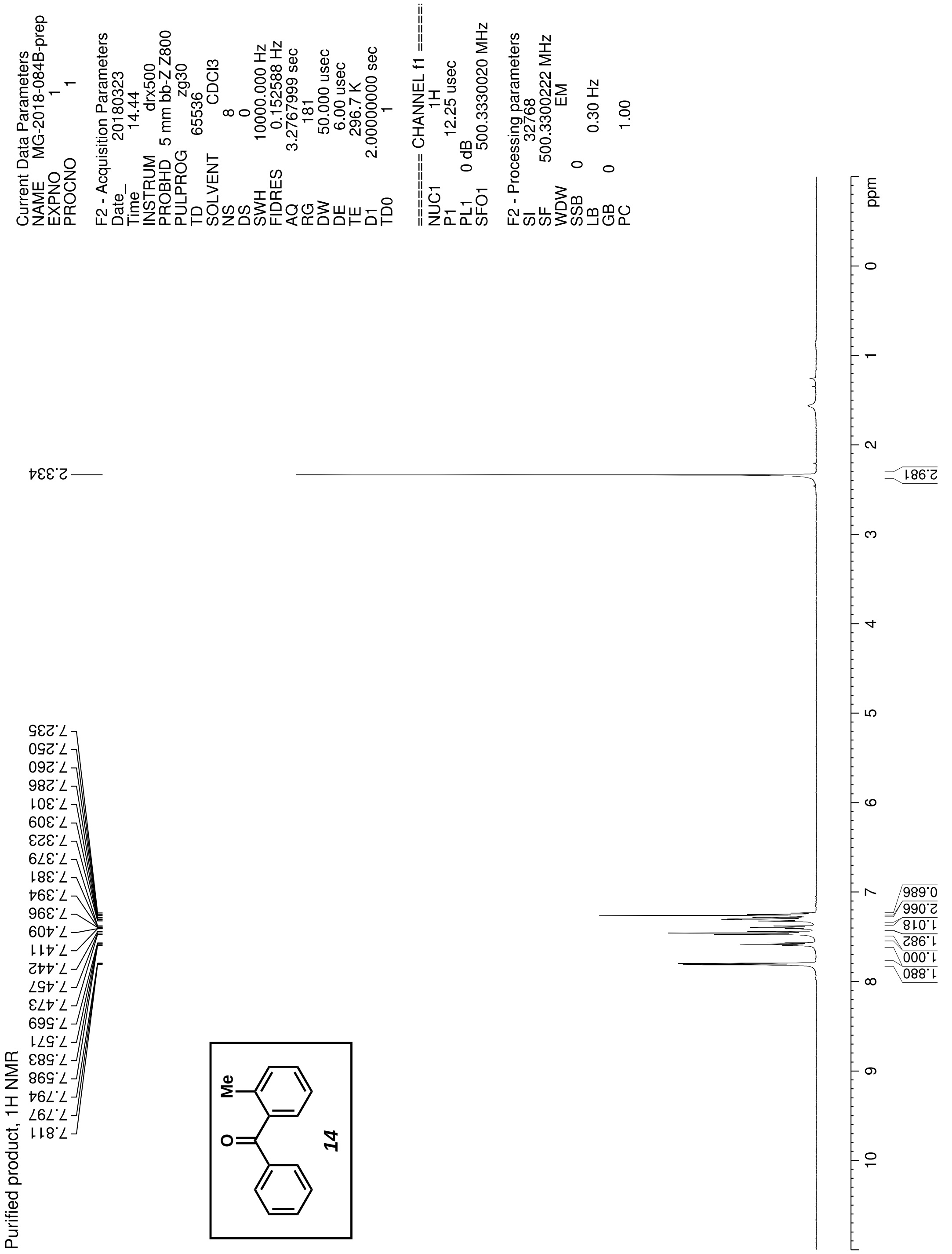

Supplementary Figure 17. ^{1}H NMR spectrum for compound ***14***.

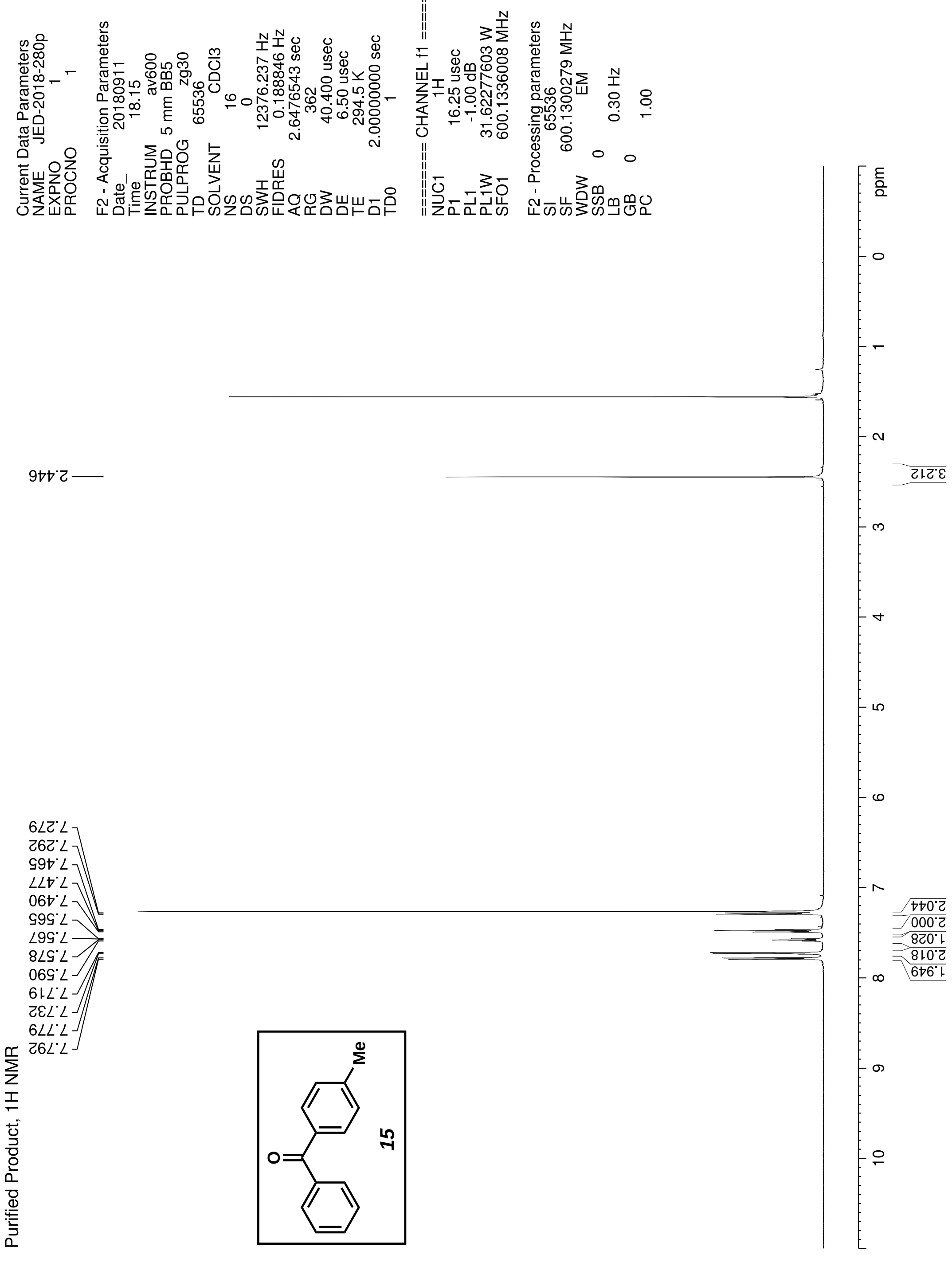

Supplementary Figure 18. ^{1}H NMR spectrum for compound ***15***.

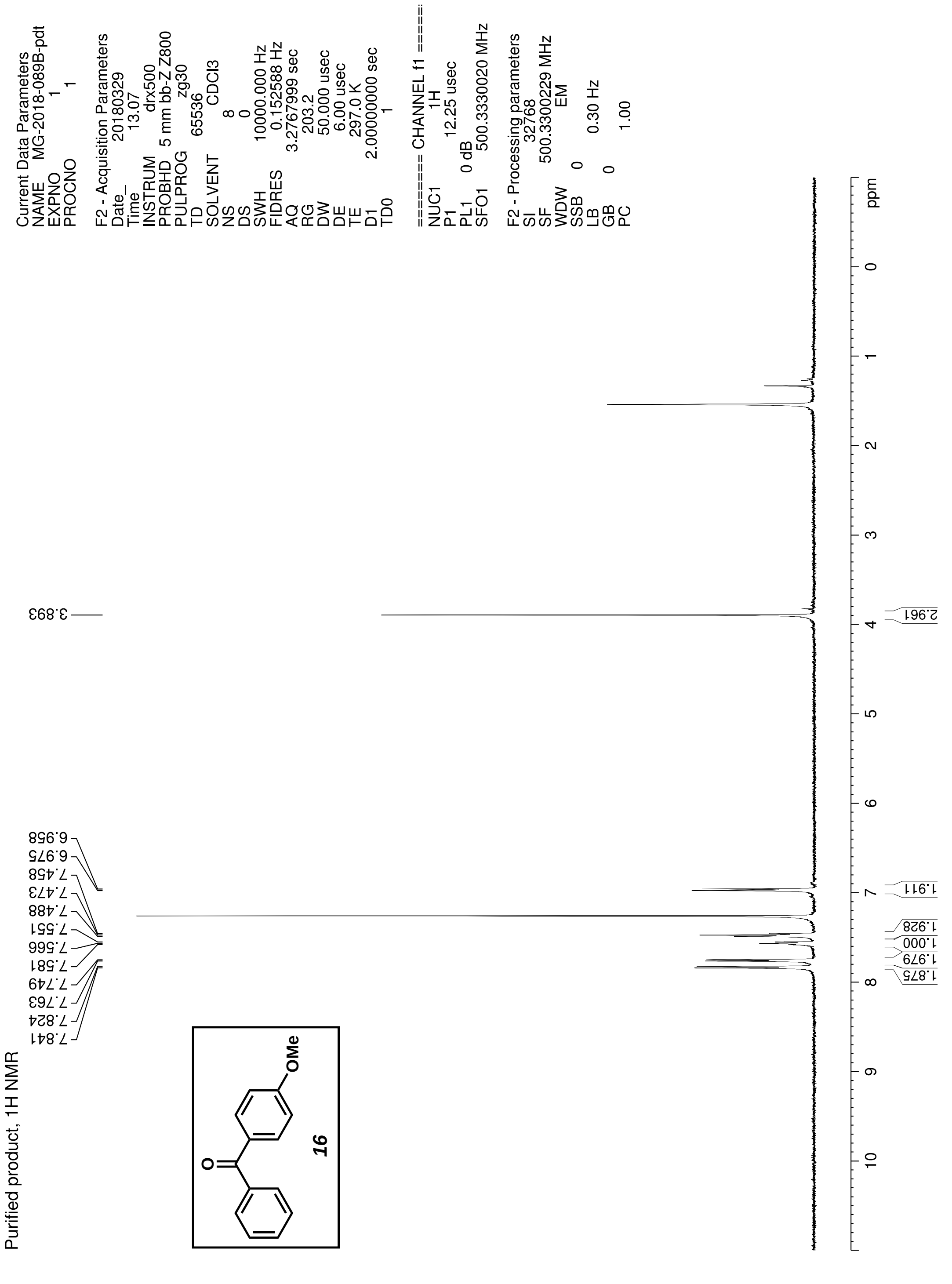

Supplementary Figure 19. ^{1}H NMR spectrum for compound ***16***.

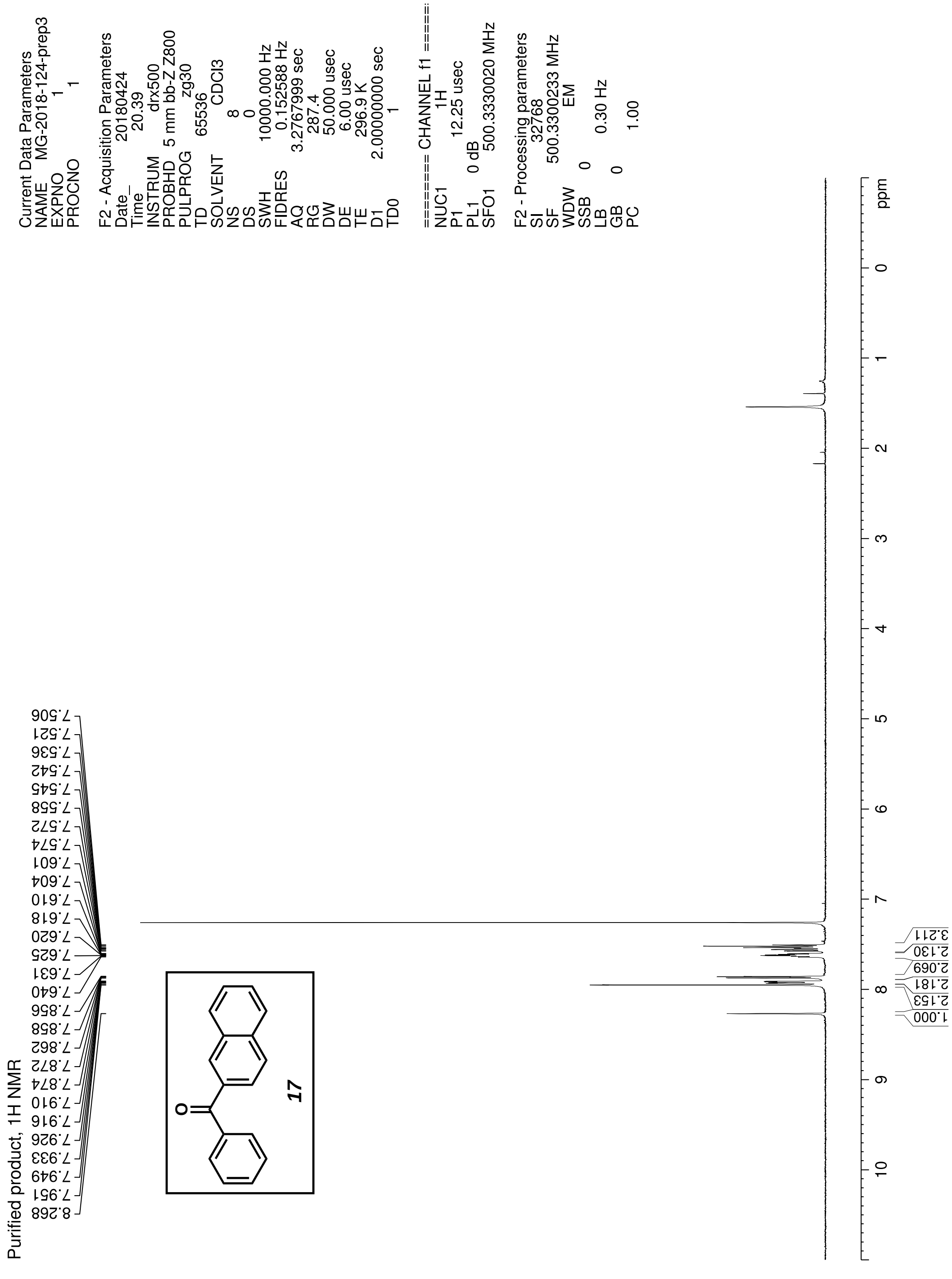

Supplementary Figure 20. ^{1}H NMR spectrum for compound ***17***.

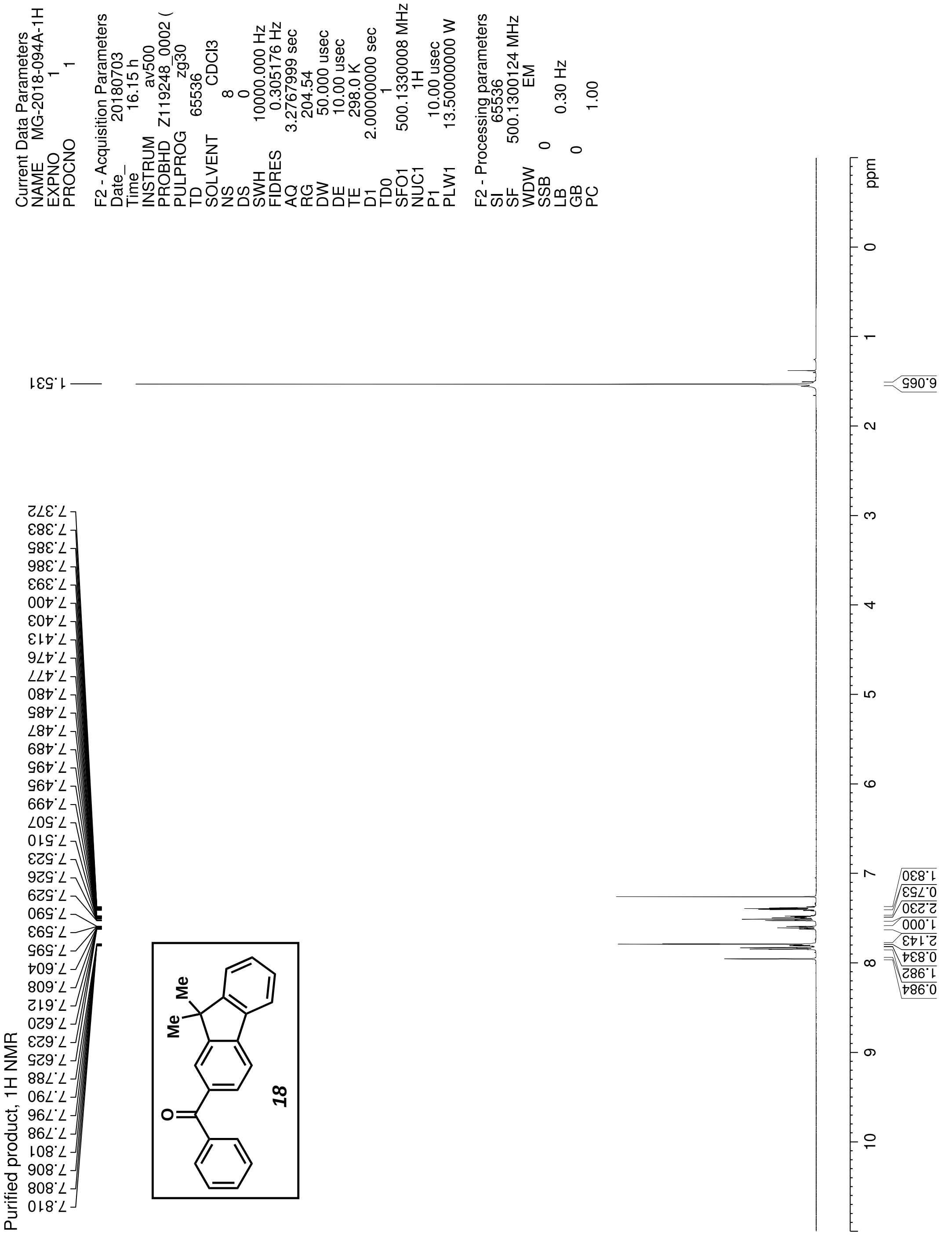

Supplementary Figure 21. ^{1}H NMR spectrum for compound ***18***.

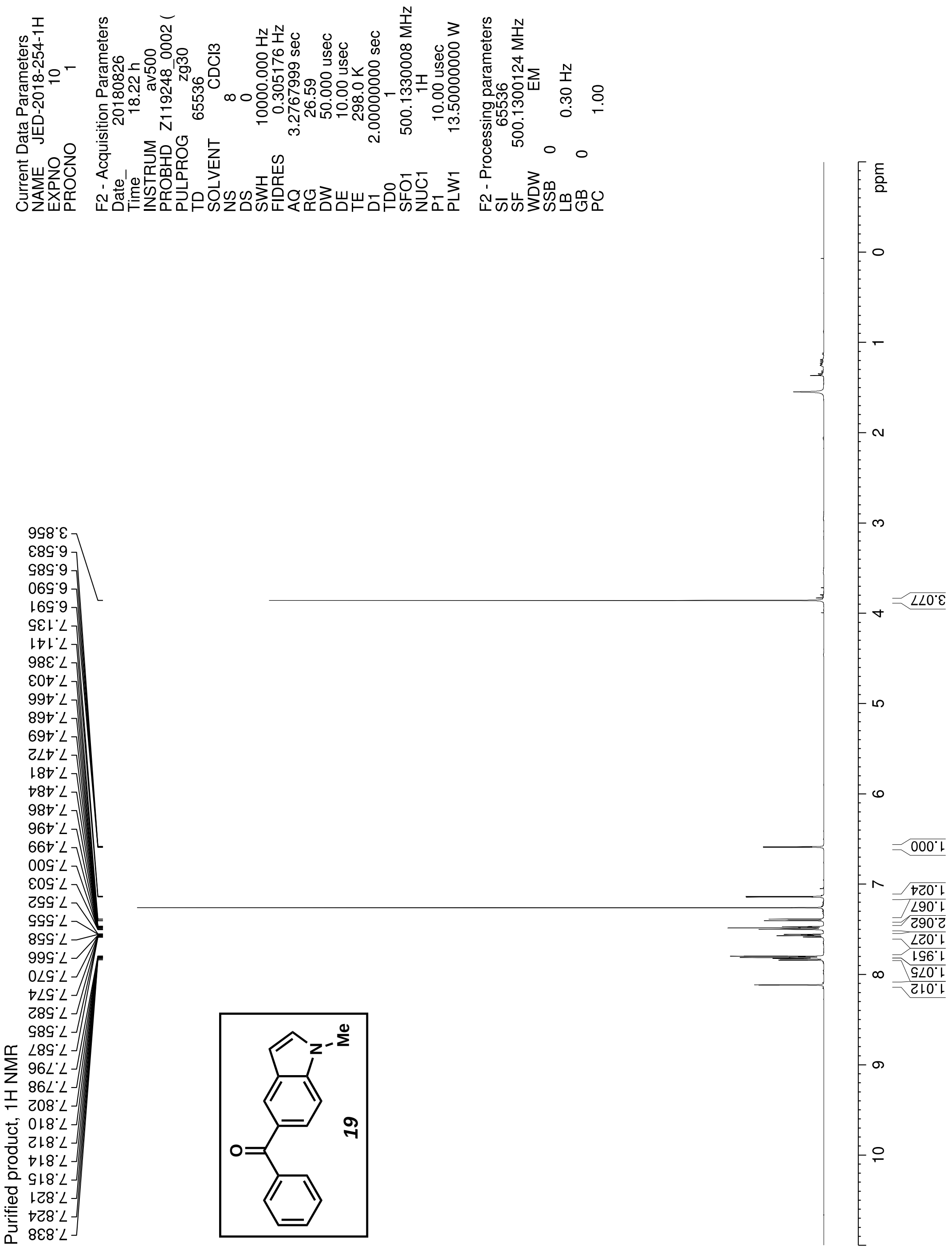

Supplementary Figure 22. ^{1}H NMR spectrum for compound ***19***.

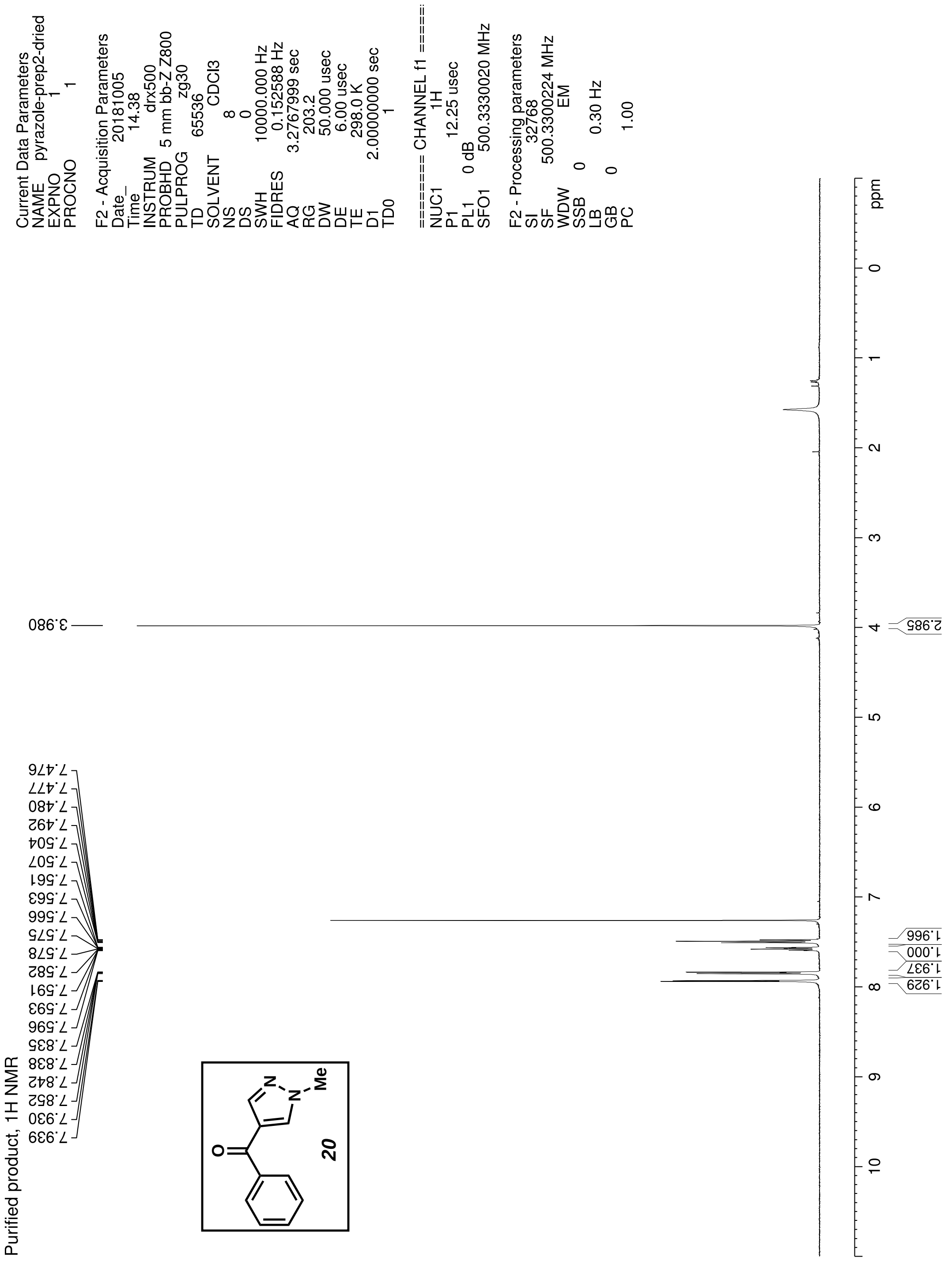

Supplementary Figure 23. ^{1}H NMR spectrum for compound ***20***.

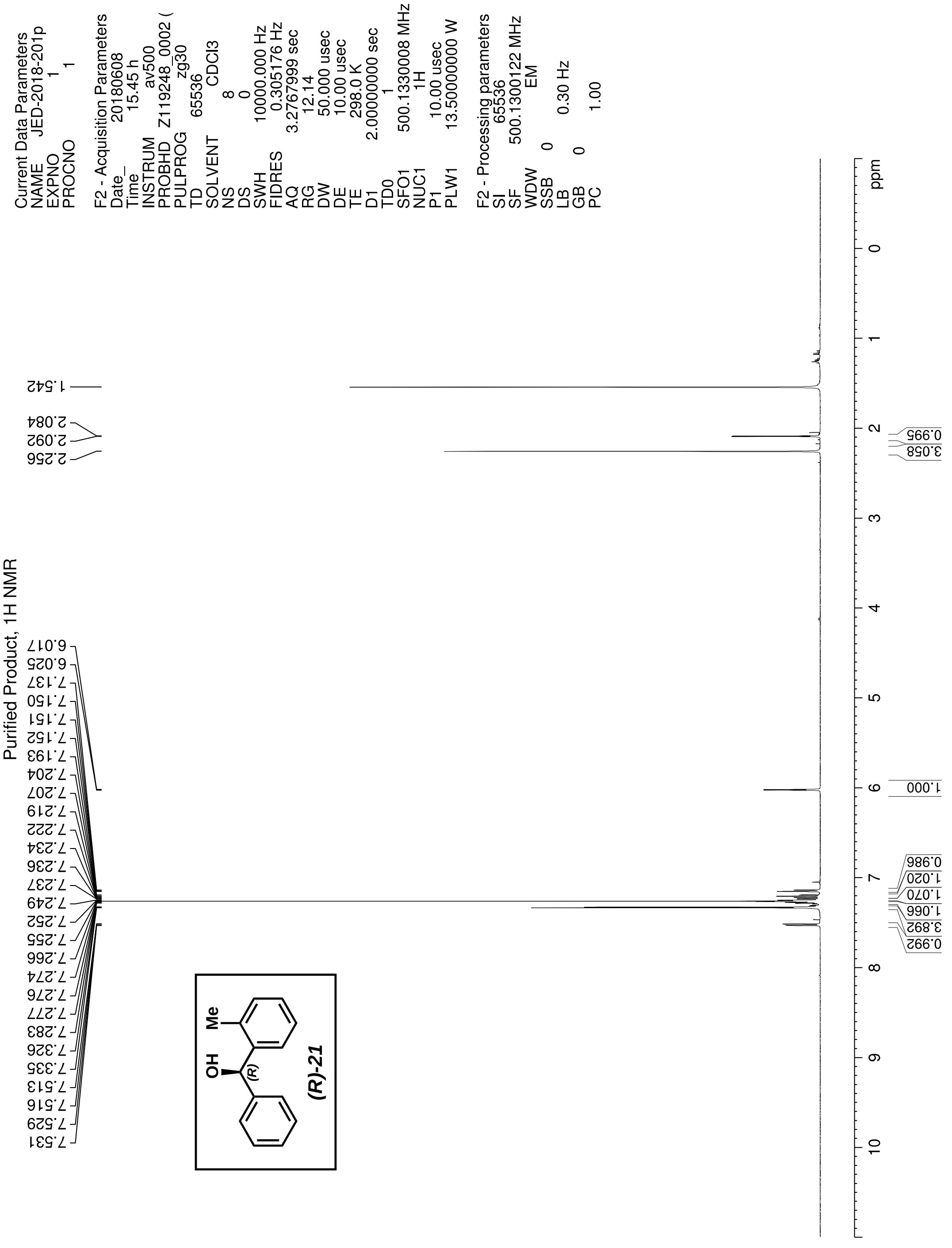

Supplementary Figure 24. ^{1}H NMR spectrum for compound ***(R)-21***.

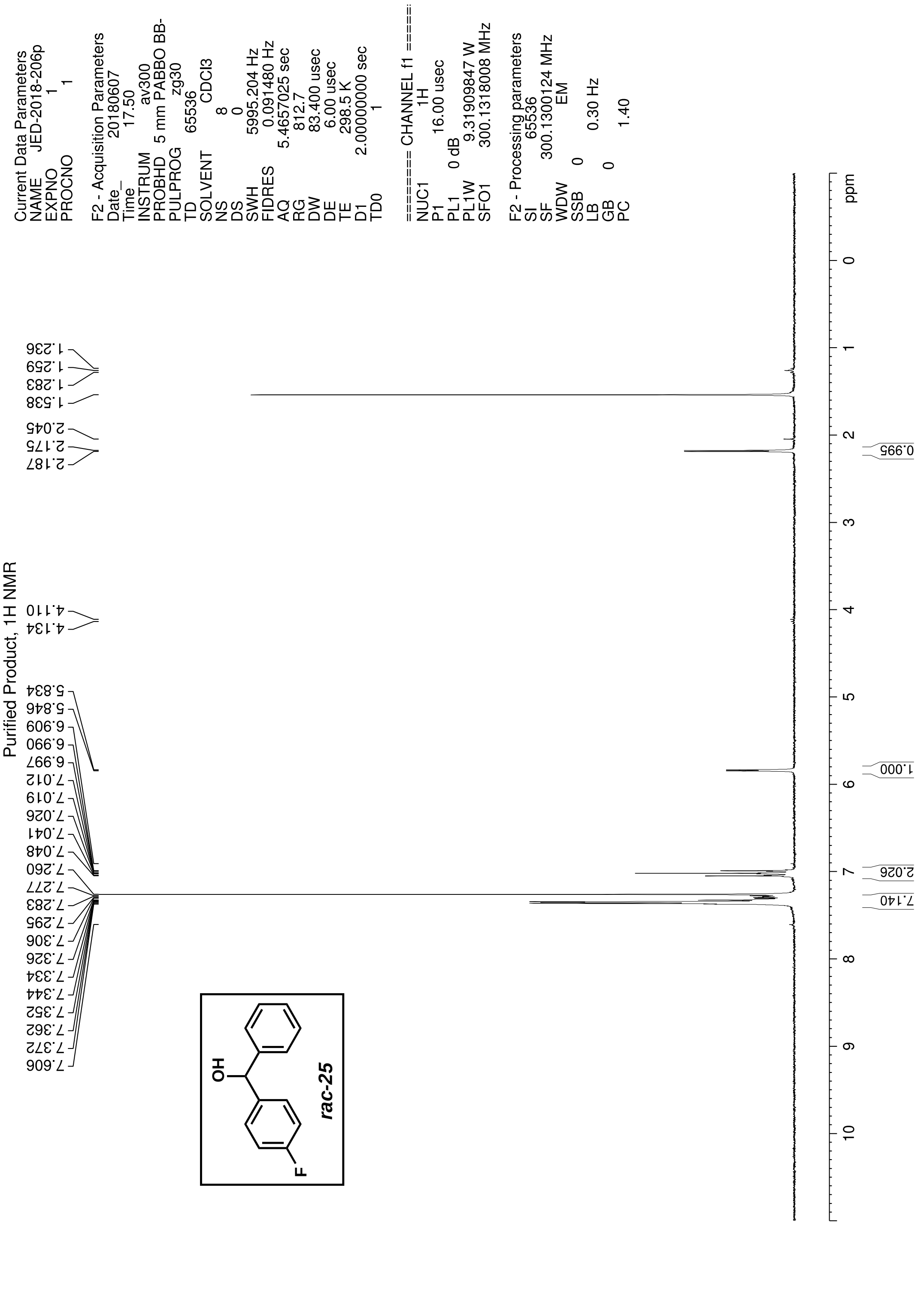

Supplementary Figure 25. ^{1}H NMR spectrum for compound ***rac-25***.

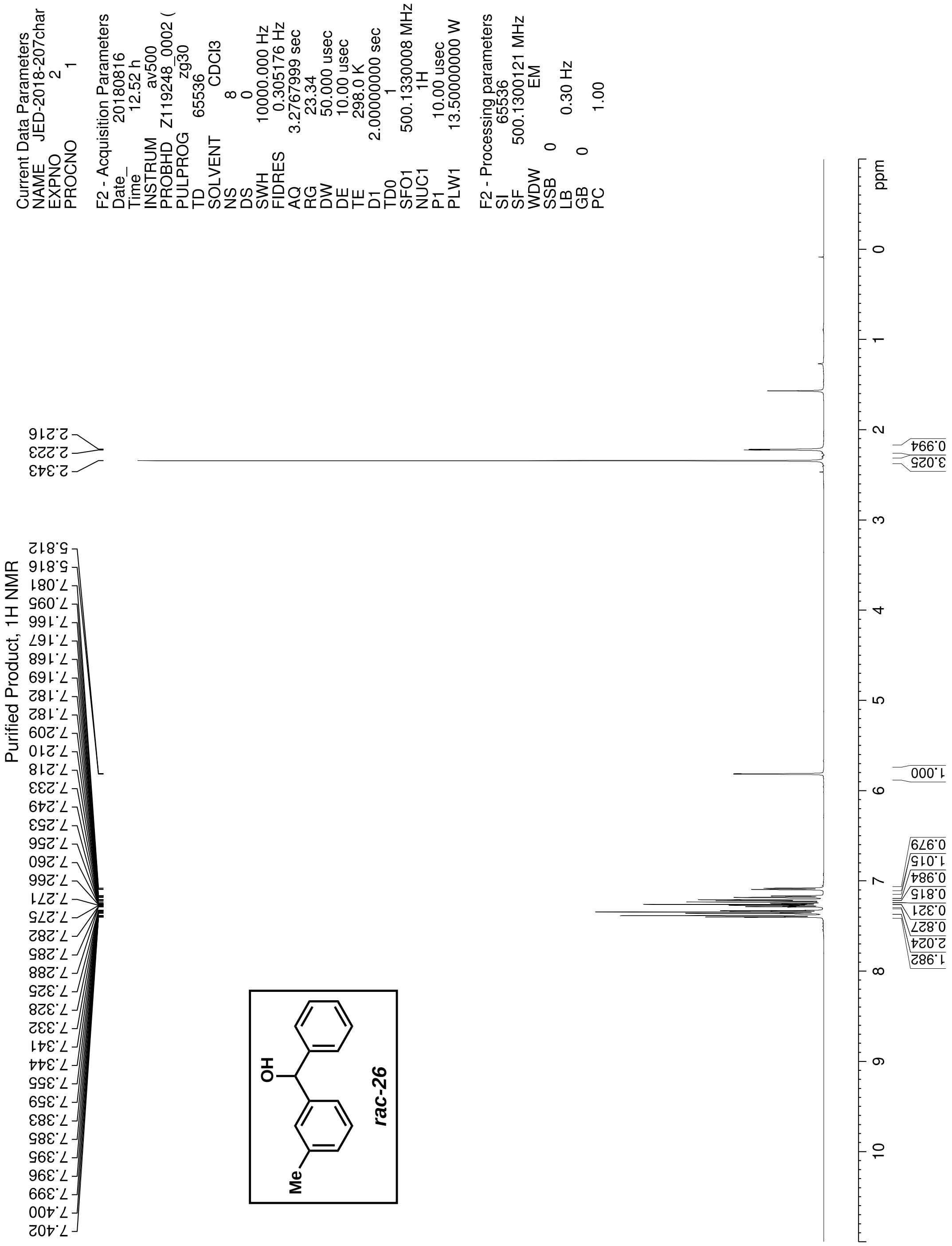

Supplementary Figure 26. ^{1}H NMR spectrum for compound ***rac-26***.

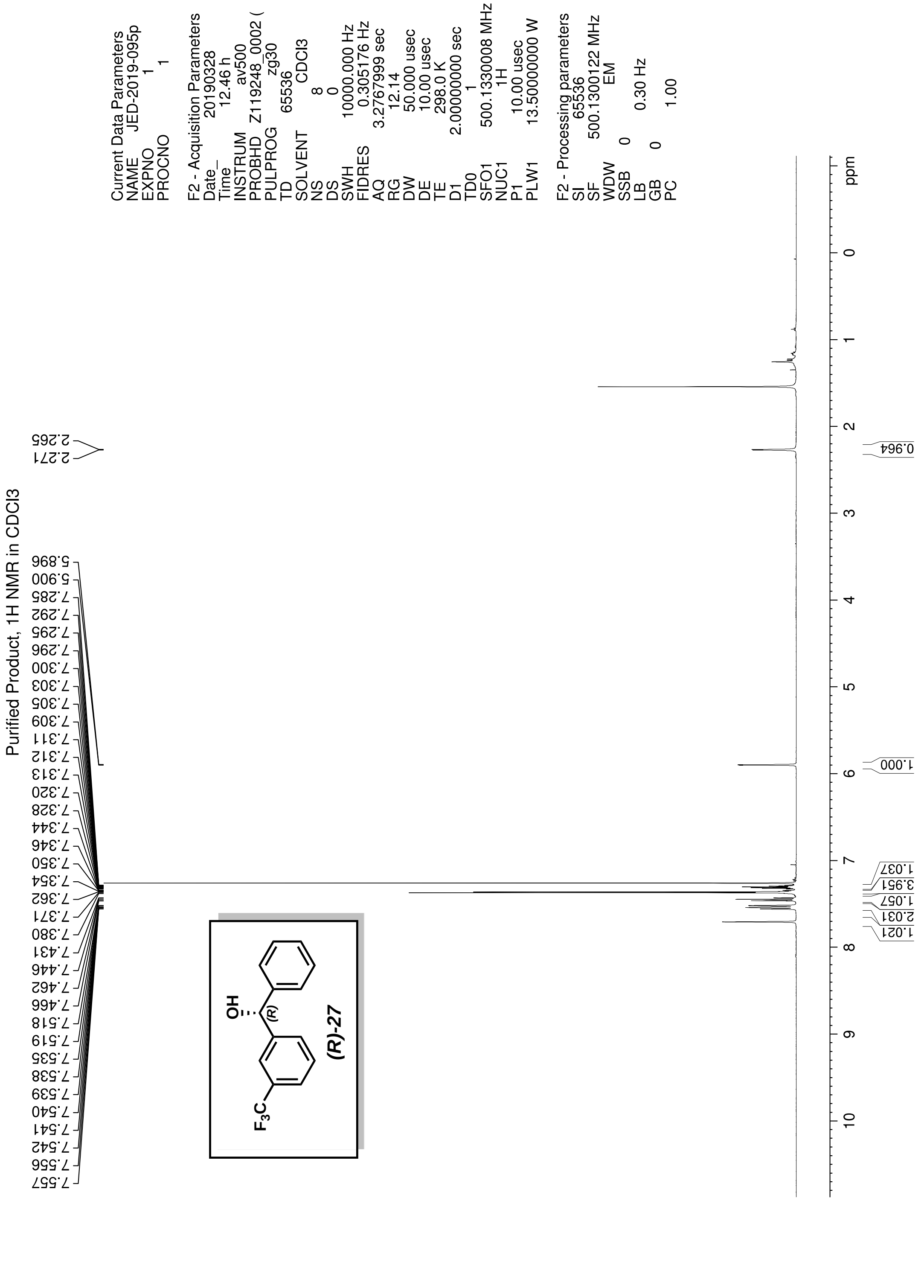

Supplementary Figure 27. ^{1}H NMR spectrum for compound ***(R)-27***.

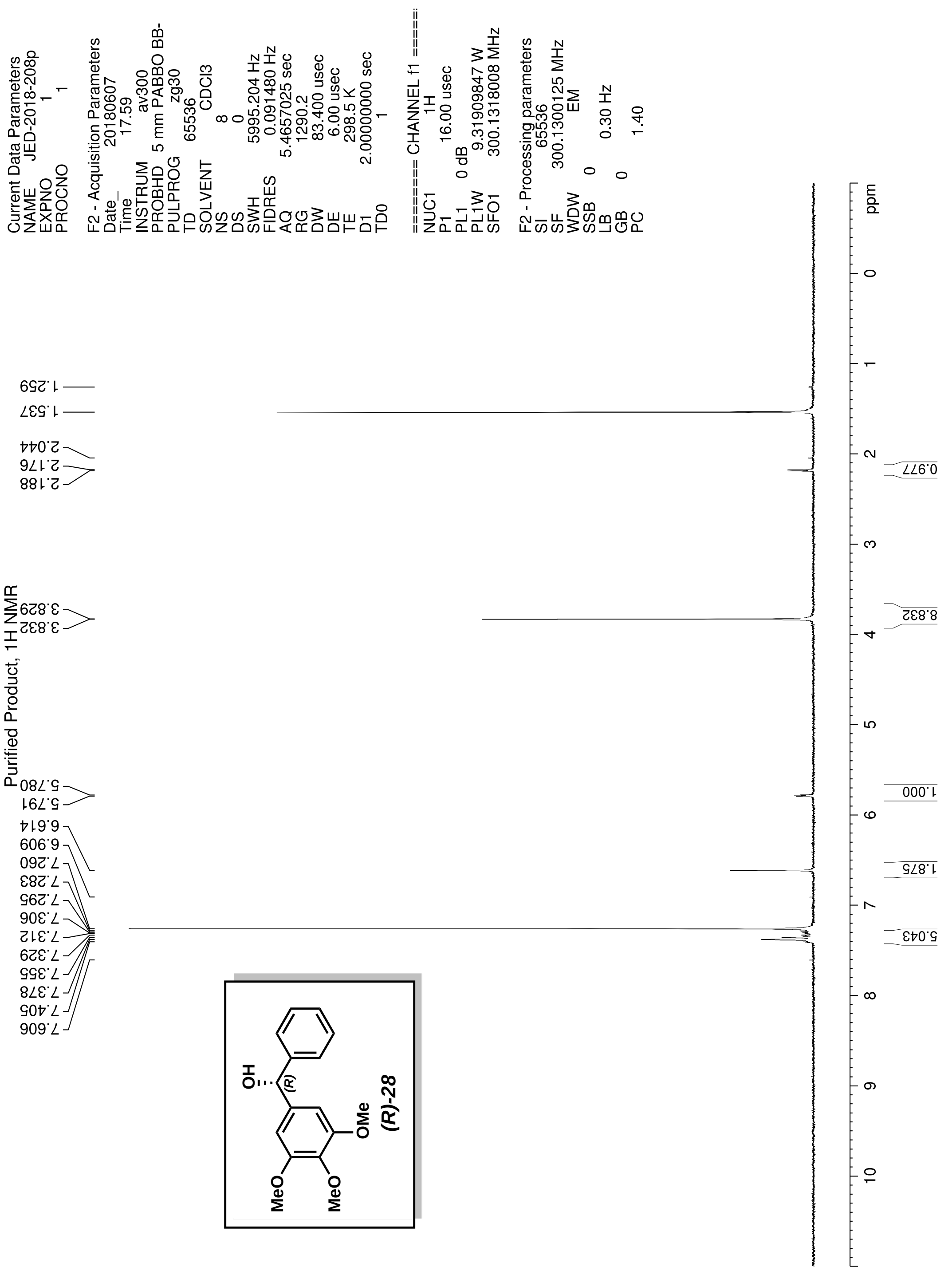

Supplementary Figure 28. ^{1}H NMR spectrum for compound ***(R)-28***.

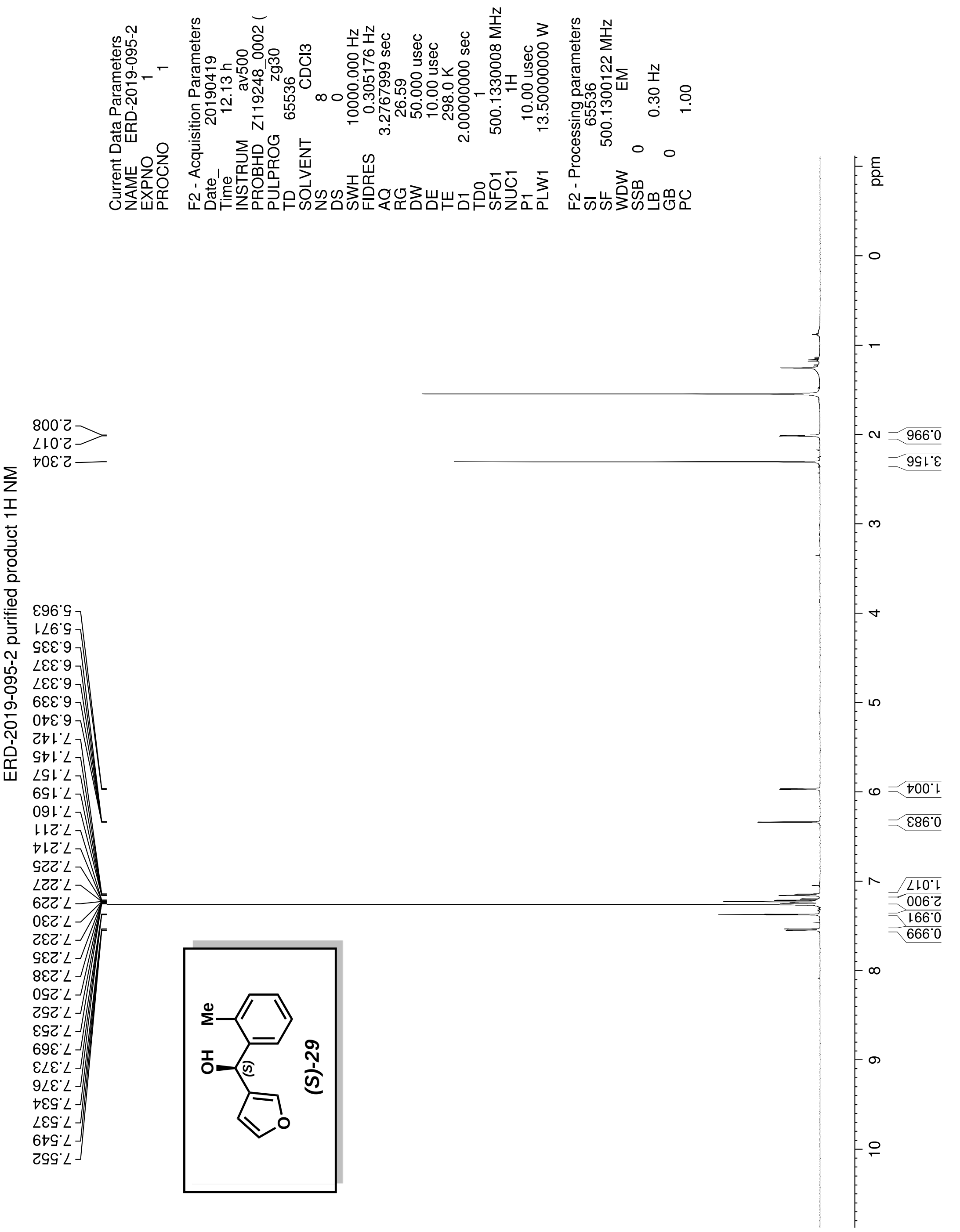

Supplementary Figure 29. ^{1}H NMR spectrum for compound ***(S)-29***.

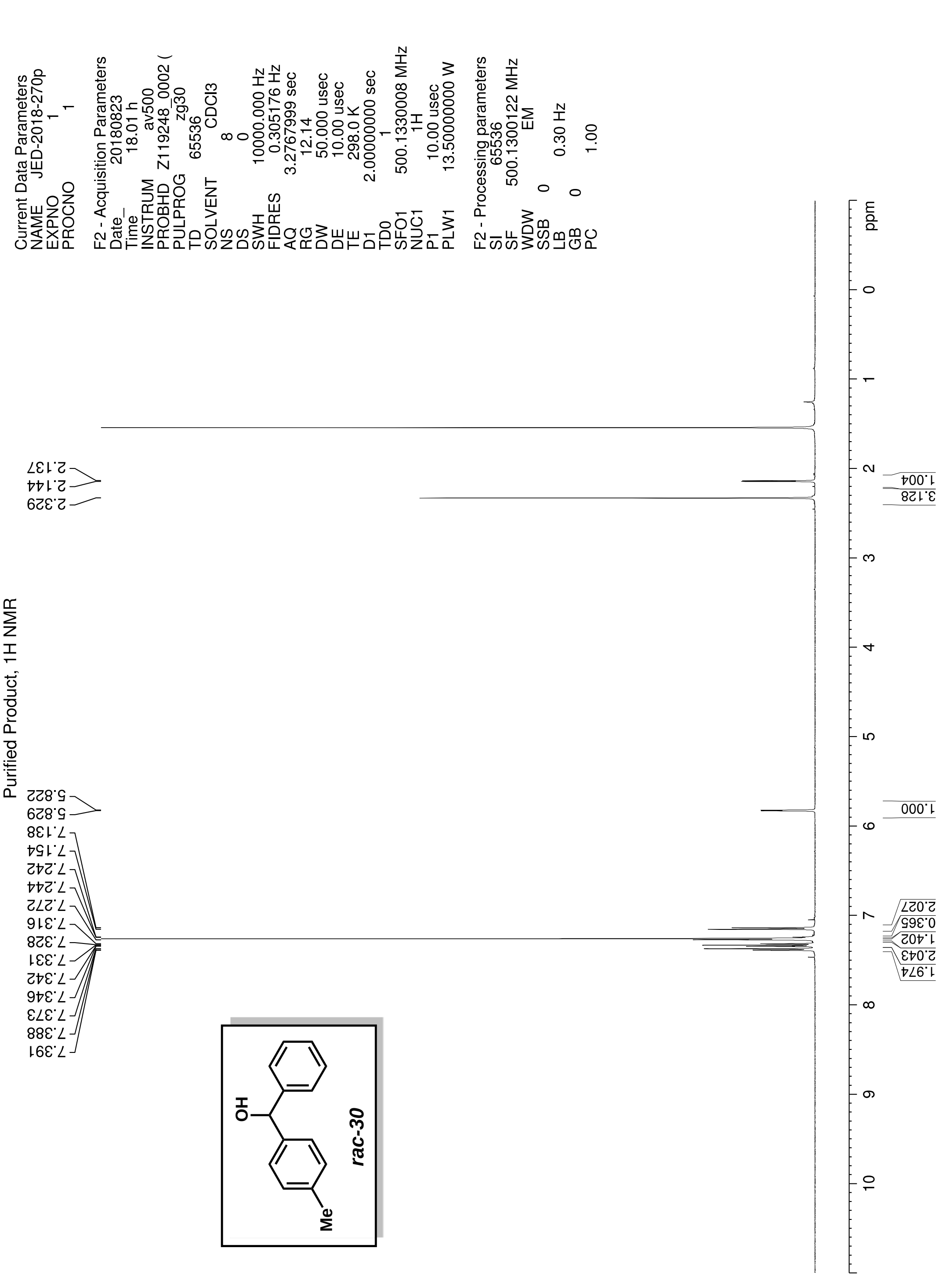

Supplementary Figure 30. ^{1}H NMR spectrum for compound ***rac-30***.

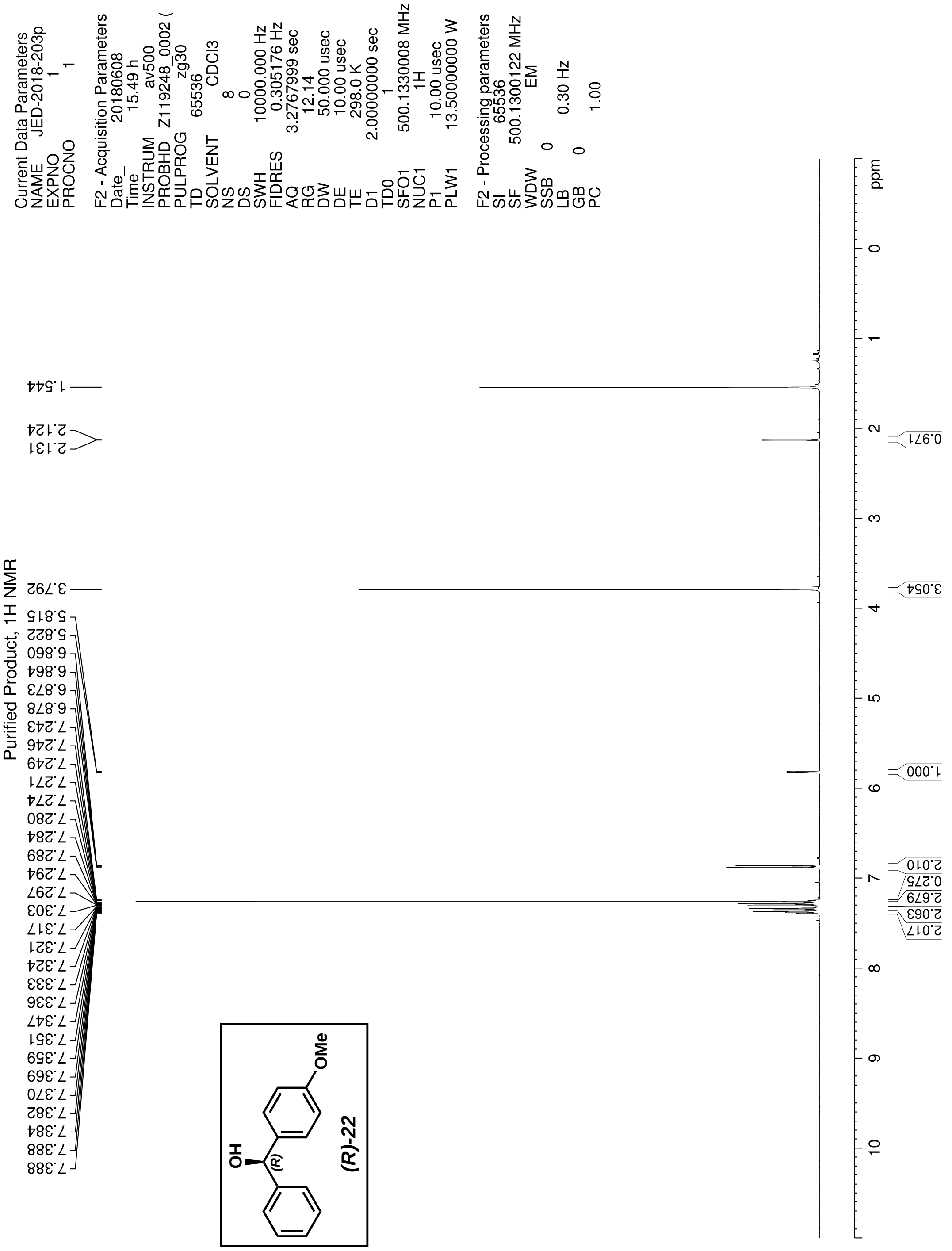

Supplementary Figure 31. ^{1}H NMR spectrum for compound ***(R)-22***.

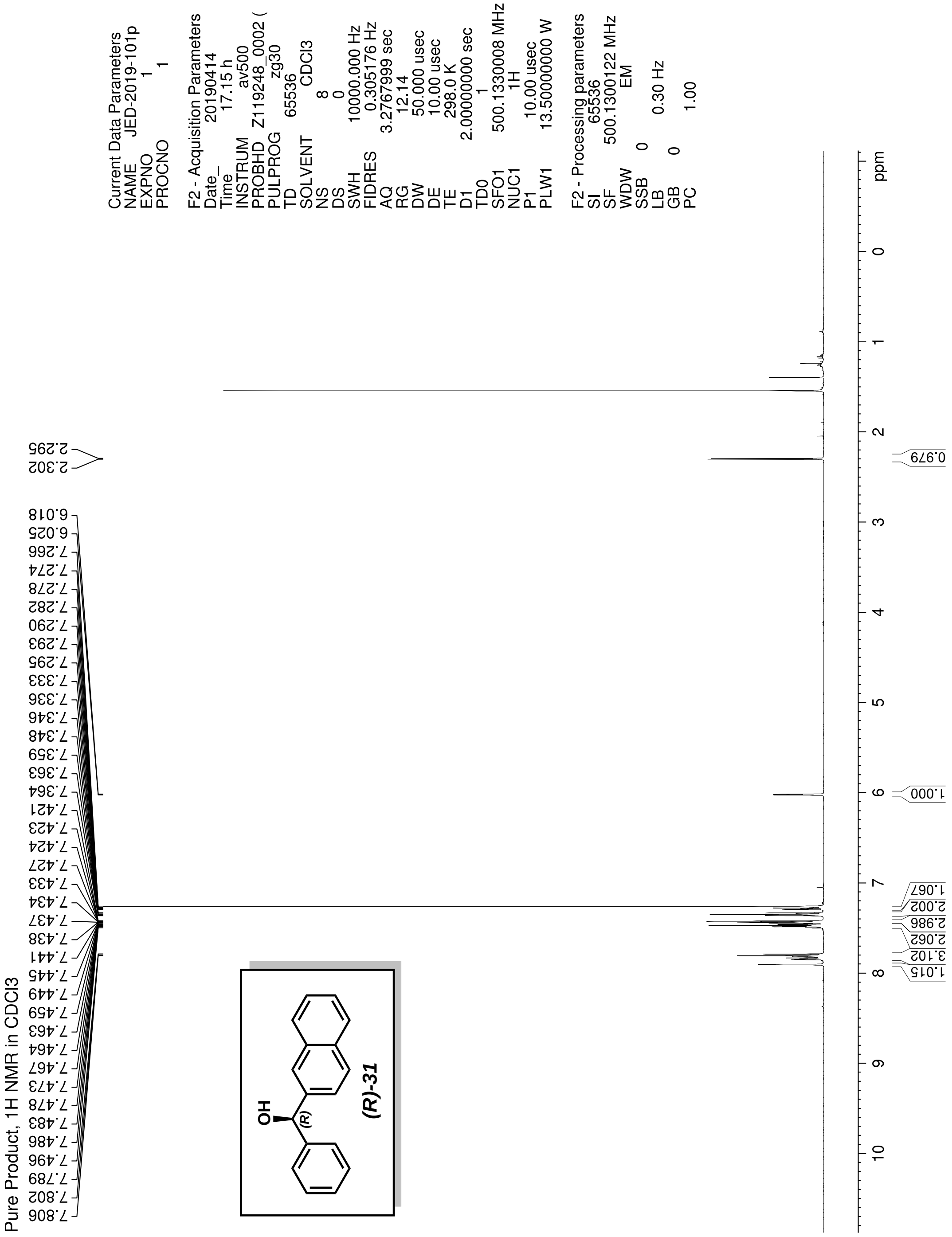

Supplementary Figure 32. ¹H NMR spectrum for compound ***(R)-31***.

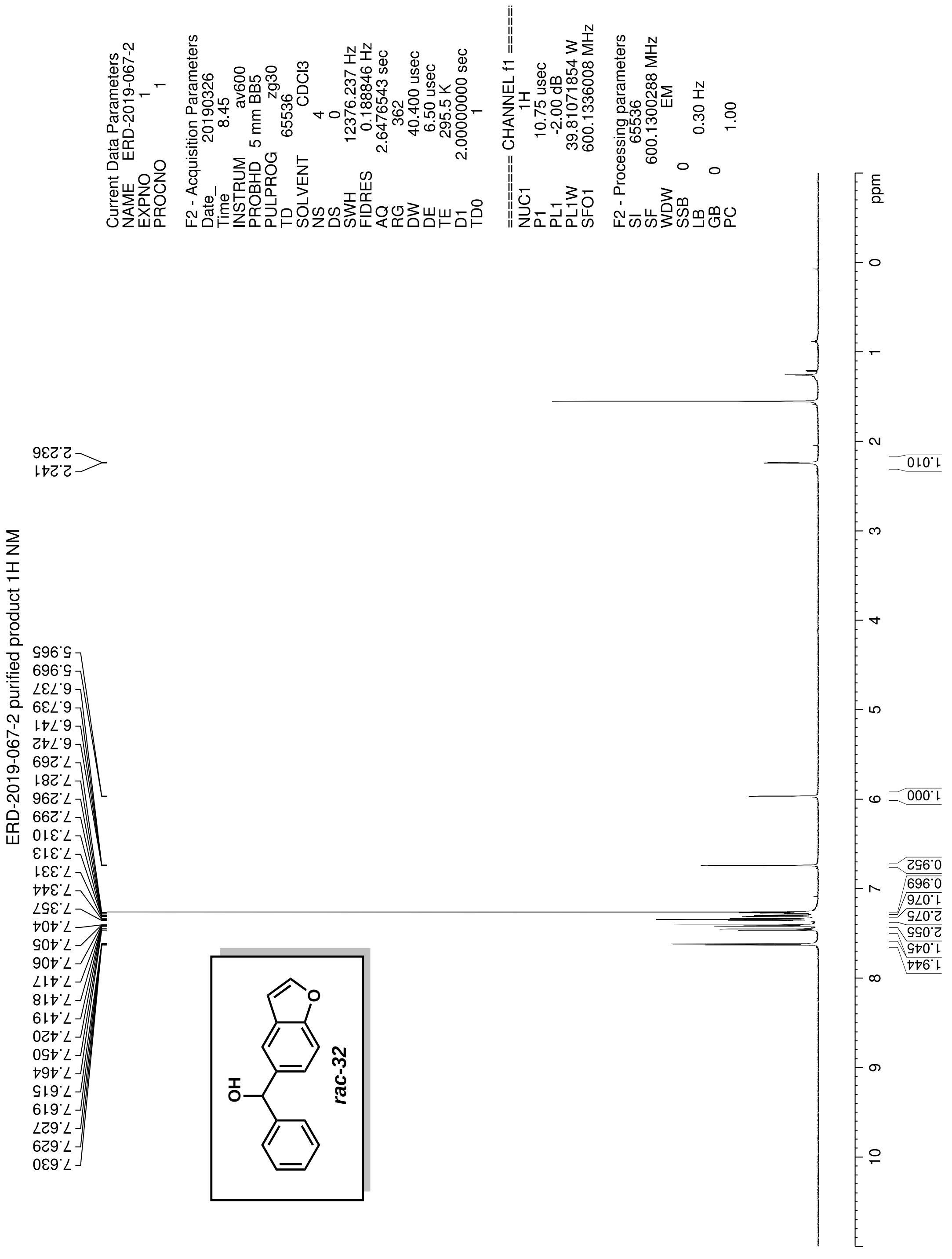

Supplementary Figure 33. ^{1}H NMR spectrum for compound ***rac-32***.

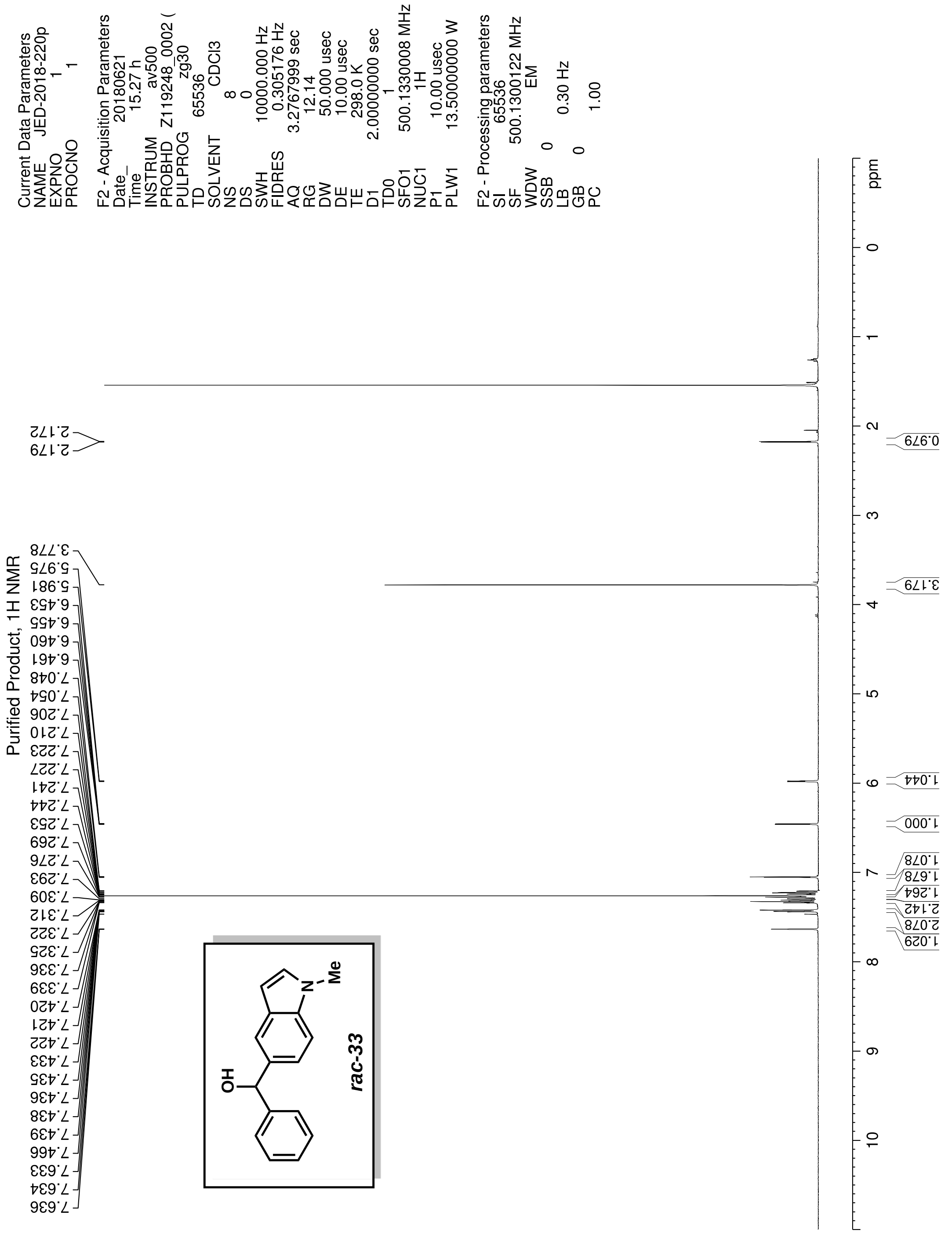

Supplementary Figure 34. ^{1}H NMR spectrum for compound ***rac-33***.

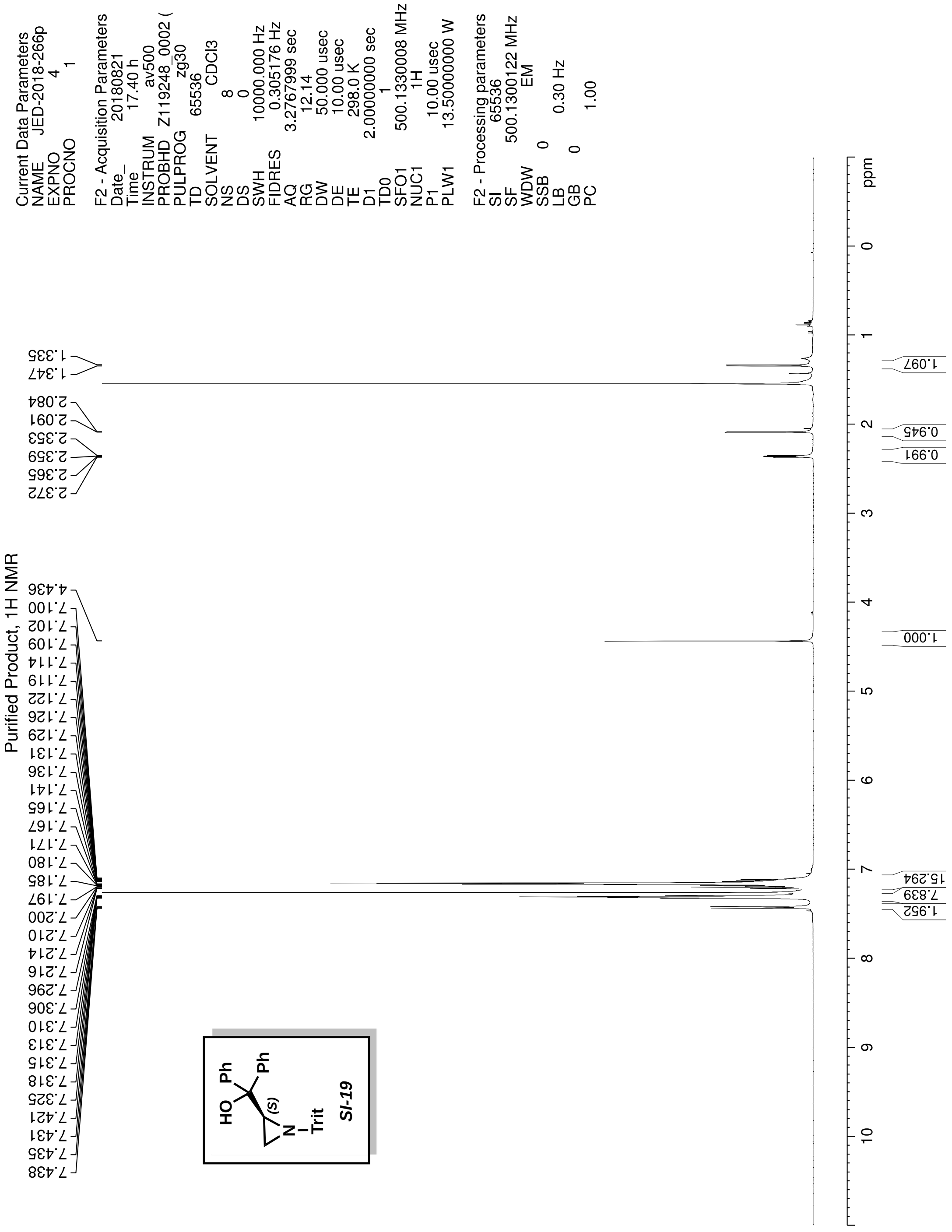

Supplementary Figure 35. ^{1}H NMR spectrum for compound ***SI-19***.

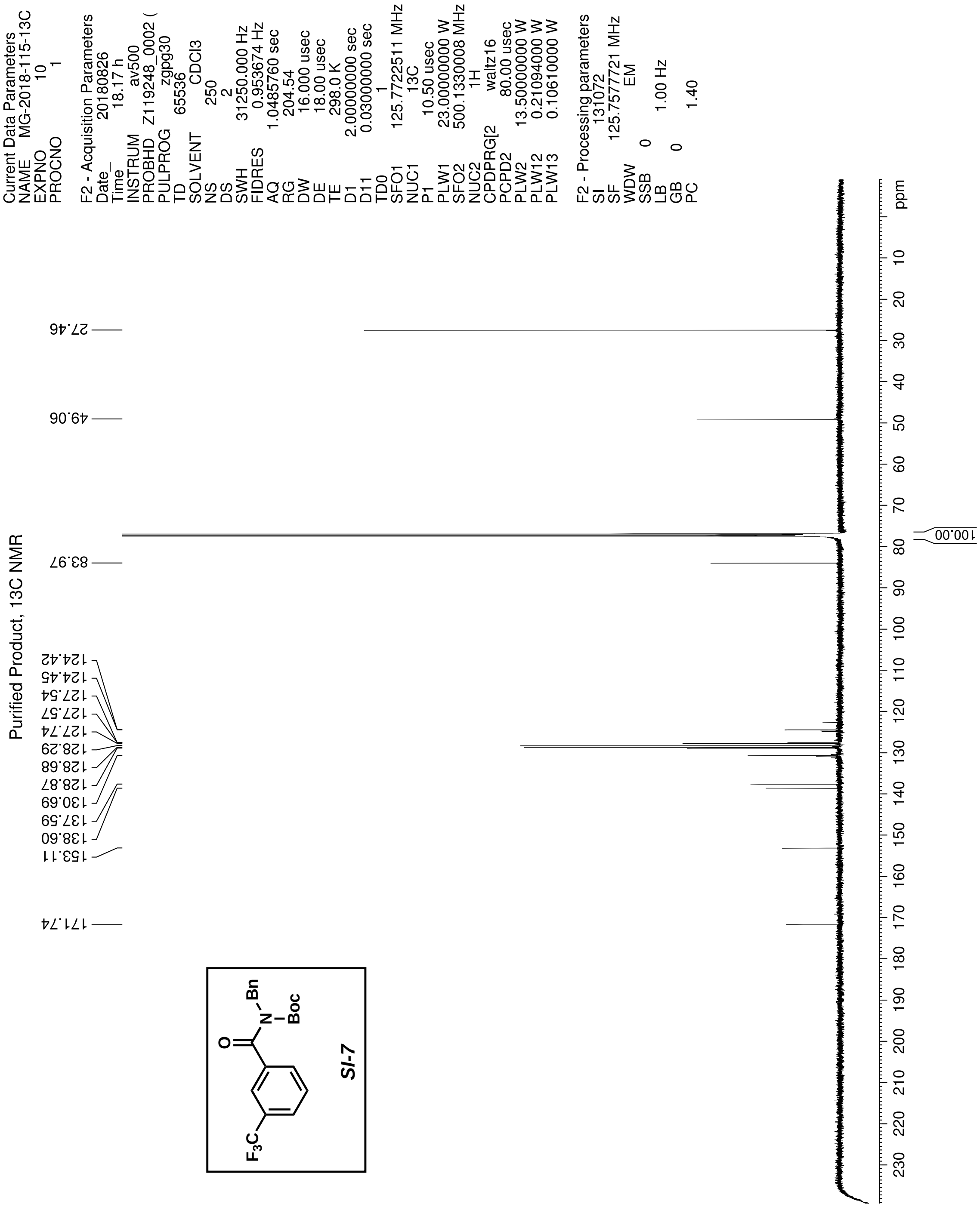

Supplementary Figure 36. ^{13}C NMR spectrum for compound ***SI-7***.

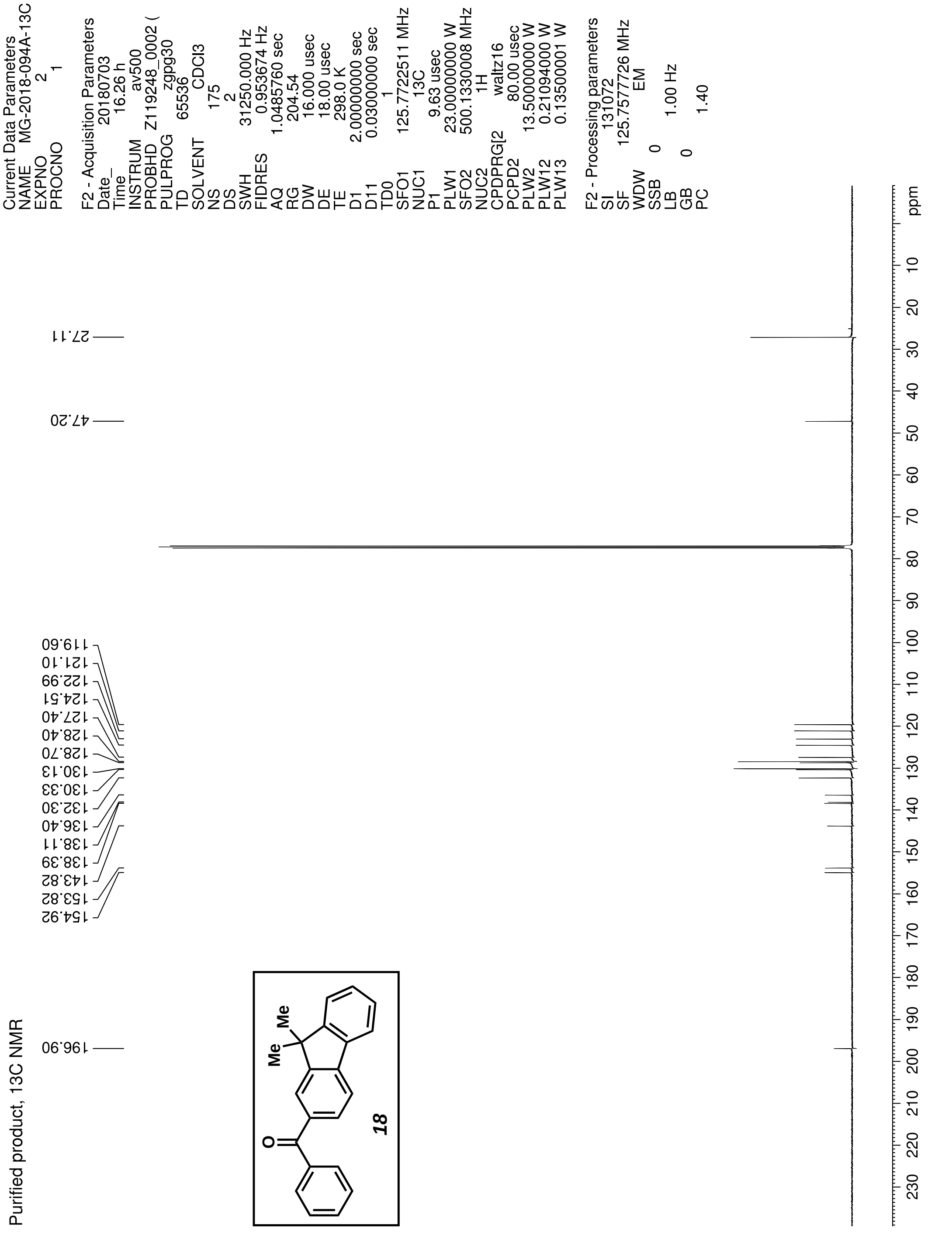

Supplementary Figure 37. ^{13}C NMR spectrum for compound ***18***.

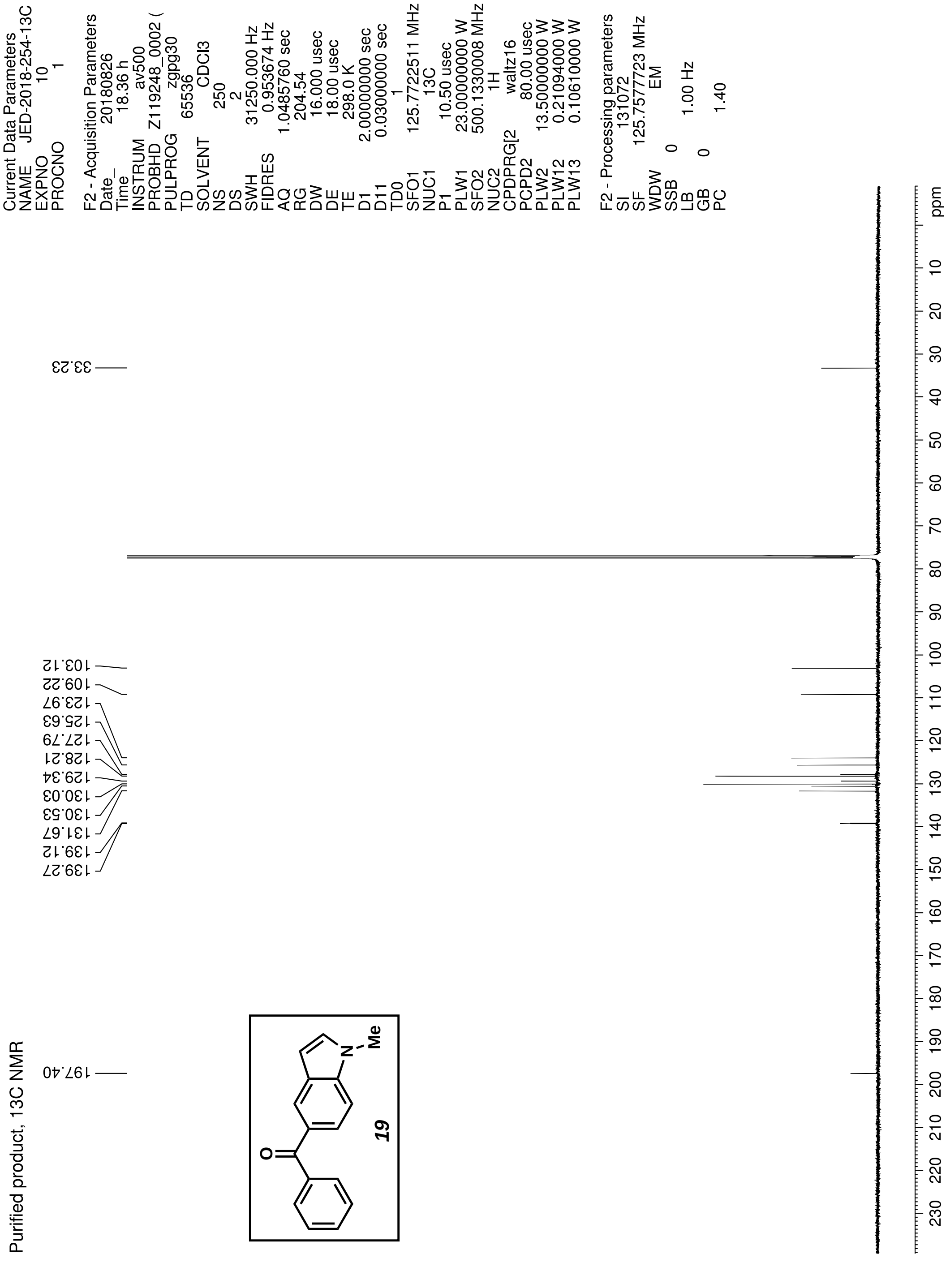

Supplementary Figure 38. ^{13}C NMR spectrum for compound ***19***.

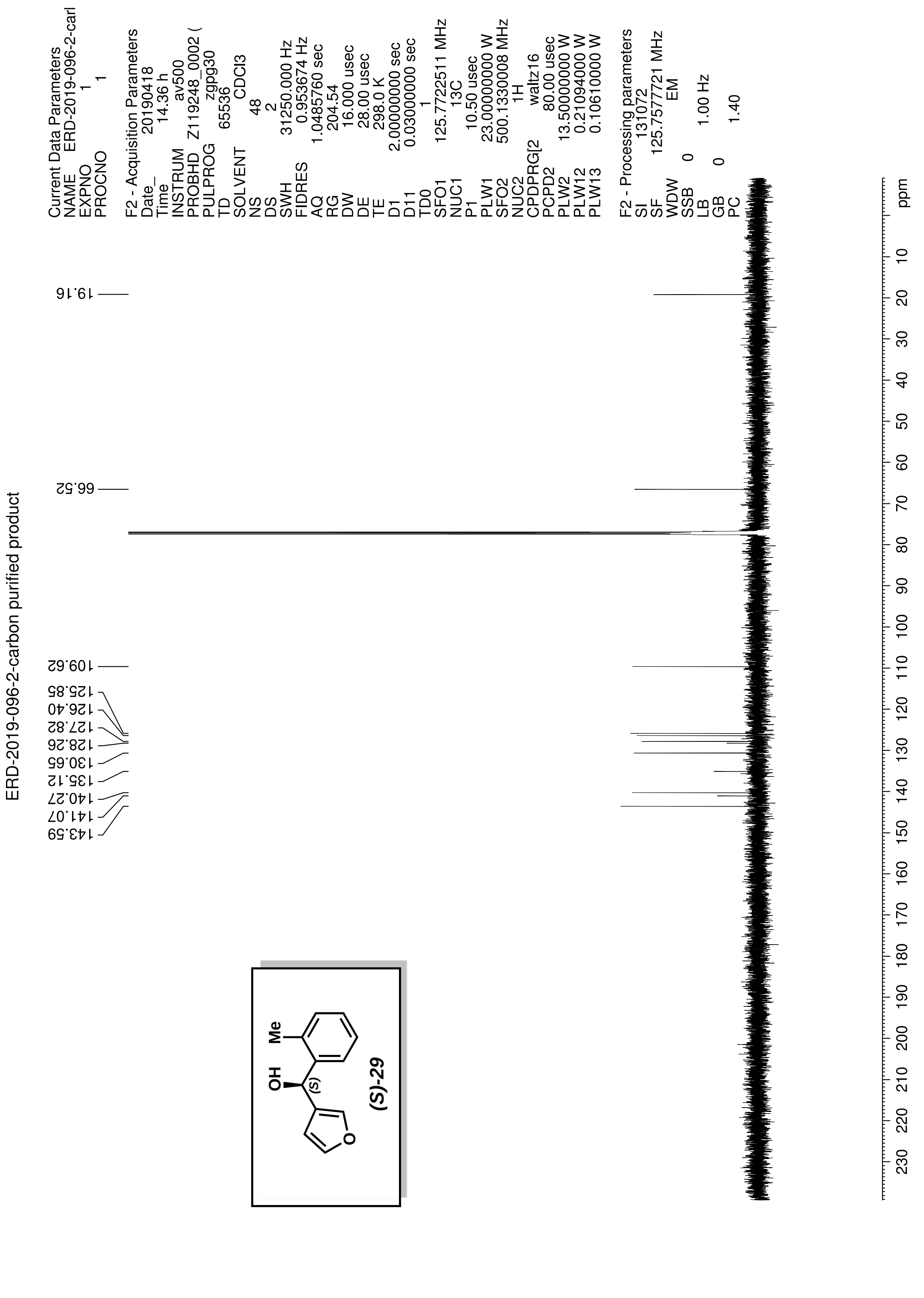

Supplementary Figure 39. ^{13}C NMR spectrum for compound ***(S)-29***.

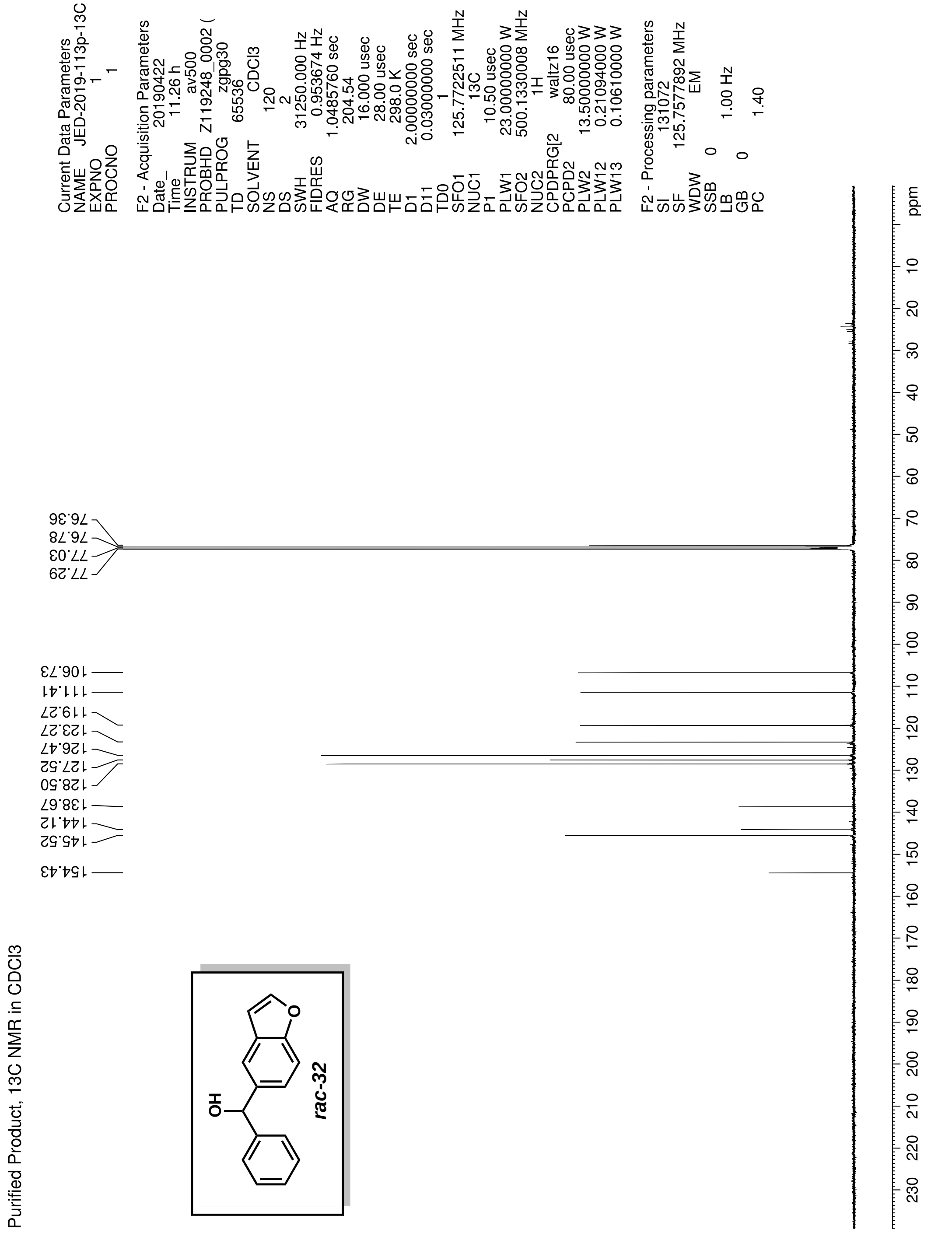

Supplementary Figure 40. ^{13}C NMR spectrum for compound ***rac-32***.

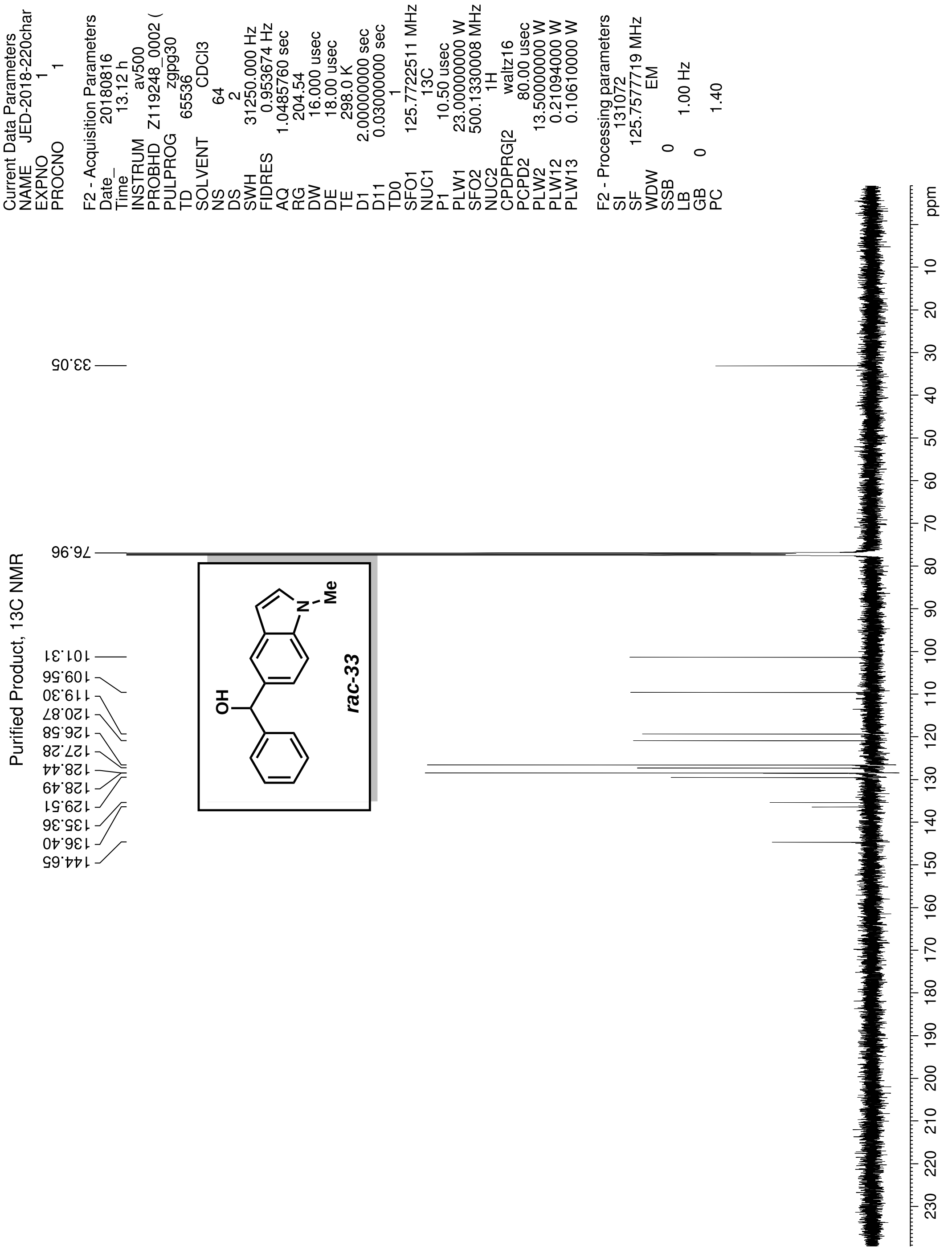

Supplementary Figure 41. ^{13}C NMR spectrum for compound ***rac-33***.

SFC Traces from Figure 2 and Supplementary Table 2: Evaluation of KREDs in the reduction of ketones **14** and **16**.

Supplementary Figure 42. SFC Trace of ***rac*-21**

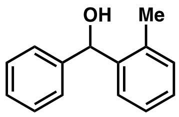

rac-21

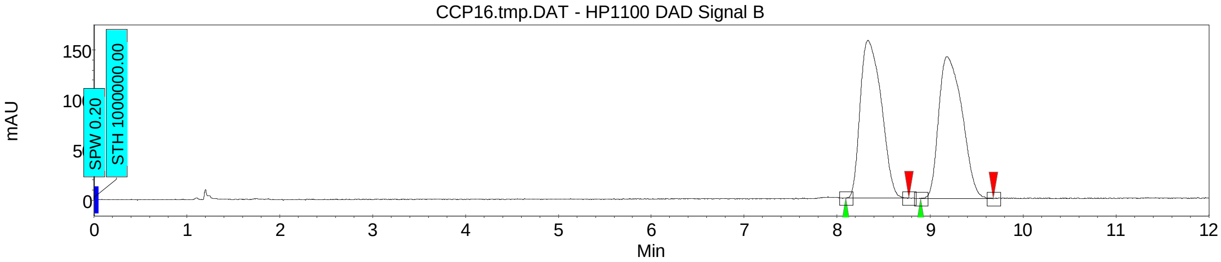

Index	Name	Start	Time	End	RT Offset	Quantity	Height	Area	Area
		[Min]	[Min]	[Min]	[Min]	[% Area]	[µV]	[µV.Min]	[%]
1	UNKNOWN	8.09	8.33	8.77	0.00	50.05	157.1	41.3	50.050
2	UNKNOWN	8.90	9.18	9.68	0.00	49.95	141.4	41.2	49.950
Total						100.00	298.5	82.5	100.000

Supplementary Figure 43. SFC trace of ***(S)*-21** (Supplementary Table 2, Entry 1)

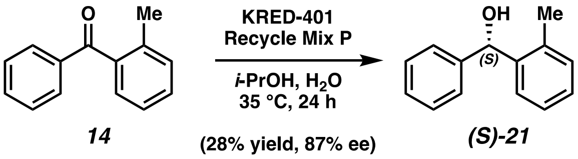

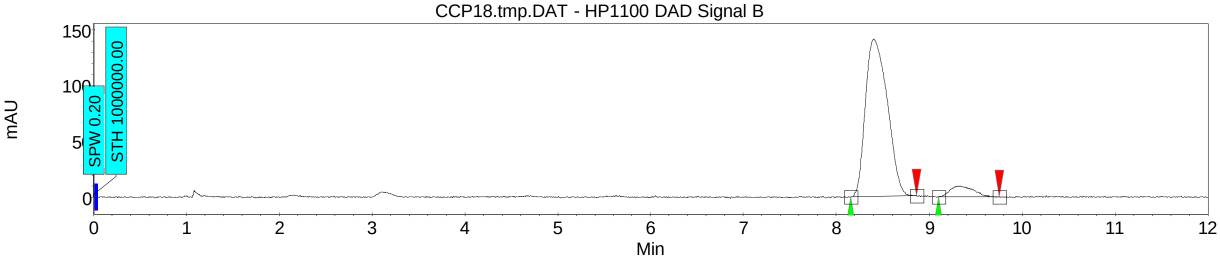

Index	Name	Start	Time	End	RT Offset	Quantity	Height	Area	Area
		[Min]	[Min]	[Min]	[Min]	[% Area]	[µV]	[µV.Min]	[%]
1	UNKNOWN	8.15	8.40	8.86	0.00	93.30	140.8	38.5	93.302
2	UNKNOWN	9.10	9.33	9.76	0.00	6.70	9.7	2.8	6.698
Total						100.00	150.5	41.2	100.000

Supplementary Figure 44. SFC trace of ***(S)*-21** (Supplementary Table 2, Entry 2)

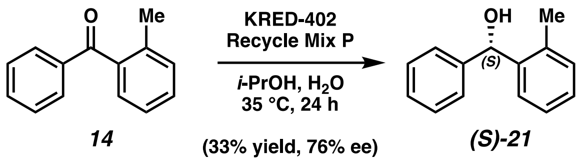

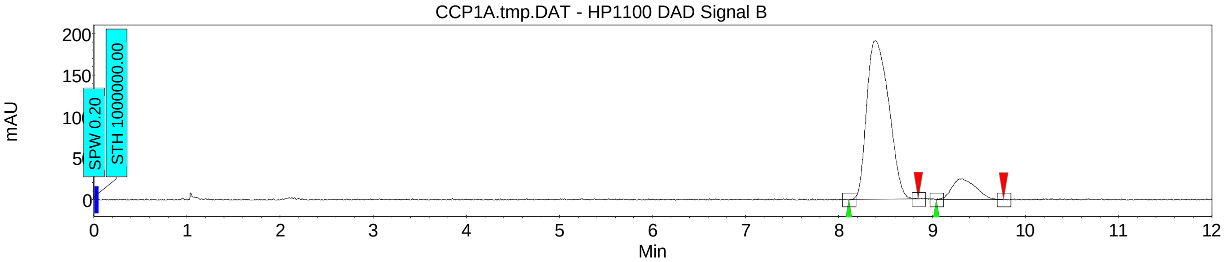

Index	Name	Start	Time	End	RT Offset	Quantity	Height	Area	Area
		[Min]	[Min]	[Min]	[Min]	[% Area]	[µV]	[µV.Min]	[%]
1	UNKNOWN	8.10	8.39	8.85	0.00	87.88	190.9	52.4	87.883
2	UNKNOWN	9.05	9.29	9.77	0.00	12.12	24.5	7.2	12.117
Total						100.00	215.4	59.6	100.000

Supplementary Figure 45. SFC trace of ***(S)*-21** (Supplementary Table 2, Entry 3)

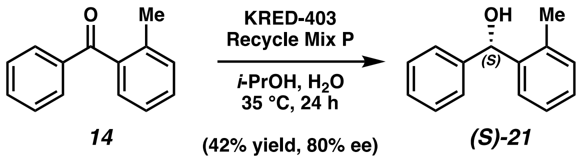

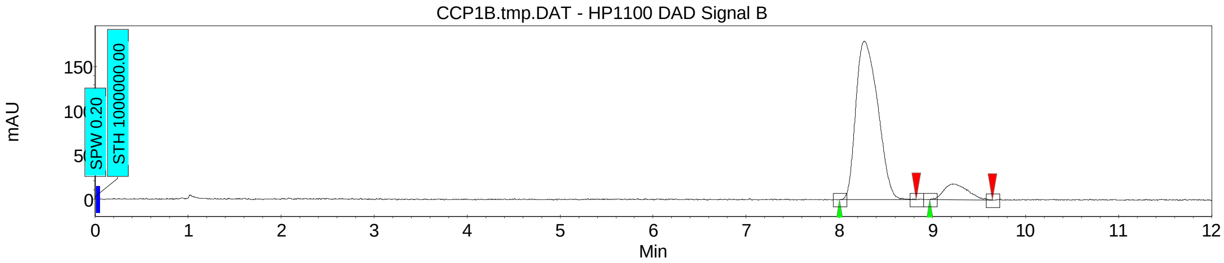

Index	Name	Start	Time	End	RT Offset	Quantity	Height	Area	Area
		[Min]	[Min]	[Min]	[Min]	[% Area]	[µV]	[µV.Min]	[%]
1	UNKNOWN	8.01	8.27	8.82	0.00	90.07	179.6	49.2	90.075
2	UNKNOWN	8.97	9.22	9.64	0.00	9.93	17.9	5.4	9.925
Total						100.00	197.5	54.6	100.000

Supplementary Figure 46. SFC trace of ***(S)*-21** (Supplementary Table 2, Entry 4)

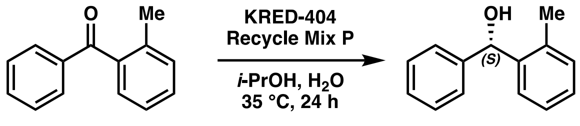

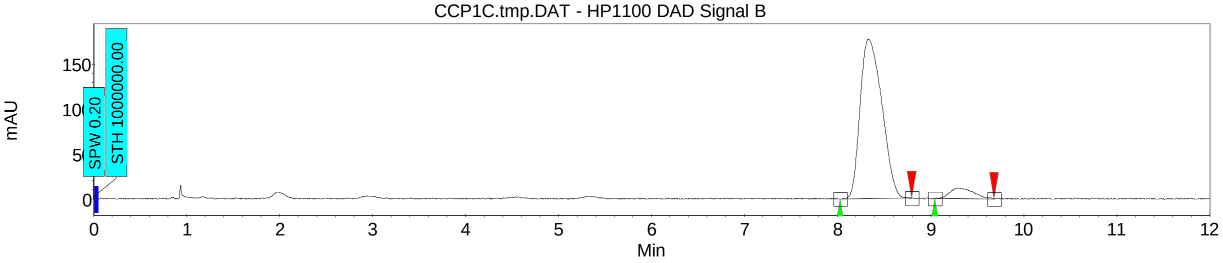

Index	Name	Start	Time	End	RT Offset	Quantity	Height	Area	Area
		[Min]	[Min]	[Min]	[Min]	[% Area]	[µV]	[µV.Min]	[%]
1	UNKNOWN	8.03	8.33	8.80	0.00	93.33	176.7	47.9	93.331
2	UNKNOWN	9.05	9.31	9.68	0.00	6.67	11.6	3.4	6.669
Total						100.00	188.4	51.4	100.000

Supplementary Figure 47. SFC trace of ***(R)*-21** (Supplementary Table 2, Entry 5)

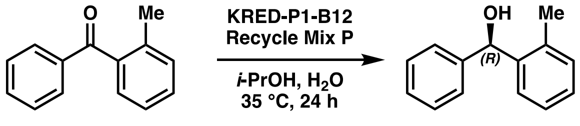

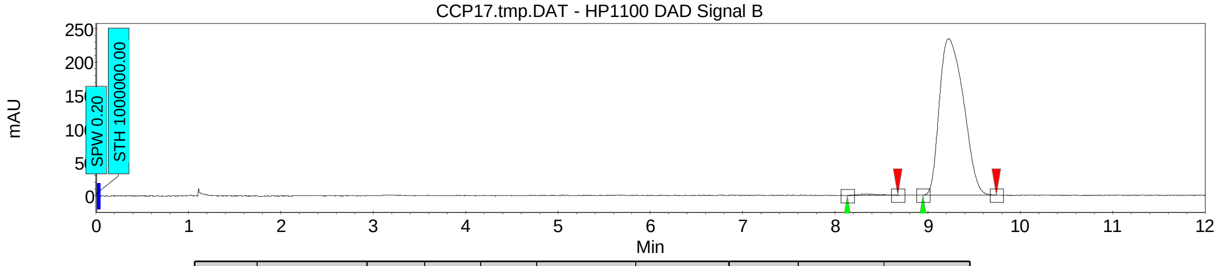

Index	Name	Start	Time	End	RT Offset	Quantity	Height	Area	Area
		[Min]	[Min]	[Min]	[Min]	[% Area]	[µV]	[µV.Min]	[%]
1	UNKNOWN	8.13	8.35	8.68	0.00	0.72	2.2	0.5	0.720
2	UNKNOWN	8.95	9.23	9.74	0.00	99.28	233.7	70.6	99.280
Total						100.00	235.9	71.1	100.000

Supplementary Figure 48. SFC Trace of ***rac*-22**

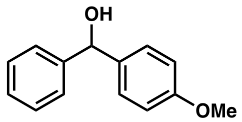

rac-22

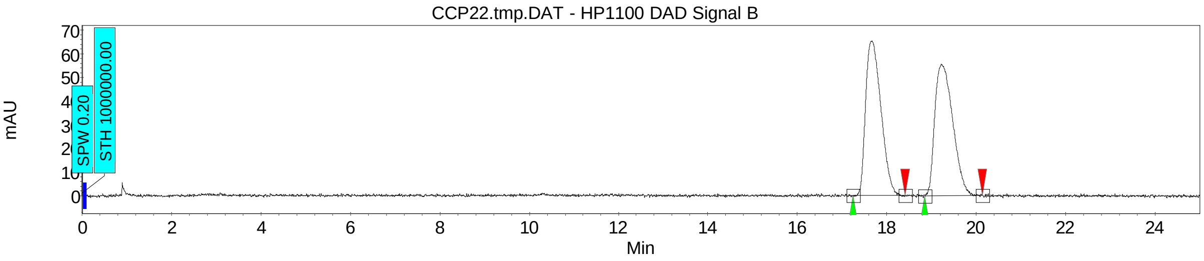

Index	Name	Start	Time	End	RT Offset	Quantity	Height	Area	Area
		[Min]	[Min]	[Min]	[Min]	[% Area]	[µV]	[µV.Min]	[%]
1	UNKNOWN	17.25	17.67	18.41	0.00	50.12	65.4	26.0	50.119
2	UNKNOWN	18.85	19.22	20.14	0.00	49.88	55.7	25.9	49.881
Total						100.00	121.1	51.8	100.000

Supplementary Figure 49. SFC trace of ***(R)*-22** (Supplementary Table 2, Entry 6)

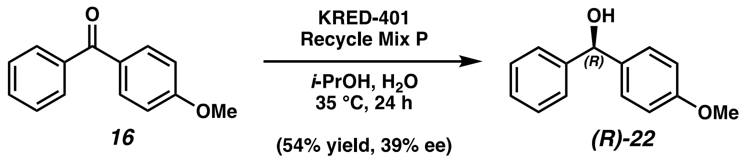

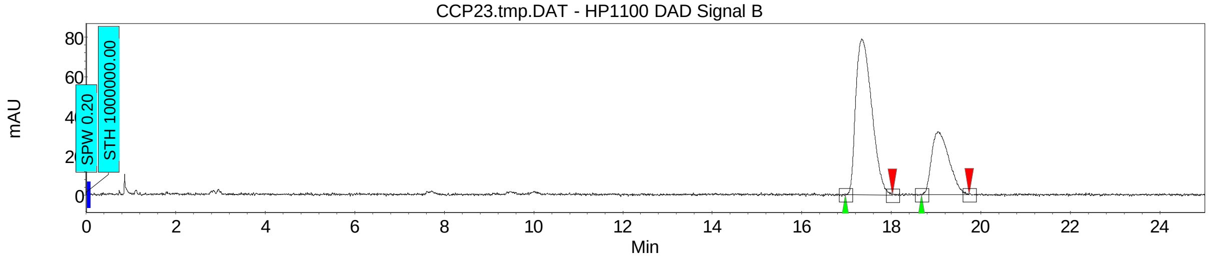

Index	Name	Start	Time	End	RT Offset	Quantity	Height	Area	Area
		[Min]	[Min]	[Min]	[Min]	[% Area]	[µV]	[µV.Min]	[%]
1	UNKNOWN	16.98	17.34	18.02	0.00	69.42	79.1	32.8	69.418
2	UNKNOWN	18.68	19.05	19.73	0.00	30.58	32.1	14.4	30.582
Total						100.00	111.1	47.2	100.000

Supplementary Figure 50. SFC trace of ***(R)*-22** (Supplementary Table 2, Entry 7)

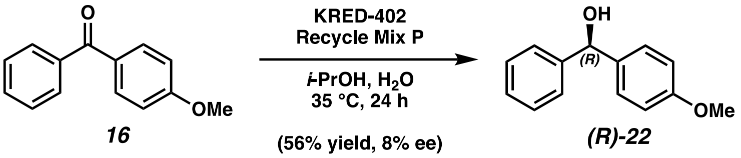

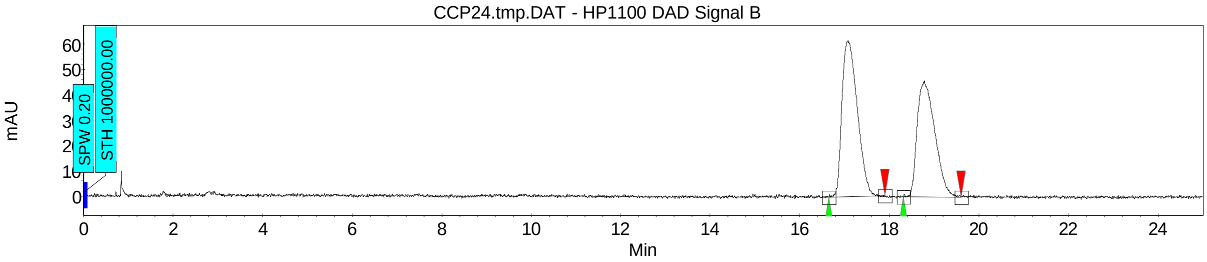

Index	Name	Start	Time	End	RT Offset	Quantity	Height	Area	Area
		[Min]	[Min]	[Min]	[Min]	[% Area]	[µV]	[µV.Min]	[%]
1	UNKNOWN	16.65	17.08	17.89	0.00	54.08	61.4	24.5	54.078
2	UNKNOWN	18.31	18.78	19.60	0.00	45.92	45.8	20.8	45.922
Total						100.00	107.3	45.4	100.000

Supplementary Figure 51. SFC trace of ***(R)*-22** (Supplementary Table 2, Entry 8)

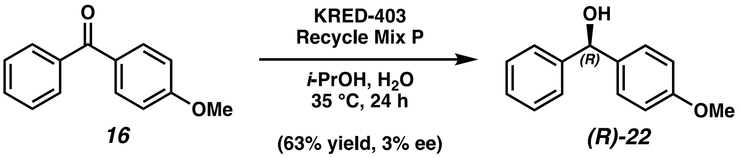

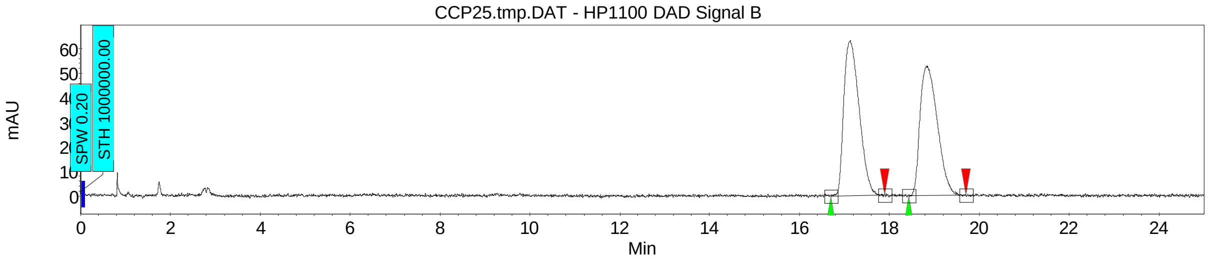

Index	Name	Start	Time	End	RT Offset	Quantity	Height	Area	Area
		[Min]	[Min]	[Min]	[Min]	[% Area]	[µV]	[µV.Min]	[%]
1	UNKNOWN	16.70	17.13	17.89	0.00	51.46	63.2	25.0	51.462
2	UNKNOWN	18.43	18.85	19.70	0.00	48.54	52.9	23.6	48.538
Total						100.00	116.1	48.6	100.000

Supplementary Figure 52. SFC trace of ***(S)*-22** (Supplementary Table 2, Entry 9)

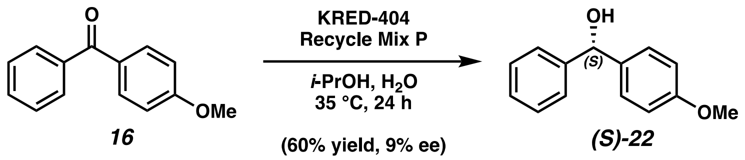

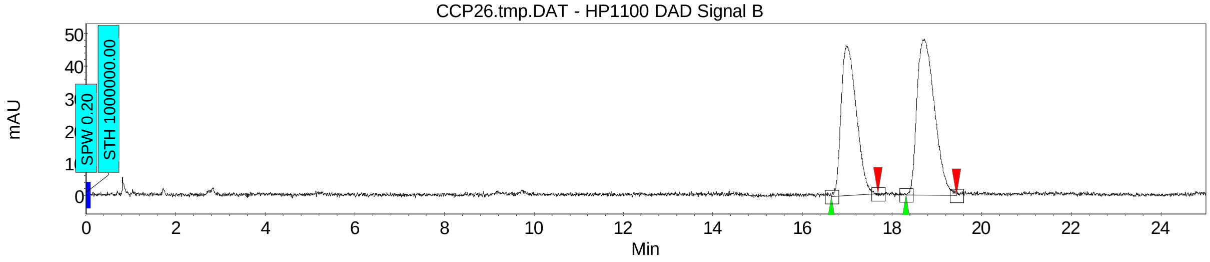

Index	Name	Start	Time	End	RT Offset	Quantity	Height	Area	Area
		[Min]	[Min]	[Min]	[Min]	[% Area]	[µV]	[µV.Min]	[%]
1	UNKNOWN	16.65	16.98	17.69	0.00	45.35	46.4	18.2	45.349
2	UNKNOWN	18.31	18.72	19.43	0.00	54.65	48.1	22.0	54.651
Total						100.00	94.5	40.2	100.000

Supplementary Figure 53. SFC trace of ***(S)*-22** (Supplementary Table 2, Entry 10)

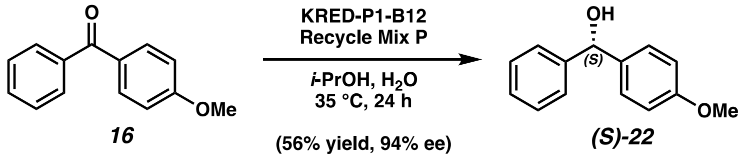

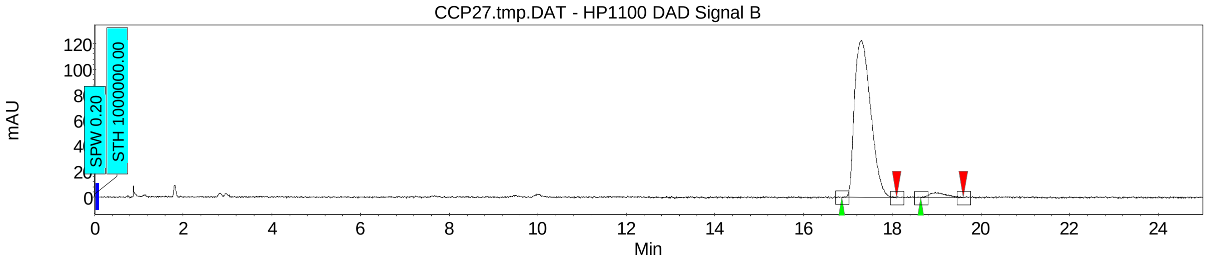

Index	Name	Start	Time	End	RT Offset	Quantity	Height	Area	Area
		[Min]	[Min]	[Min]	[Min]	[% Area]	[µV]	[µV.Min]	[%]
1	UNKNOWN	16.86	17.31	18.10	0.00	96.90	122.8	51.8	96.901
2	UNKNOWN	18.64	18.97	19.60	0.00	3.10	4.1	1.7	3.099
Total						100.00	126.8	53.5	100.000

SFC Traces from Figures 3 and 4a, and Section Gii of the Experimental Procedures: One-pot isolation and asymmetric arylation experiments.

Supplementary Figure 54. SFC Trace of ***rac*-25**

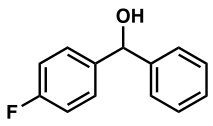

rac-25

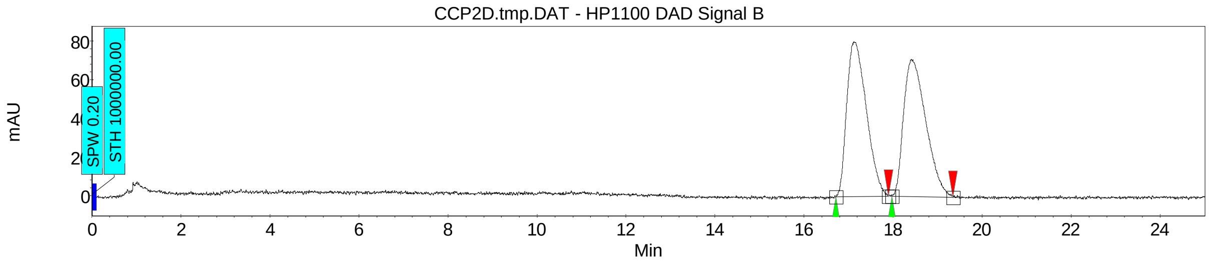

Index	Name	Start	Time	End	RT Offset	Quantity	Height	Area	Area
		[Min]	[Min]	[Min]	[Min]	[% Area]	[µV]	[µV.Min]	[%]
1	UNKNOWN	16.72	17.12	17.89	0.00	49.84	80.3	41.1	49.837
2	UNKNOWN	17.97	18.41	19.35	0.00	50.16	71.3	41.3	50.163
Total						100.00	151.6	82.4	100.000

Supplementary Figure 55. SFC Trace of ***(R)*-25**

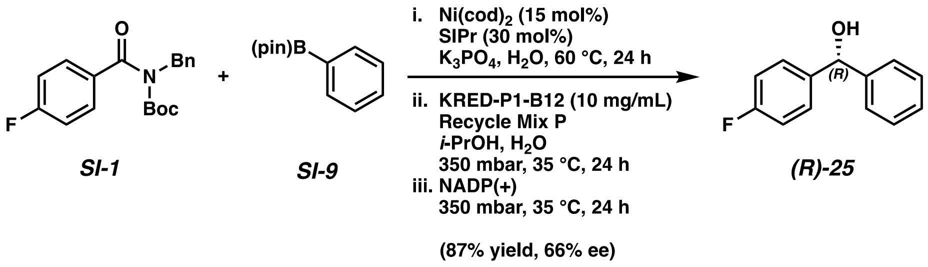

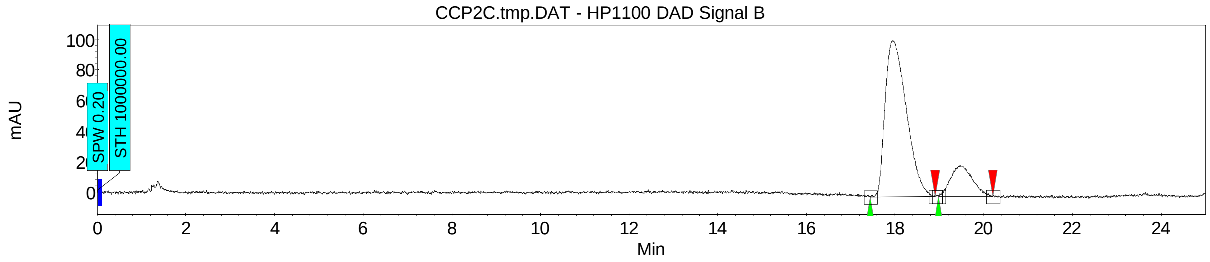

Index	Name	Start	Time	End	RT Offset	Quantity	Height	Area	Area
		[Min]	[Min]	[Min]	[Min]	[% Area]	[µV]	[µV.Min]	[%]
1	UNKNOWN	17.45	17.93	18.91	0.00	82.82	102.0	56.7	82.821
2	UNKNOWN	18.98	19.47	20.20	0.00	17.18	20.4	11.8	17.179
Total						100.00	122.4	68.4	100.000

Supplementary Figure 56. SFC Trace of ***rac*-26**

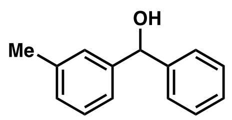

rac-26

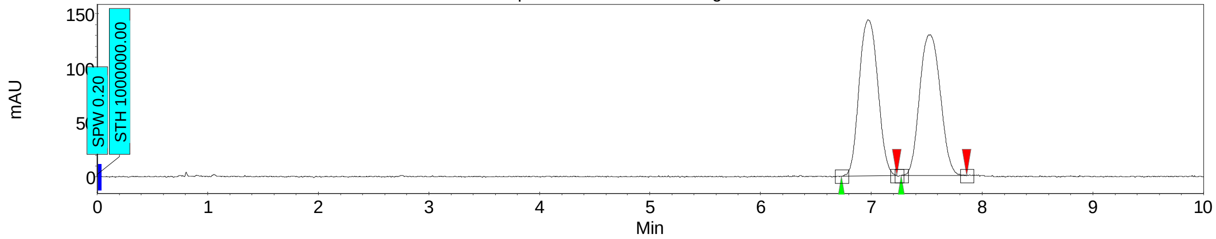

Index	Name	Start	Time	End	RT Offset	Quantity	Height	Area	Area
		[Min]	[Min]	[Min]	[Min]	[% Area]	[µV]	[µV.Min]	[%]
1	UNKNOWN	6.73	6.97	7.23	0.00	50.00	143.1	28.1	50.003
2	UNKNOWN	7.27	7.52	7.86	0.00	50.00	129.3	28.1	49.997
Total						100.00	272.4	56.1	100.000

Supplementary Figure 57. SFC Trace of ***(R)*-26**

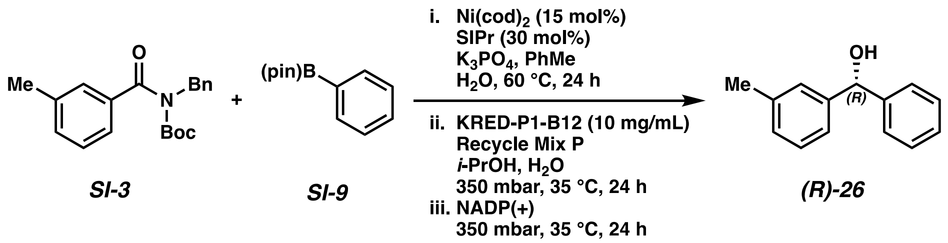

SI-3 ***SI-9*** ***(R)-26***

(67% yield, 87% ee)

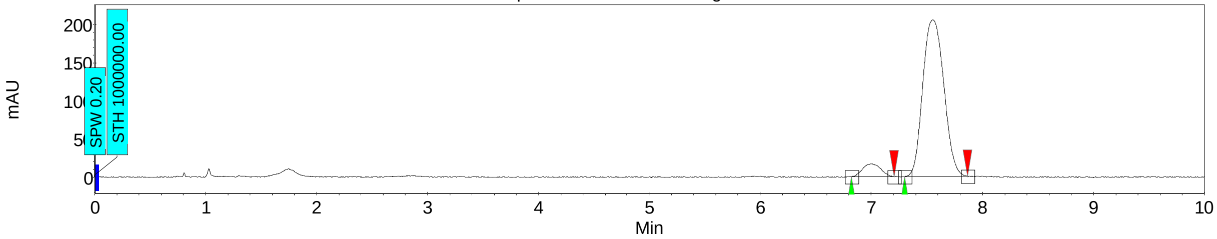

Index	Name	Start	Time	End	RT Offset	Quantity	Height	Area	Area
		[Min]	[Min]	[Min]	[Min]	[% Area]	[µV]	[µV.Min]	[%]
1	UNKNOWN	6.82	7.00	7.21	0.00	6.62	16.9	3.2	6.622
2	UNKNOWN	7.30	7.55	7.86	0.00	93.38	204.6	45.1	93.378
Total						100.00	221.5	48.3	100.000

Supplementary Figure 58. SFC Trace of ***rac*-27**

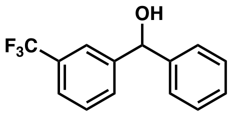

rac-27

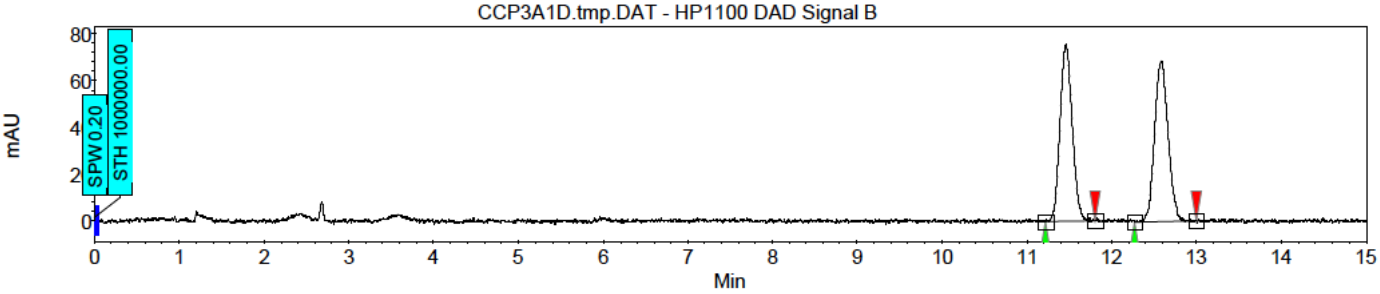

Index	Name	Start	Time	End	RT Offset	Quantity	Height	Area	Area
		[Min]	[Min]	[Min]	[Min]	[% Area]	[µV]	[µV.Min]	[%]
1	UNKNOWN	11.22	11.46	11.81	0.00	49.85	75.3	12.5	49.845
2	UNKNOWN	12.27	12.58	13.00	0.00	50.15	68.7	12.6	50.155
Total						100.00	144.0	25.1	100.000

Supplementary Figure 59. SFC Trace of ***(R)*-27**

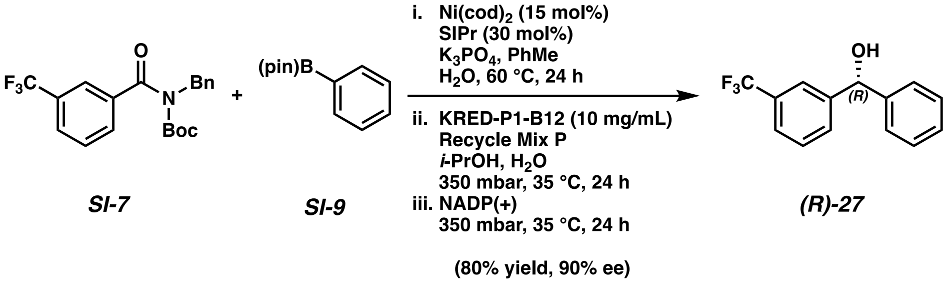

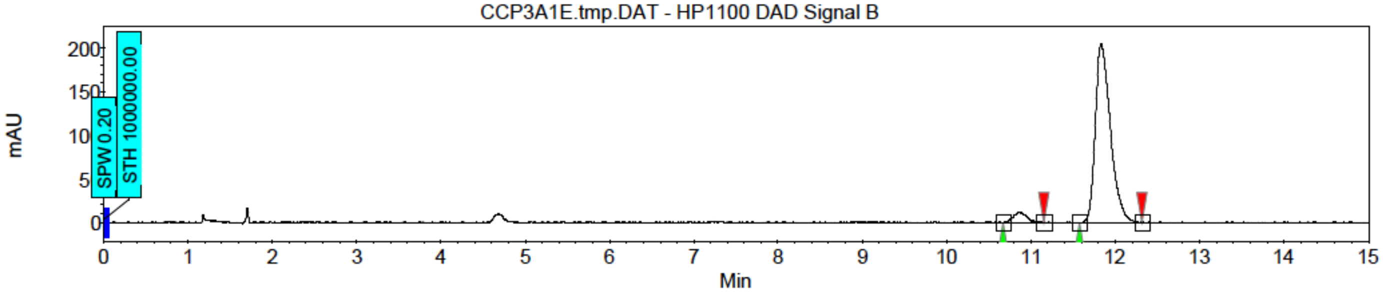

Index	Name	Start	Time	End	RT Offset	Quantity	Height	Area	Area
		[Min]	[Min]	[Min]	[Min]	[% Area]	[µV]	[µV.Min]	[%]
1	UNKNOWN	10.67	10.87	11.15	0.00	4.83	11.4	2.2	4.825
2	UNKNOWN	11.57	11.84	12.32	0.00	95.17	203.9	42.6	95.175
Total						100.00	215.3	44.8	100.000

Supplementary Figure 60. SFC Trace of ***rac*-28**

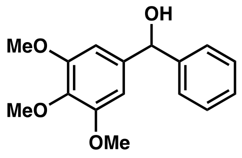

rac-28

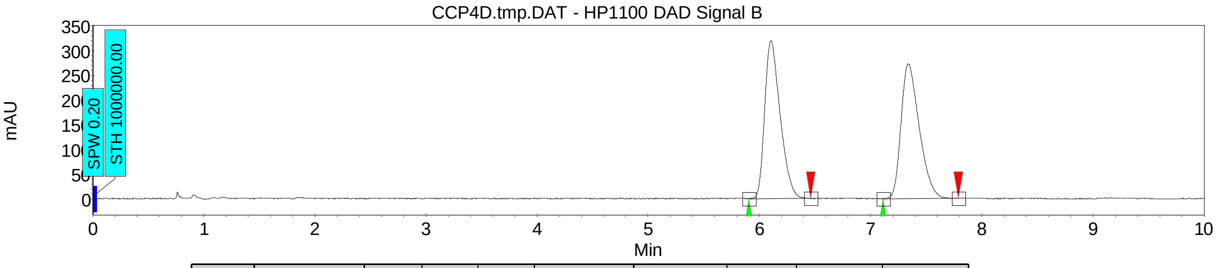

Index	Name	Start	Time	End	RT Offset	Quantity	Height	Area	Area
		[Min]	[Min]	[Min]	[Min]	[% Area]	[µV]	[µV.Min]	[%]
1	UNKNOWN	5.91	6.11	6.46	0.00	50.03	320.1	51.8	50.035
2	UNKNOWN	7.12	7.34	7.79	0.00	49.97	273.1	51.8	49.965
Total						100.00	593.2	103.6	100.000

Supplementary Figure 61. SFC Trace of ***(R)*-28**

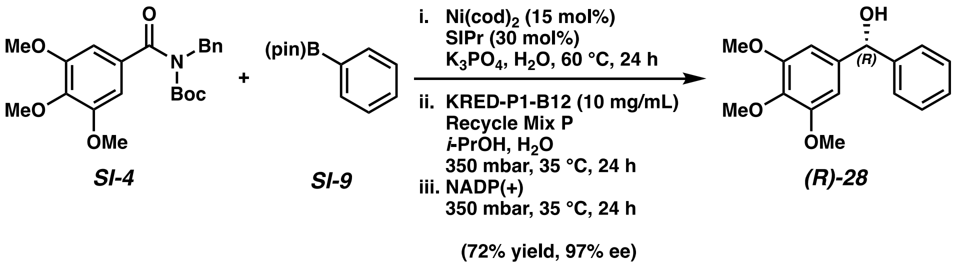

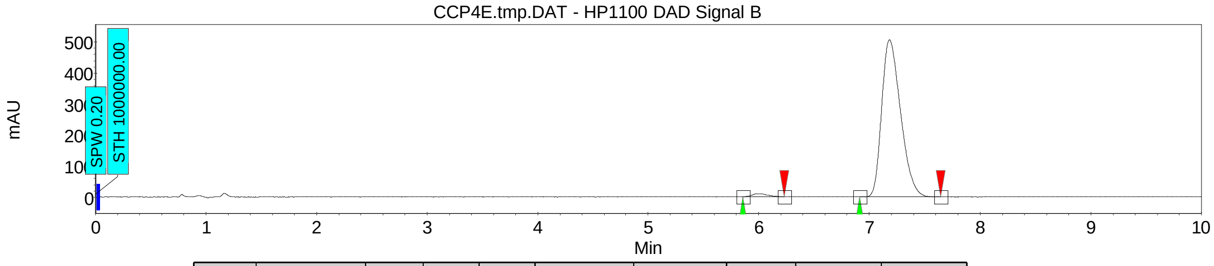

Index	Name	Start	Time	End	RT Offset	Quantity	Height	Area	Area
		[Min]	[Min]	[Min]	[Min]	[% Area]	[µV]	[µV.Min]	[%]
1	UNKNOWN	5.85	6.01	6.23	0.00	1.63	10.0	1.7	1.634
2	UNKNOWN	6.91	7.18	7.64	0.00	98.37	507.2	100.5	98.366
Total						100.00	517.2	102.2	100.000

Supplementary Figure 62. SFC Trace of ***(S)*-27**

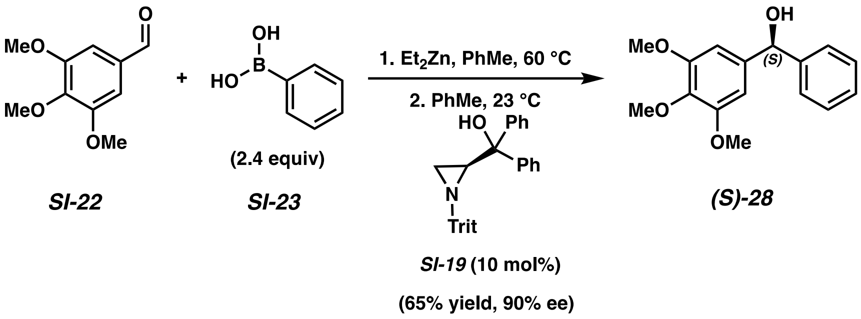

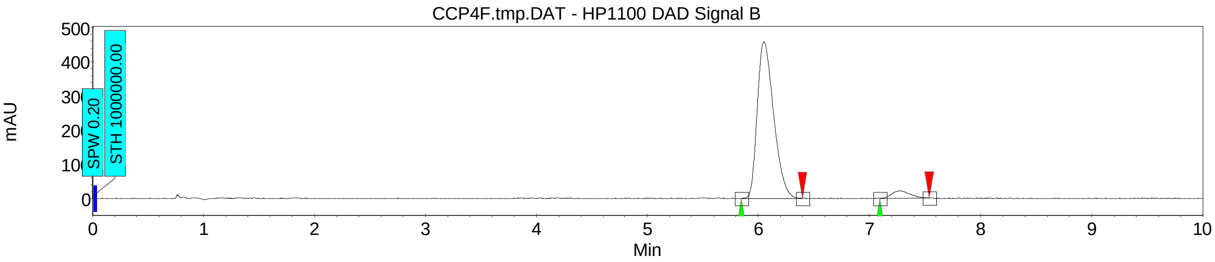

Index	Name	Start	Time	End	RT Offset	Quantity	Height	Area	Area
		[Min]	[Min]	[Min]	[Min]	[% Area]	[µV]	[µV.Min]	[%]
1	UNKNOWN	5.85	6.05	6.40	0.00	94.92	458.4	78.2	94.920
2	UNKNOWN	7.09	7.27	7.54	0.00	5.08	21.9	4.2	5.080
Total						100.00	480.3	82.4	100.000

Supplementary Figure 63. SFC Trace of ***rac*-29**

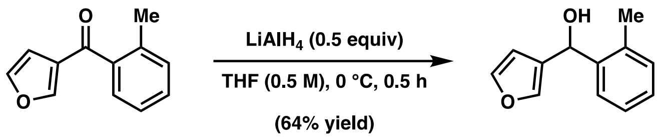

SI-17 ***rac-29***

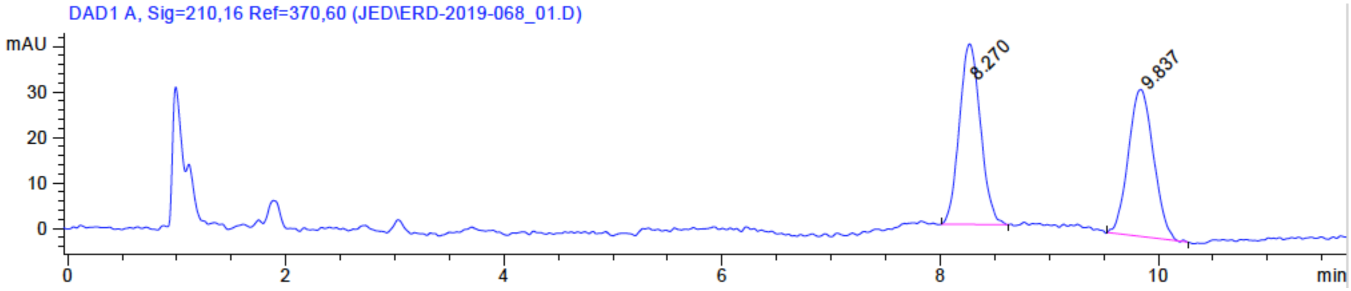

Signal 1: DAD1 A, Sig=210,16 Ref=370,60

Peak #	RetTime [min]	Type	Width [min]	Area [mAU*s]	Height [mAU]	Area %
1	8.270	BB	0.2091	534.61139	39.69661	50.8078
2	9.837	BB	0.2450	517.61230	32.28548	49.1922

Supplementary Figure 64. SFC Trace of ***(S)*-29**

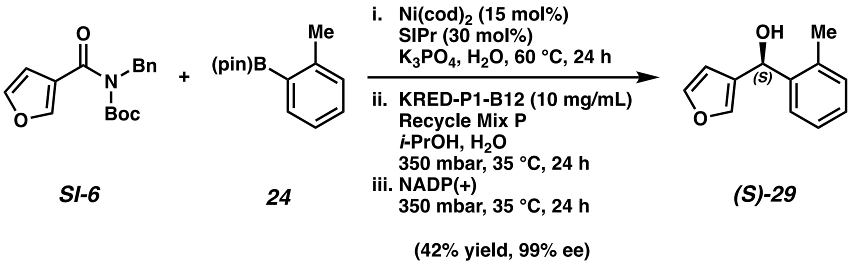

SI-6 ***24*** ***(S)-29***

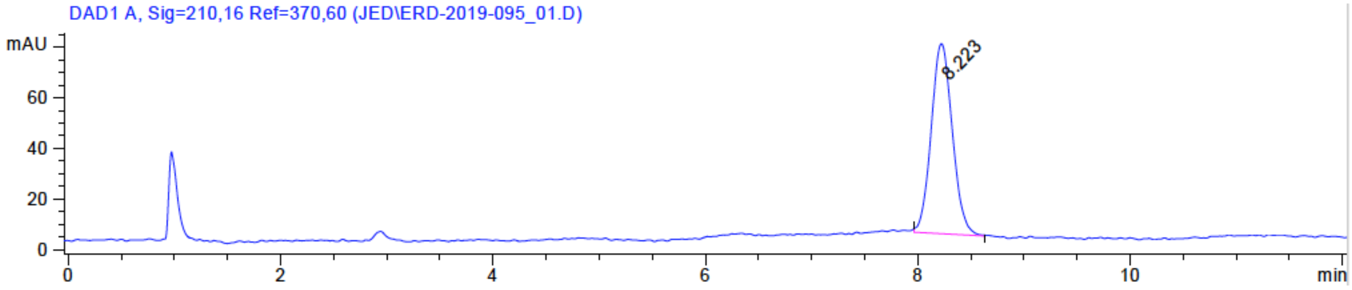

Signal 1: DAD1 A, Sig=210,16 Ref=370,60

Peak #	RetTime [min]	Type	Width [min]	Area [mAU*s]	Height [mAU]	Area %
1	8.223	BB	0.2111	1037.91272	75.17302	100.0000

Supplementary Figure 65. SFC Trace of ***rac*-21**

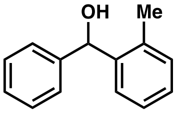

***rac*-21**

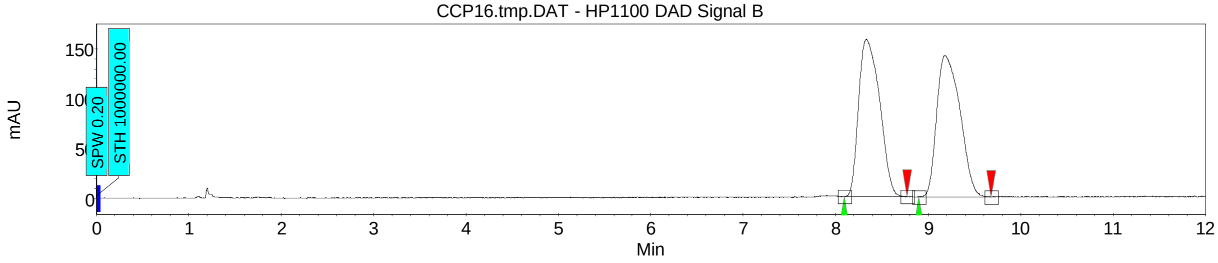

Index	Name	Start	Time	End	RT Offset	Quantity	Height	Area	Area
		[Min]	[Min]	[Min]	[Min]	[% Area]	[μV]	[μV.Min]	[%]
1	UNKNOWN	8.09	8.33	8.77	0.00	50.05	157.1	41.3	50.050
2	UNKNOWN	8.90	9.18	9.68	0.00	49.95	141.4	41.2	49.950
Total						100.00	298.5	82.5	100.000

Supplementary Figure 66. SFC Trace of ***(R)*-21**

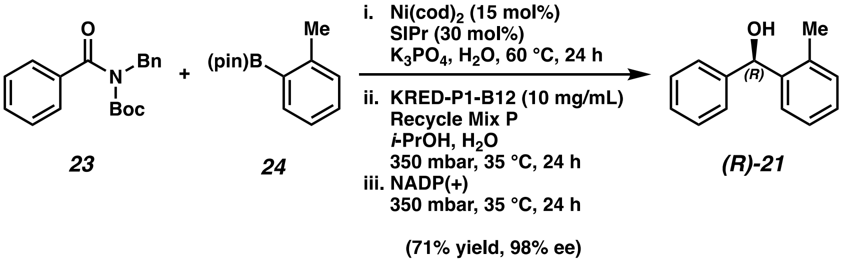

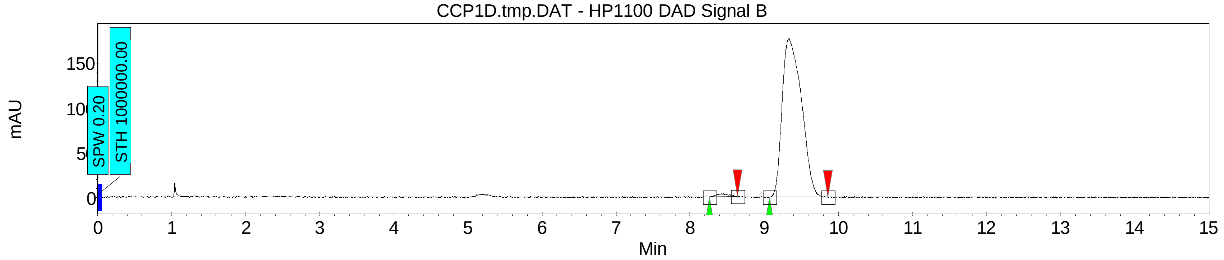

Index	Name	Start	Time	End	RT Offset	Quantity	Height	Area	Area
		[Min]	[Min]	[Min]	[Min]	[% Area]	[μV]	[μV.Min]	[%]
1	UNKNOWN	8.26	8.43	8.64	0.00	1.15	3.1	0.6	1.147
2	UNKNOWN	9.07	9.33	9.86	0.00	98.85	176.0	53.2	98.853
Total						100.00	179.1	53.9	100.000

Supplementary Figure 67. SFC Trace of ***(S)*-21**

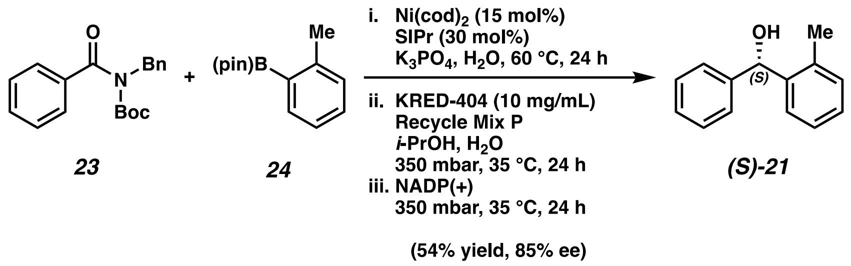

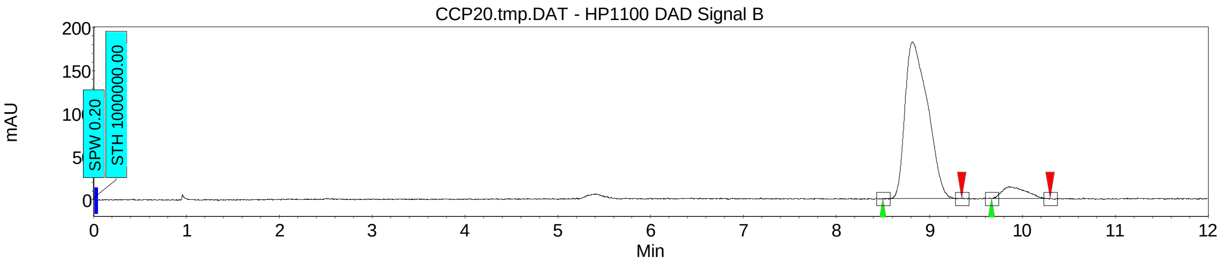

Index	Name	Start	Time	End	RT Offset	Quantity	Height	Area	Area
		[Min]	[Min]	[Min]	[Min]	[% Area]	[µV]	[µV.Min]	[%]
1	UNKNOWN	8.50	8.82	9.35	0.00	92.64	181.6	51.5	92.637
2	UNKNOWN	9.67	9.86	10.30	0.00	7.36	13.6	4.1	7.363
Total						100.00	195.1	55.6	100.000

Supplementary Figure 68. SFC Trace of ***rac*-30**

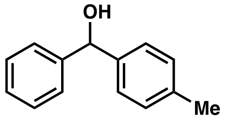

rac-30

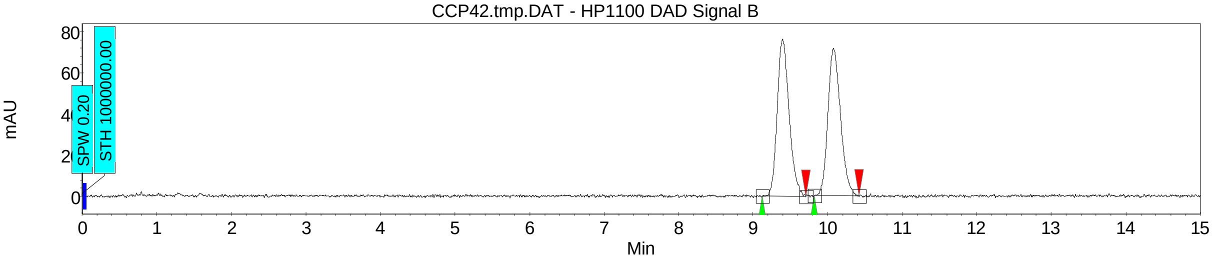

Index	Name	Start	Time	End	RT Offset	Quantity	Height	Area	Area
		[Min]	[Min]	[Min]	[Min]	[% Area]	[µV]	[µV.Min]	[%]
1	UNKNOWN	9.13	9.40	9.71	0.00	50.00	76.1	14.1	50.000
2	UNKNOWN	9.82	10.08	10.43	0.00	50.00	71.5	14.1	50.000
Total						100.00	147.6	28.1	100.000

Supplementary Figure 69. SFC Trace of ***(R)*-30**

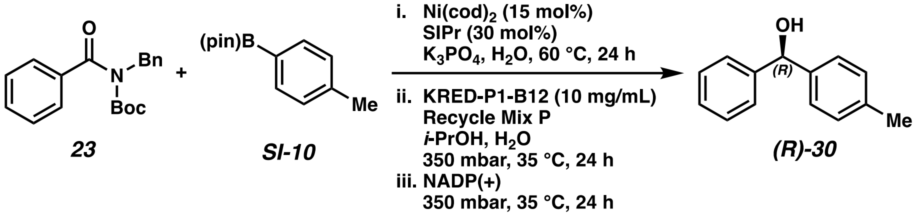

(81% yield, 93% ee)

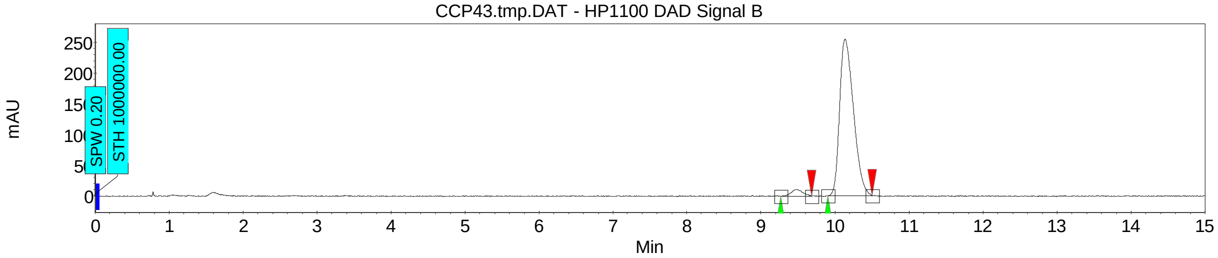

Index	Name	Start	Time	End	RT Offset	Quantity	Height	Area	Area
		[Min]	[Min]	[Min]	[Min]	[% Area]	[µV]	[µV.Min]	[%]
1	UNKNOWN	9.27	9.49	9.69	0.00	3.45	10.9	2.0	3.452
2	UNKNOWN	9.91	10.14	10.51	0.00	96.55	254.5	55.1	96.548
Total						100.00	265.4	57.1	100.000

Supplementary Figure 70. SFC Trace of ***rac*-22**

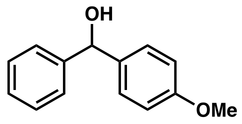

rac-22

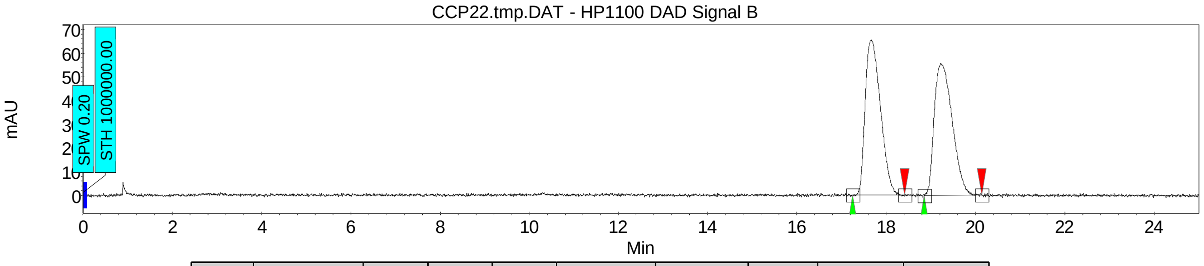

Index	Name	Start	Time	End	RT Offset	Quantity	Height	Area	Area
		[Min]	[Min]	[Min]	[Min]	[% Area]	[µV]	[µV.Min]	[%]
1	UNKNOWN	17.25	17.67	18.41	0.00	50.12	65.4	26.0	50.119
2	UNKNOWN	18.85	19.22	20.14	0.00	49.88	55.7	25.9	49.881
Total						100.00	121.1	51.8	100.000

Supplementary Figure 71. SFC Trace of ***(R)*-22**

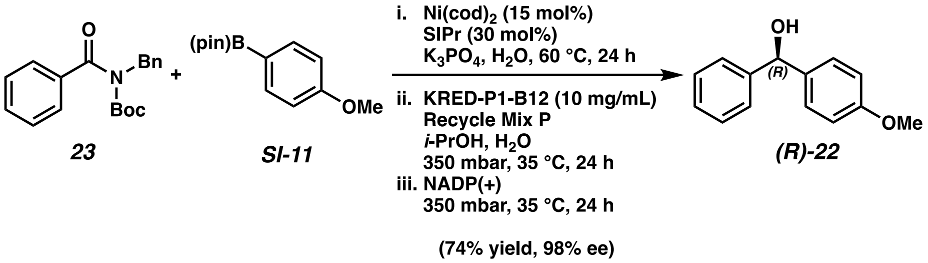

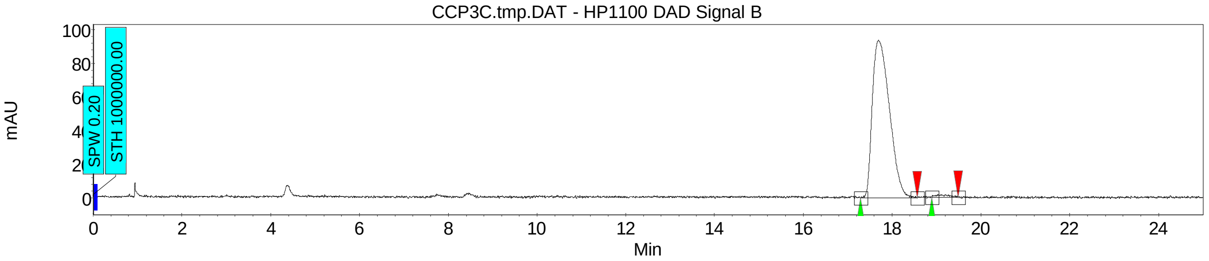

Index	Name	Start	Time	End	RT Offset	Quantity	Height	Area	Area
		[Min]	[Min]	[Min]	[Min]	[% Area]	[µV]	[µV.Min]	[%]
1	UNKNOWN	17.29	17.70	18.57	0.00	98.88	94.0	42.0	98.875
2	UNKNOWN	18.89	19.26	19.49	0.00	1.12	1.5	0.5	1.125
Total						100.00	95.5	42.5	100.000

Supplementary Figure 72. SFC Trace of ***rac*-31**

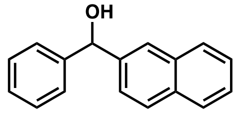

***rac*-31**

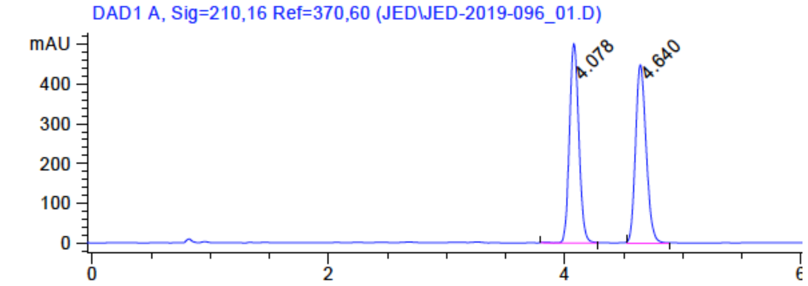

Signal 1: DAD1 A, Sig=210,16 Ref=370,60

Peak #	RetTime [min]	Type	Width [min]	Area [mAU*s]	Height [mAU]	Area %
1	4.078	BB	0.0864	2770.84009	502.07462	49.7535
2	4.640	BB	0.0966	2798.29565	449.76782	50.2465

Supplementary Figure 73. SFC Trace of ***(R)*-31**

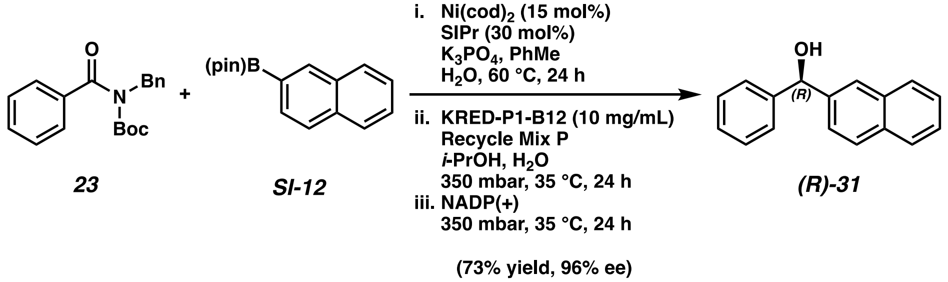

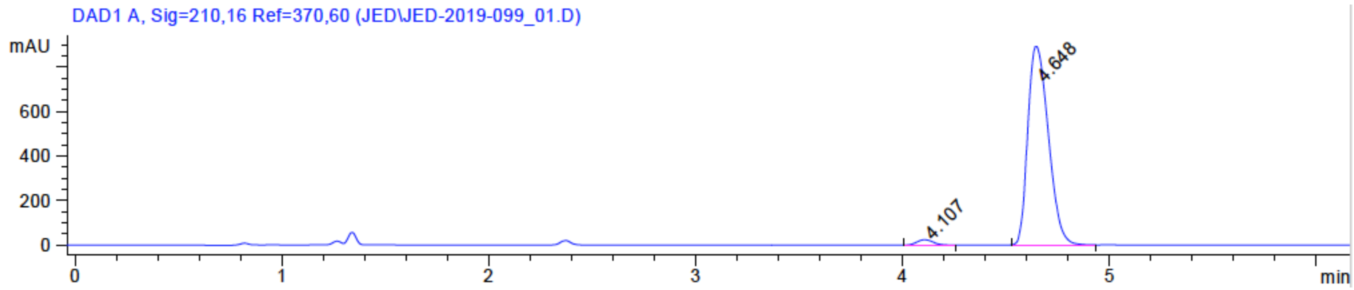

Signal 1: DAD1 A, Sig=210,16 Ref=370,60

Peak #	RetTime [min]	Type	Width [min]	Area [mAU*s]	Height [mAU]	Area %
1	4.107	BB	0.0837	130.76057	24.71537	2.0722
2	4.648	BB	0.1106	6179.55029	894.60040	97.9278

Supplementary Figure 74. SFC Trace of ***rac*-32**

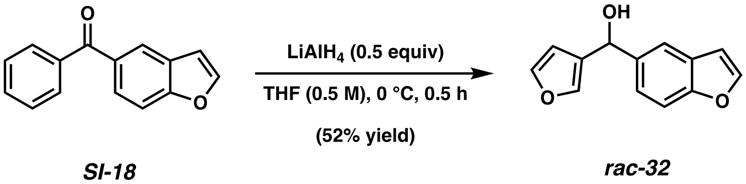

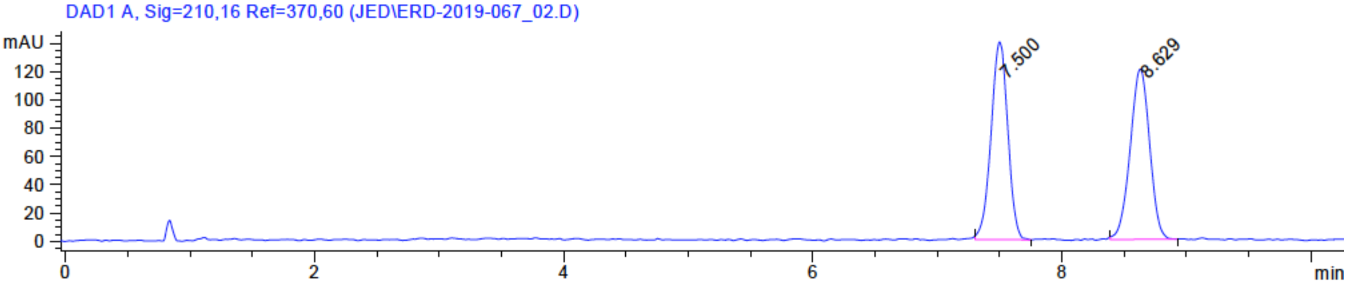

Signal 1: DAD1 A, Sig=210,16 Ref=370,60

Peak #	RetTime [min]	Type	Width [min]	Area [mAU*s]	Height [mAU]	Area %
1	7.500	BB	0.1453	1310.44800	139.86392	50.0445
2	8.629	BB	0.1687	1308.11926	120.48936	49.9555

Supplementary Figure 75. SFC Trace of ***(R)*-32**

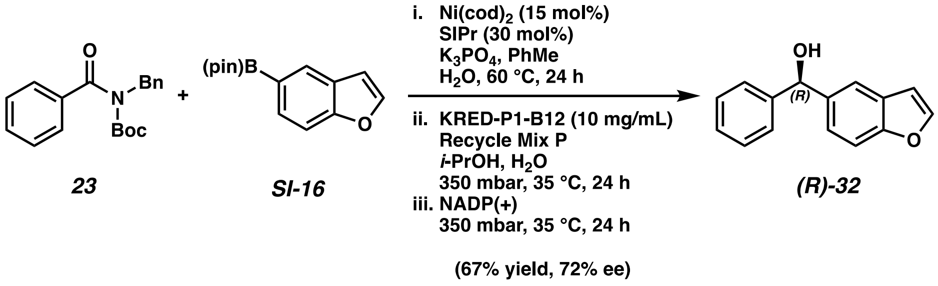

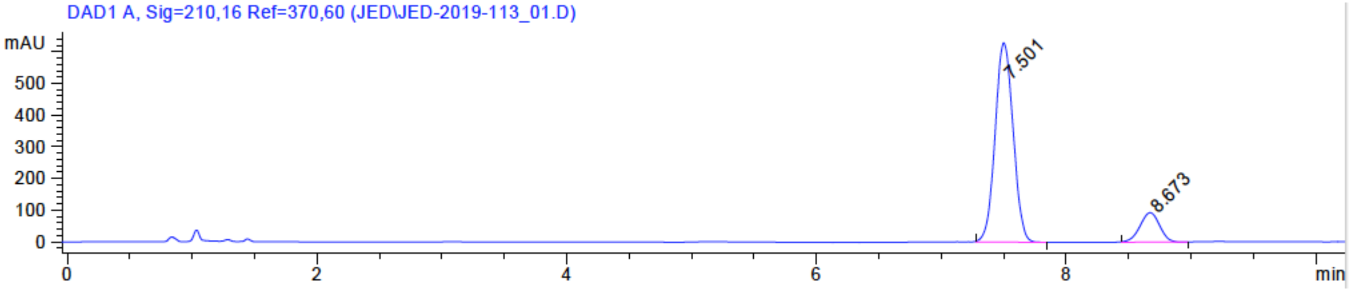

Signal 1: DAD1 A, Sig=210,16 Ref=370,60

Peak #	RetTime [min]	Type	Width [min]	Area [mAU*s]	Height [mAU]	Area %
1	7.501	BB	0.1592	6324.12988	629.74841	86.1817
2	8.673	BB	0.1707	1014.00287	93.35793	13.8183

Supplementary Figure 76. SFC Trace of ***rac*-33**

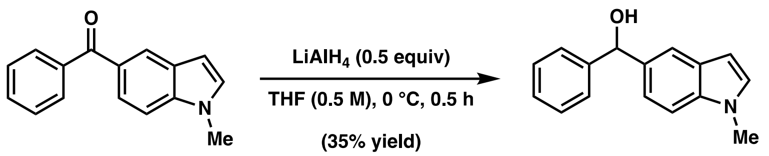

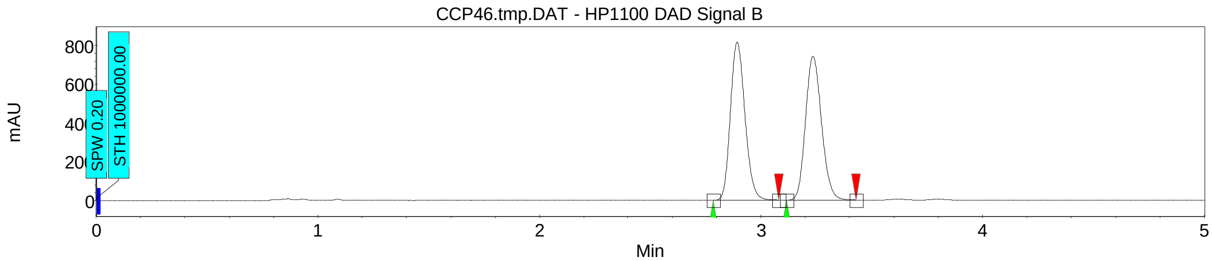

Index	Name	Start	Time	End	RT Offset	Quantity	Height	Area	Area
		[Min]	[Min]	[Min]	[Min]	[% Area]	[µV]	[µV.Min]	[%]
1	UNKNOWN	2.78	2.89	3.08	0.00	49.98	813.4	61.9	49.982
2	UNKNOWN	3.12	3.24	3.43	0.00	50.02	740.5	61.9	50.018
Total						100.00	1553.9	123.8	100.000

Supplementary Figure 77. SFC Trace of ***(R)*-33**

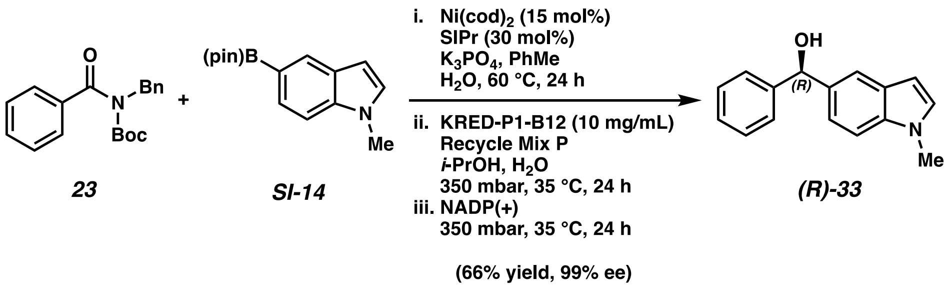

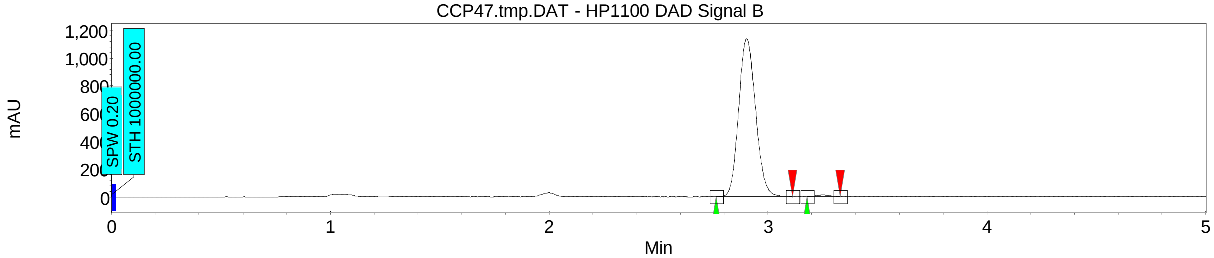

Index	Name	Start	Time	End	RT Offset	Quantity	Height	Area	Area
		[Min]	[Min]	[Min]	[Min]	[% Area]	[µV]	[µV.Min]	[%]
1	UNKNOWN	2.76	2.90	3.11	0.00	99.26	1135.8	101.8	99.258
2	UNKNOWN	3.18	3.25	3.33	0.00	0.74	9.6	0.8	0.742
Total						100.00	1145.4	102.6	100.000

Supplementary Figure 78. SFC Trace of ***(R)*-33**

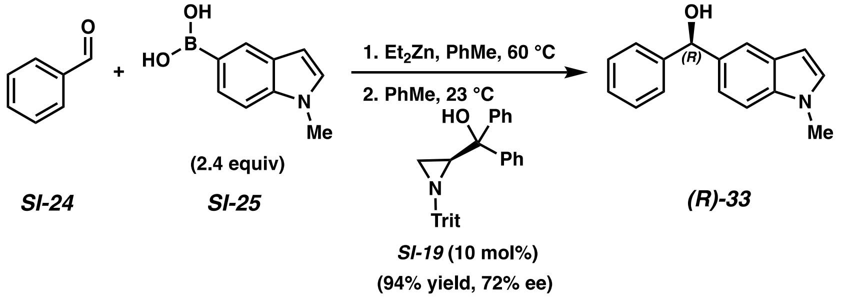

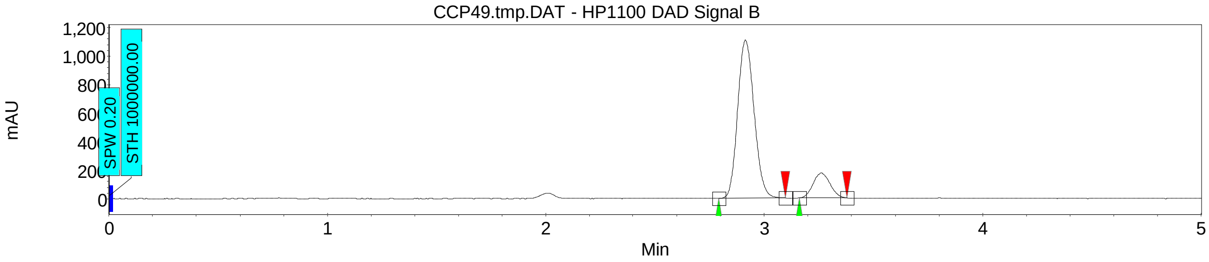

Index	Name	Start	Time	End	RT Offset	Quantity	Height	Area	Area
		[Min]	[Min]	[Min]	[Min]	[% Area]	[µV]	[µV.Min]	[%]
1	UNKNOWN	2.79	2.92	3.10	0.00	86.03	1108.9	96.9	86.030
2	UNKNOWN	3.16	3.26	3.38	0.00	13.97	176.7	15.7	13.970
Total						100.00	1285.6	112.6	100.000
